# Supplementary material for: Synthesis and Elimination Pathways of 1-Methanesulfonyl-1,2-dihydroquinoline Sulfonamides
Source: Molecules. 2023 Apr 5;28(7):3256. doi: 10.3390/molecules28073256 (PMC10096818; doi:10.3390/molecules28073256)
Supplement: Supplementary file 1 [file molecules-28-03256-s001.zip › molecules-2321702-supplementary.pdf]

Synthesis and Elimination Pathways of 1-Methane-sulfonyl-1,2-Dihydroquinoline Sulfonamides

Ebenezer Ametsetor, Kwabena Fobi and Richard A. Bunce*

*Correspondence: rab@okstate.edu;

Compound **Page**

^{1}H NMR for (±)-ethyl 2-(acetoxy(2-fluoro-5-nitrophenyl)methyl)acrylate (**13**)....3
^{13}C NMR for (±)-ethyl 2-(acetoxy(2-fluoro-5-nitrophenyl)methyl)acrylate (**13**) 3
^{1}H NMR for (±)-ethyl 2-(acetoxy(5-cyano-2-fluorophenyl)methyl)acrylate (**14**) 4
^{13}C NMR for (±)-ethyl 2-(acetoxy(5-cyano-2-fluorophenyl)methyl)acrylate (**14**) 4
^{1}H NMR for (±)-ethyl 2-(acetoxy(2-fluoro-5-(trifluoromethyl)phenyl)methyl)acrylate (**15**)....5
^{13}C NMR for (±)-ethyl 2-(acetoxy(2-fluoro-5-(trifluoromethyl)phenyl)methyl)acrylate (**15**)....5
^{1}H NMR for (±)-ethyl 2-(acetoxy(5-chloro-2-fluorophenyl)methyl)acrylate (**16**)....6
^{13}C NMR for (±)-ethyl 2-(acetoxy(5-chloro-2-fluorophenyl)methyl)acrylate (**16**)....6
^{1}H NMR for (±)-ethyl 2-(acetoxy(2,4,5-trifluorophenyl)methyl)acrylate (**17**)....7
^{13}C NMR for (±)-ethyl 2-(acetoxy(2,4,5-trifluorophenyl)methyl)acrylate (**17**) 7
^{1}H NMR for (±)-ethyl (±)-2-(acetoxy(2-fluoropyridin-3-yl)methyl)acrylate (**18**)....8
^{13}C NMR for (±)-ethyl 2-(acetoxy(2-fluoropyridin-3-yl)methyl)acrylate (**18**)....8
^{1}H NMR for (±)-2-cyano-1-(2-fluoro-5-nitrophenyl)allyl acetate (**19**) 9
^{13}C NMR for (±)-2-cyano-1-(2-fluoro-5-nitrophenyl)allyl acetate (**19**) 9
^{1}H NMR for (±)-2-cyano-1-(5-cyano-2-fluorophenyl)allyl acetate (**20**) 10
^{13}C NMR for (±)-2-cyano-1-(5-cyano-2-fluorophenyl)allyl acetate (**20**) 10
^{1}H NMR for (±)-2-cyano-1-(2-fluoro-5-(trifluoromethyl)phenyl)allyl acetate (**21**)....11
^{13}C NMR for (±)-2-cyano-1-(2-fluoro-5-(trifluoromethyl)phenyl)allyl acetate (**21**)....11
^{1}H NMR for (±)-1-(5-chloro-2-fluorophenyl)-2-cyanoallyl acetate (**22**) 12
^{13}C NMR for (±)-1-(5-chloro-2-fluorophenyl)-2-cyanoallyl acetate (**22**) 12
^{1}H NMR for (±)-1-(2-fluoro-5-nitrophenyl)-2-methylene-3-oxobutyl acetate (**23**)....13
^{13}C NMR for (±)-1-(2-fluoro-5-nitrophenyl)-2-methylene-3-oxobutyl acetate (**23**) 13
^{1}H NMR for (±)-1-(5-cyano-2-fluorophenyl)-2-methylene-3-oxobutyl acetate (**24**)....14
^{13}C NMR for (±)-1-(5-cyano-2-fluorophenyl)-2-methylene-3-oxobutyl acetate (**24**)....14
^{1}H NMR for (±)-1-(2-fluoro-5-(trifluoromethyl) phenyl)-2-methylene-3-oxobutyl acetate (**25**)....15
^{13}C NMR for (±)-1-(2-fluoro-5-(trifluoromethyl) phenyl)-2-methylene-3-oxobutyl acetate (**25**) 15
^{1}H NMR for (±)-2-methylene-3-oxo-1-(2,4,5-trifluorophenyl)butyl acetate (**26**)....16
^{13}C NMR for (±)-2-methylene-3-oxo-1-(2,4,5-trifluorophenyl)butyl acetate (**26**) 16
^{1}H NMR for (±)-1-(2-fluoropyridin-3-yl)-2-methylene-3-oxobutyl acetate (**27**) 17
^{13}C NMR for (±)-1-(2-fluoropyridin-3-yl)-2-methylene-3-oxobutyl acetate (**27**) 17

^{1}H NMR for ethyl 1-(methylsulfonyl)-6-nitro-1, 2-dihydroquinoline-3-carboxylate (**28-Ms**) 18
^{13}C NMR for ethyl 1-(methylsulfonyl)-6-nitro-1, 2-dihydroquinoline-3-carboxylate (**28-Ms**) 18
^{1}H NMR for ethyl 6-nitro-1-tosyl-1,2-dihydroquinoline-3-carboxylate (**28-Ts**) 19
^{13}C NMR for ethyl 6-nitro-1-tosyl-1,2-dihydroquinoline-3-carboxylate (**28-Ts**) 19
^{1}H NMR for ethyl 6-cyano-1-(methylsulfonyl)-1,2-dihydroquinoline-3-carboxylate (**29**) 20
^{13}C NMR for ethyl 6-cyano-1-(methylsulfonyl)-1,2-dihydroquinoline-3-carboxylate (**29**) 20
^{1}H NMR for ethyl 1-(methylsulfonyl)-6-(trifluoromethyl)-1,2-dihydroquinoline-3-carboxylate (**30**) 21
^{13}C NMR for ethyl 1-(methylsulfonyl)-6-(trifluoromethyl)-1,2-dihydroquinoline-3-carboxylate (**30**) 21

^{1}H NMR for ethyl 6-chloro-1-(methylsulfonyl)-1,2-dihydroquinoline-3-carboxylate (**31**) 22
^{13}C NMR for ethyl 6-chloro-1-(methylsulfonyl)-1,2-dihydroquinoline-3-carboxylate (**31**) 22
^{1}H NMR for ethyl 6,7-difluoro-1-(methylsulfonyl)-1,2-dihydroquinoline-3-carboxylate (**32**) 23
^{13}C NMR for ethyl 6,7-difluoro-1-(methylsulfonyl)-1,2-dihydroquinoline-3-carboxylate (**32**) 23
^{1}H NMR for ethyl 1-(methylsulfonyl)-1,2-dihydro-1,8-naphthyridine-3-carboxylate (**33**) 29
^{13}C NMR for ethyl 1-(methylsulfonyl)-1,2-dihydro-1,8-naphthyridine-3-carboxylate (**33**)....29
^{1}H NMR of 1-(methylsulfonyl)-6-nitro-1,2-dihydronaphthalene-3-carbonitrile (**34**) 24
^{13}C NMR of 1-(methylsulfonyl)-6-nitro-1,2-dihydronaphthalene-3-carbonitrile (**34**) 24
^{1}H NMR for 1-(1-(methylsulfonyl)-6-nitro-1,2-dihydroquinolin-3-yl)ethan-1-one (**38**) 25
^{13}C NMR for 1-(1-(methylsulfonyl)-6-nitro-1,2-dihydroquinolin-3-yl)ethan-1-one (**38**) 25
^{1}H NMR of 3-acetyl-1-(methylsulfonyl)-1,2-dihydroquinoline-6-carbonitrile (**39**) 26
^{13}C NMR of 3-acetyl-1-(methylsulfonyl)-1,2-dihydroquinoline-6-carbonitrile (**39**) 26
^{1}H NMR for 1-(1-(methylsulfonyl)-6-(trifluoromethyl)-1,2-dihydroquinolin-3-yl)ethan-1-one (**40**) 27
^{13}C NMR for 1-(1-(methylsulfonyl)-6-(trifluoromethyl)-1,2-dihydroquinolin-3-yl)ethan-1-one (**40**) 27
^{1}H NMR of 1-(6,7-difluoro-1-(methylsulfonyl)-1,2-dihydroquinolin-3-yl)ethan-1-one (**41**) 28
^{13}C NMR of 1-(6,7-difluoro-1-(methylsulfonyl)-1,2-dihydroquinolin-3-yl)ethan-1-one (**41**) 28
^{1}H for 1-(1-(methylsulfonyl)-1,2-dihydro-1,8-naphthyridin-3-yl)ethan-1-one (**42**) 30
^{13}C for 1-(1-(methylsulfonyl)-1,2-dihydro-1,8-naphthyridin-3-yl)ethan-1-one (**42**) 30

^{1}H NMR or ethyl 6-nitroquinoline-3-carboxylate (**43** from **28-Ms**) 31
^{13}C NMR or ethyl 6-nitroquinoline-3-carboxylate (**43** from **28-Ms**)....31
^{1}H NMR for ethyl 6-nitroquinoline-3-carboxylate (**43** from **28-Ts**) 32
^{13}C NMR for ethyl 6-nitroquinoline-3-carboxylate (**43** from **28-Ts**) 32
^{1}H NMR for ethyl 6-cyanoquinoline-3-carboxylate (**44**) 33
^{13}C NMR for ethyl 6-cyanoquinoline-3-carboxylate (**44**)....33
^{1}H NMR for ethyl 6-(trifluoromethyl)quinoline-3-carboxylate (**45**) 34
^{13}C NMR for ethyl 6-(trifluoromethyl)quinoline-3-carboxylate (**45**) 34
^{1}H NMR for ethyl 6-chloroquinoline-3-carboxylate (**46**) 35
^{13}C NMR for ethyl 6-chloroquinoline-3-carboxylate (**46**) 35
^{1}H NMR for 6-nitroquinoline-3-carbonitrile (**48**) 36
^{13}C NMR for 6-nitroquinoline-3-carbonitrile (**48**) 36
^{1}H NMR for ethyl 7-(methylsulfonyl)-6-(phenethylamino)quinoline-3-carboxylate (**49**) 37
^{13}C NMR for ethyl 7-(methylsulfonyl)-6-(phenethylamino)quinoline-3-carboxylate (**49**) 37

X-ray structure elucidation of **49** (CCDC 2246638) 38-49

^{1}H NMR for (±)-ethyl 2-(acetoxy(2-fluoro-5-nitrophenyl) methyl)acrylate (13)

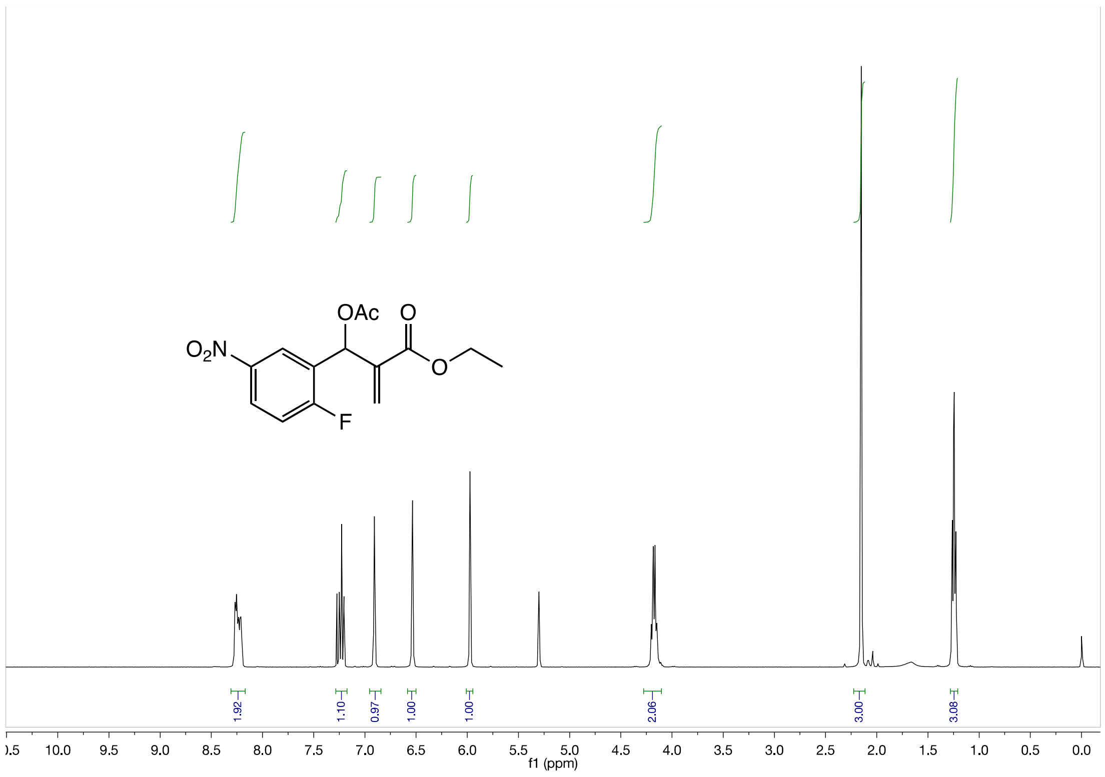

^{13}C NMR for (±)-ethyl 2-(acetoxy(2-fluoro-5-nitrophenyl)methyl)acrylate (13)

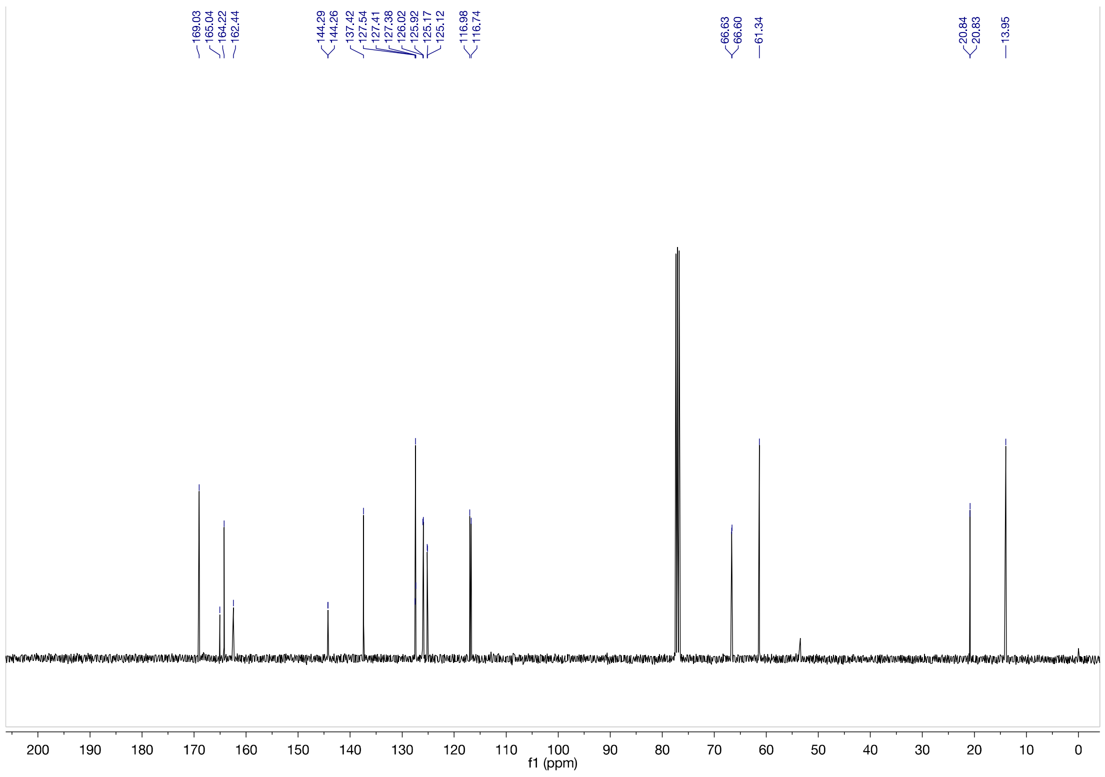

^{1}H NMR for (±)-ethyl 2-(acetoxy(5-cyano-2-fluorophenyl)methyl)acrylate (14)

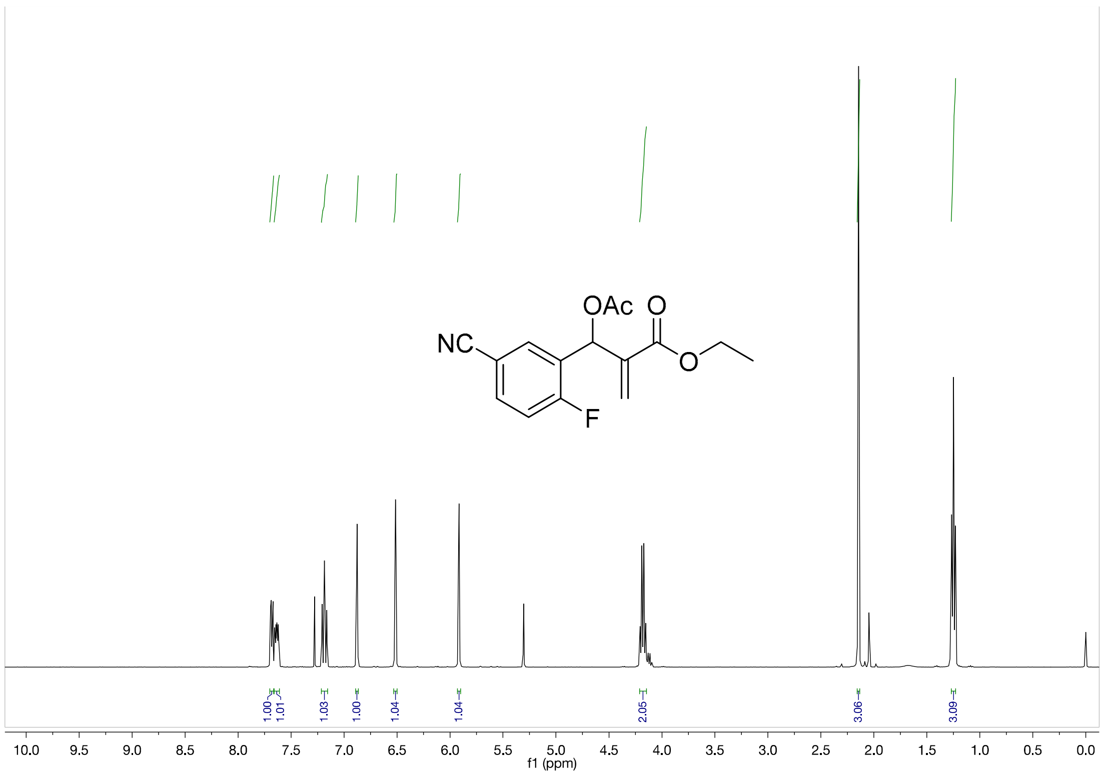

^{13}C NMR for (±)-ethyl 2-(acetoxy(5-cyano-2-fluorophenyl)methyl)acrylate (14)

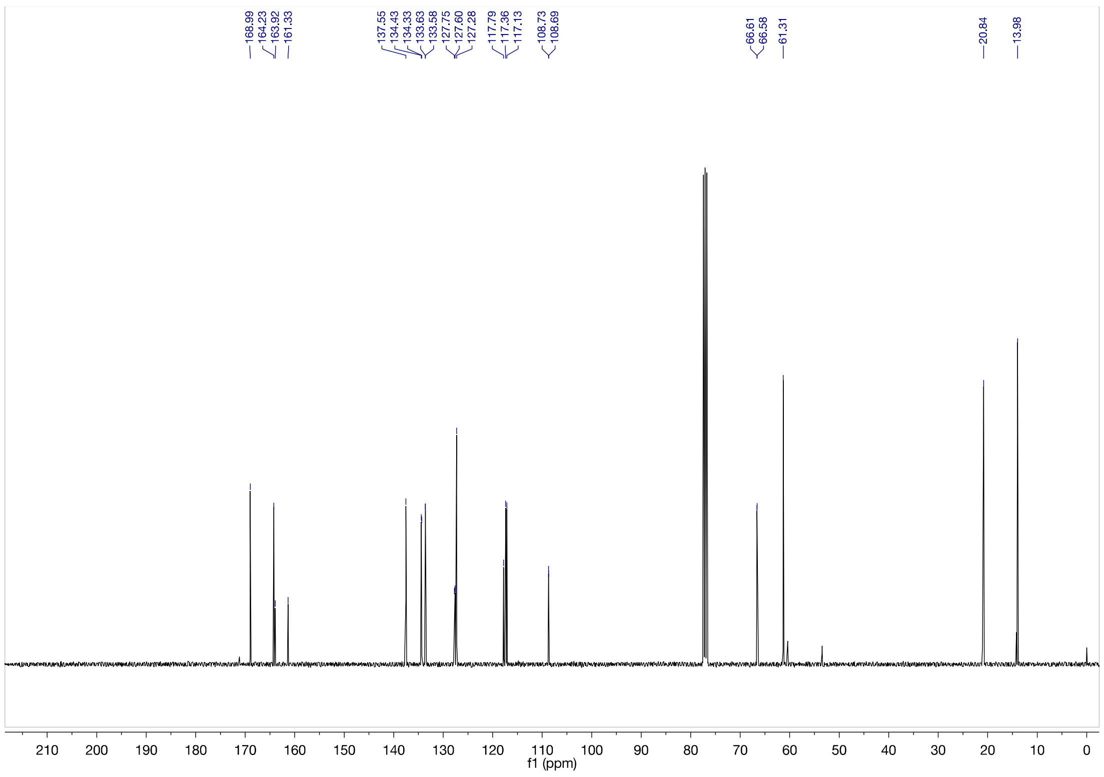

^{1}H NMR for (±)-ethyl 2-(acetoxy(2-fluoro-5-(trifluoromethyl)phenyl)methyl)acrylate (15)

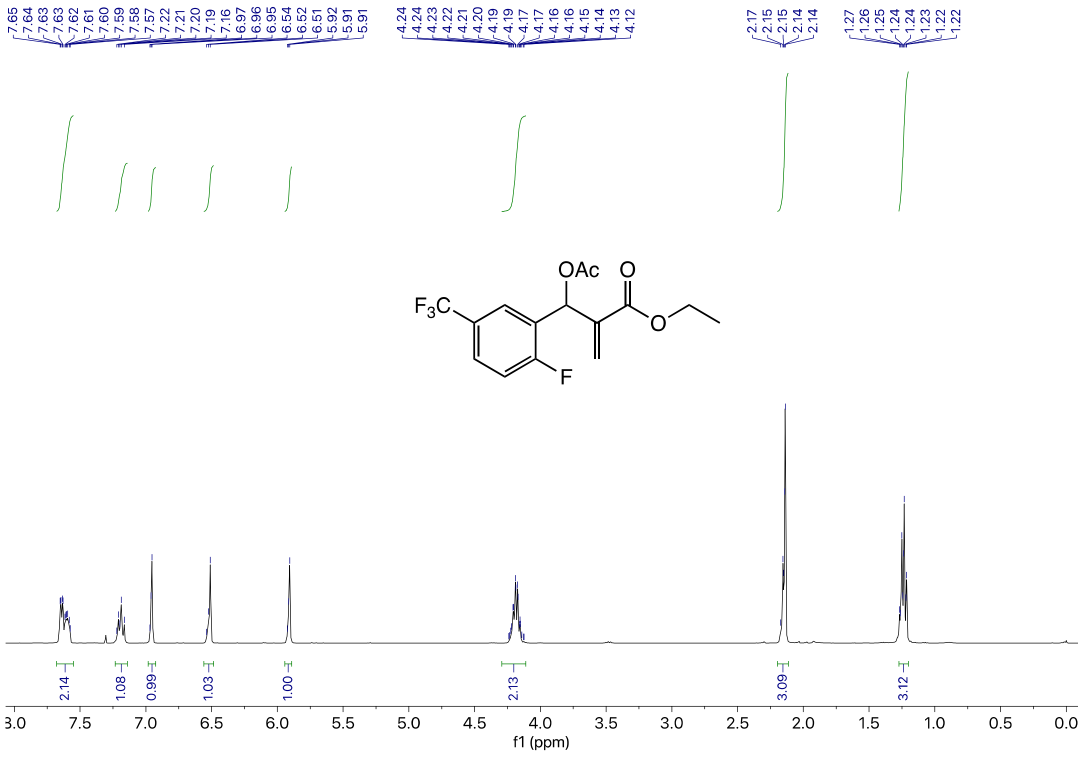

^{13}C NMR for ethyl 2-(acetoxy(2-fluoro-5-(trifluoromethyl) phenyl)methyl)acrylate (15)

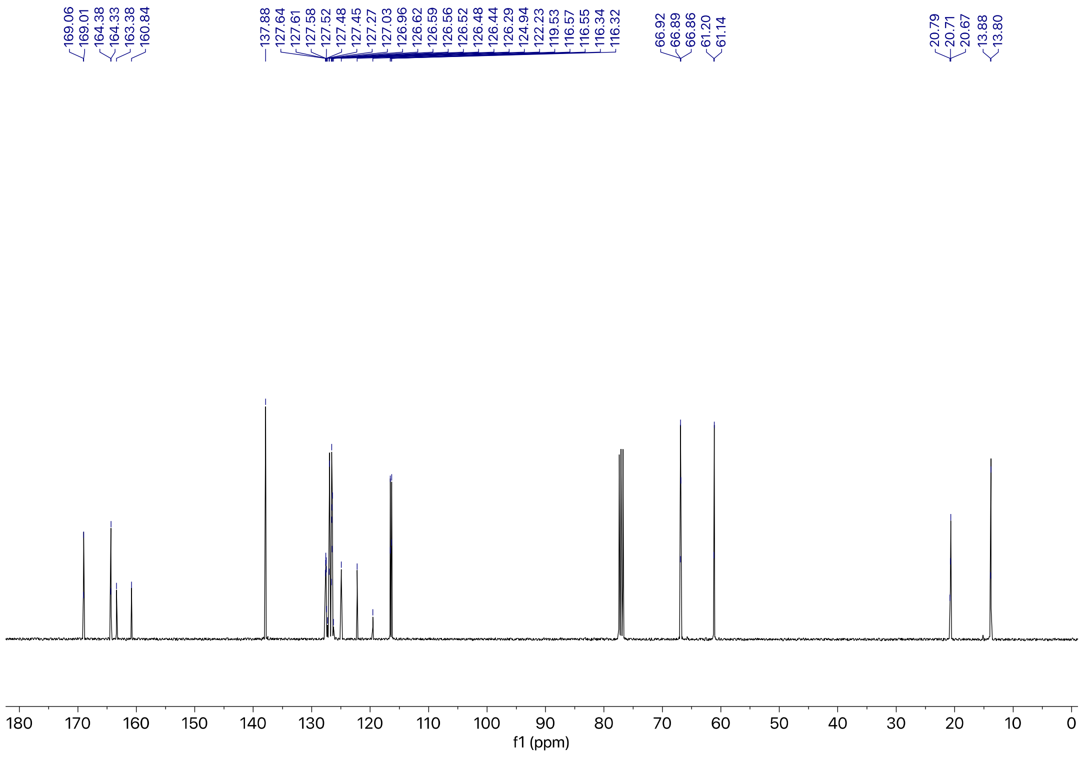

¹H NMR for (±)-ethyl 2-(acetoxy(5-chloro-2-fluorophenyl)methyl)acrylate (16)

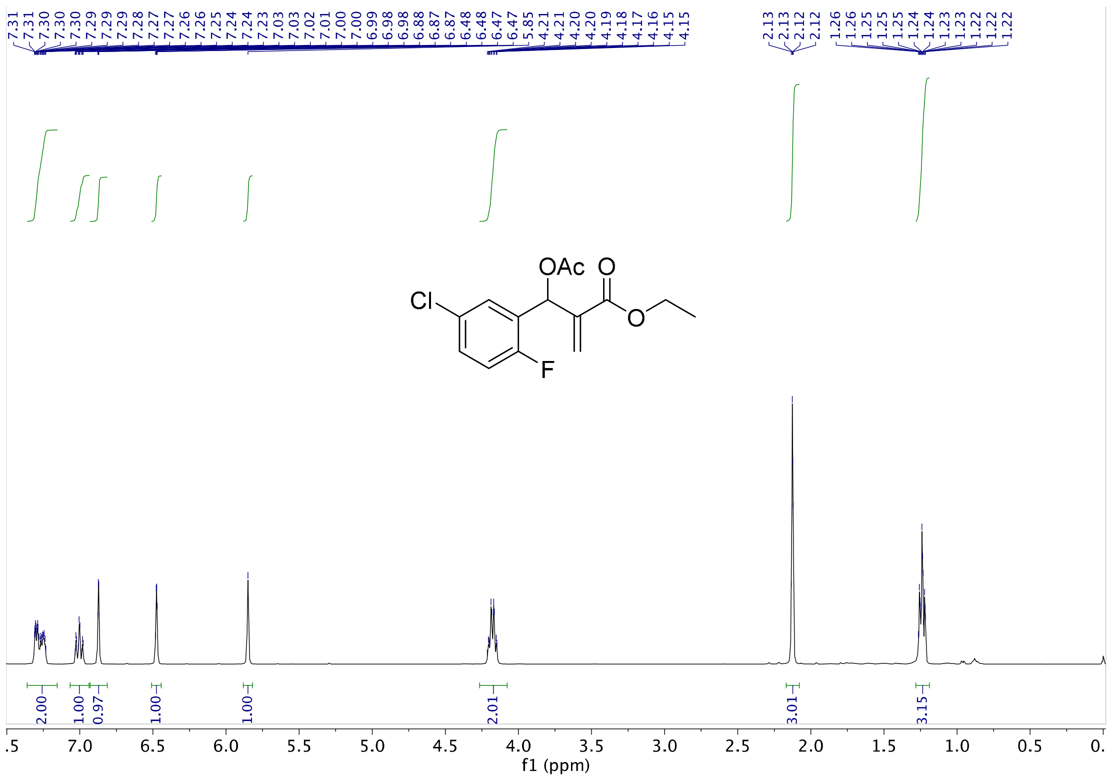

¹³C NMR for (±)-ethyl 2-(acetoxy(5-chloro-2-fluorophenyl)methyl)acrylate (16)

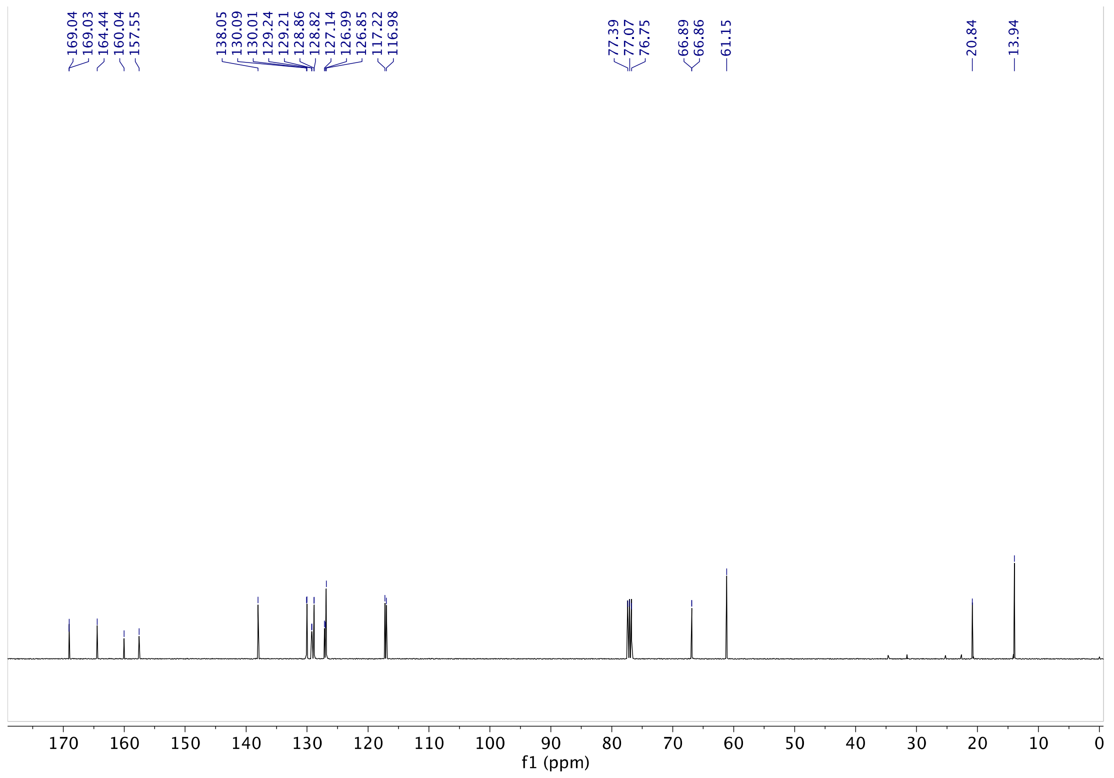

^{1}H NMR for (±)-ethyl 2-(acetoxy(2,4,5-trifluorophenyl)methyl)acrylate (17)

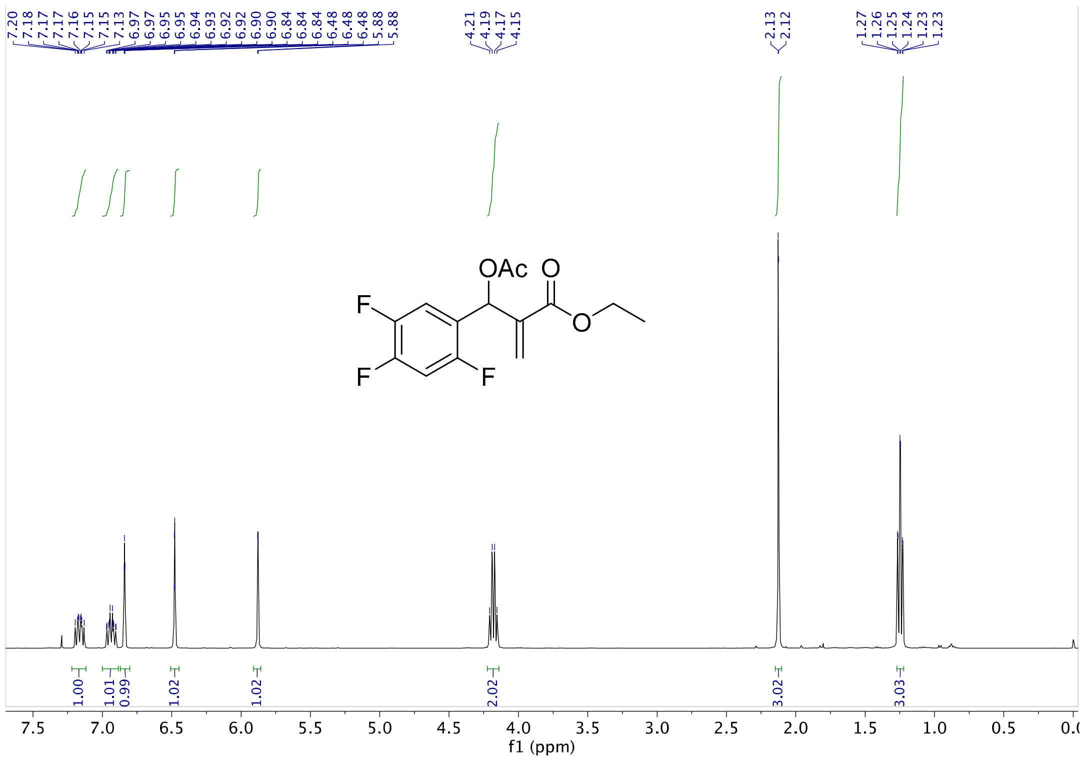

^{13}C NMR for (±)-ethyl 2-(acetoxy (2,4,5-trifluorophenyl)methyl)acrylate (17)

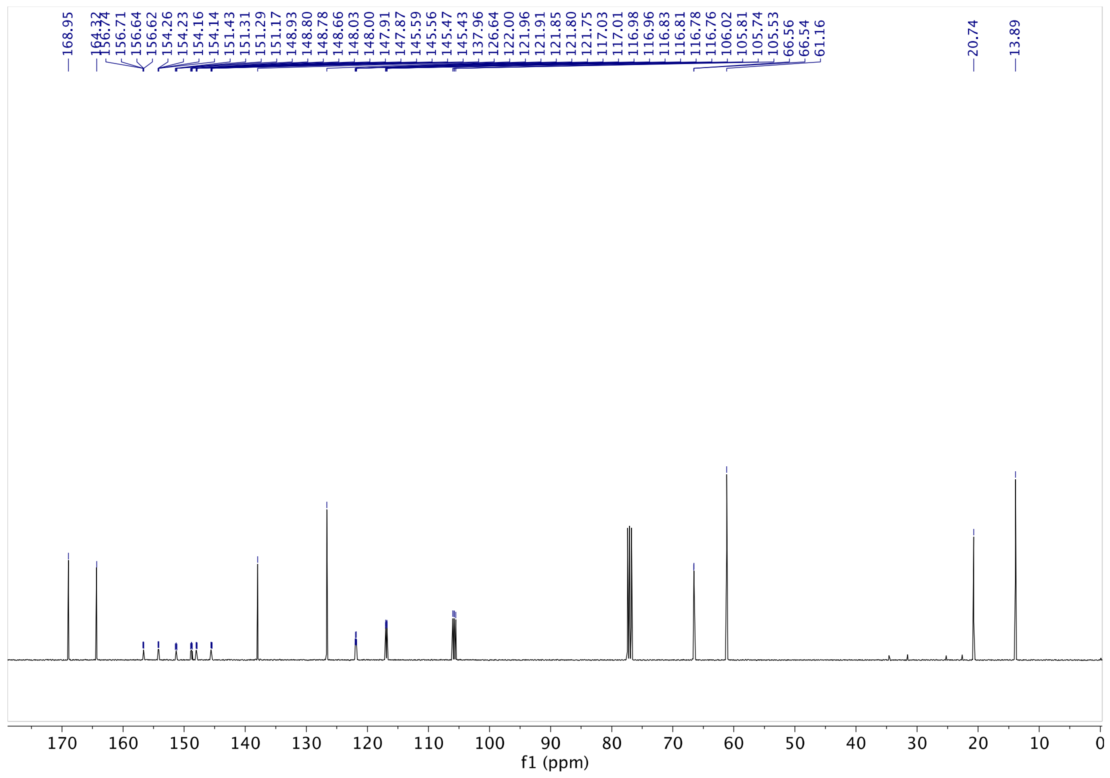

^{1}H NMR for (±)-ethyl 2-(acetoxy(2-fluoropyridin-3-yl)methyl)acrylate (18)

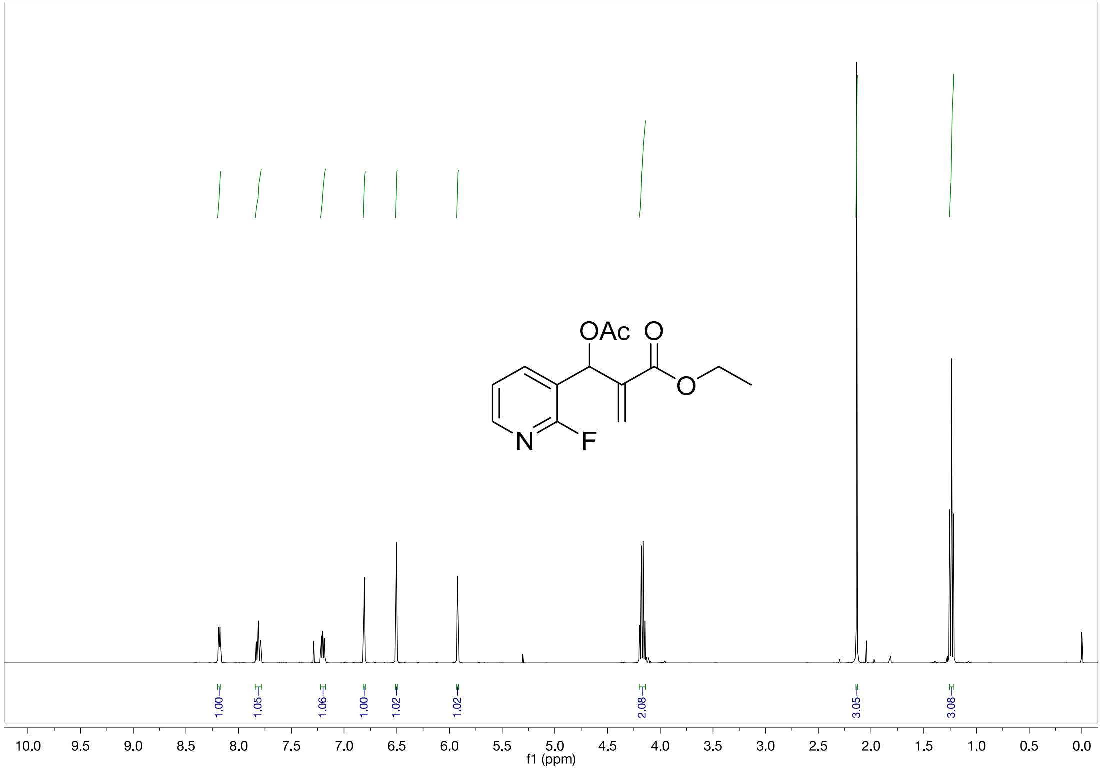

^{13}C NMR for (±)-ethyl 2-(acetoxy(2-fluoropyridin-3-yl)methyl)acrylate (18)

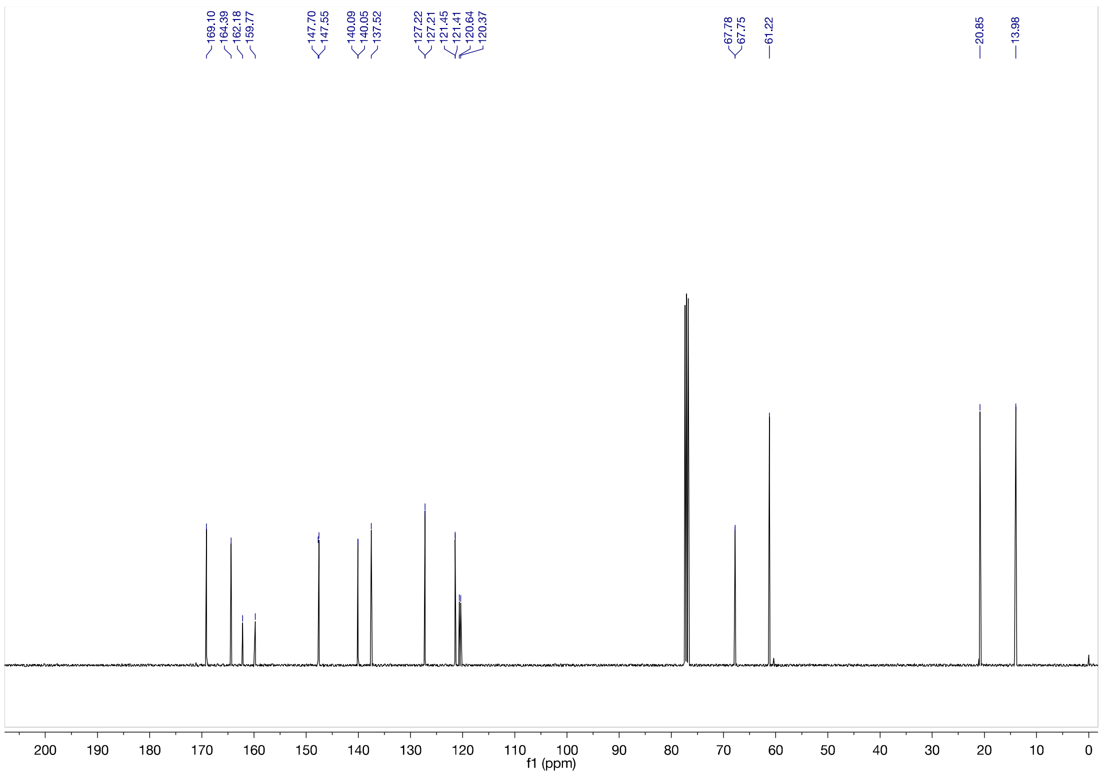

^{1}H NMR for (±)-2-cyano-1-(2-fluoro-5-nitrophenyl)allyl acetate (19)

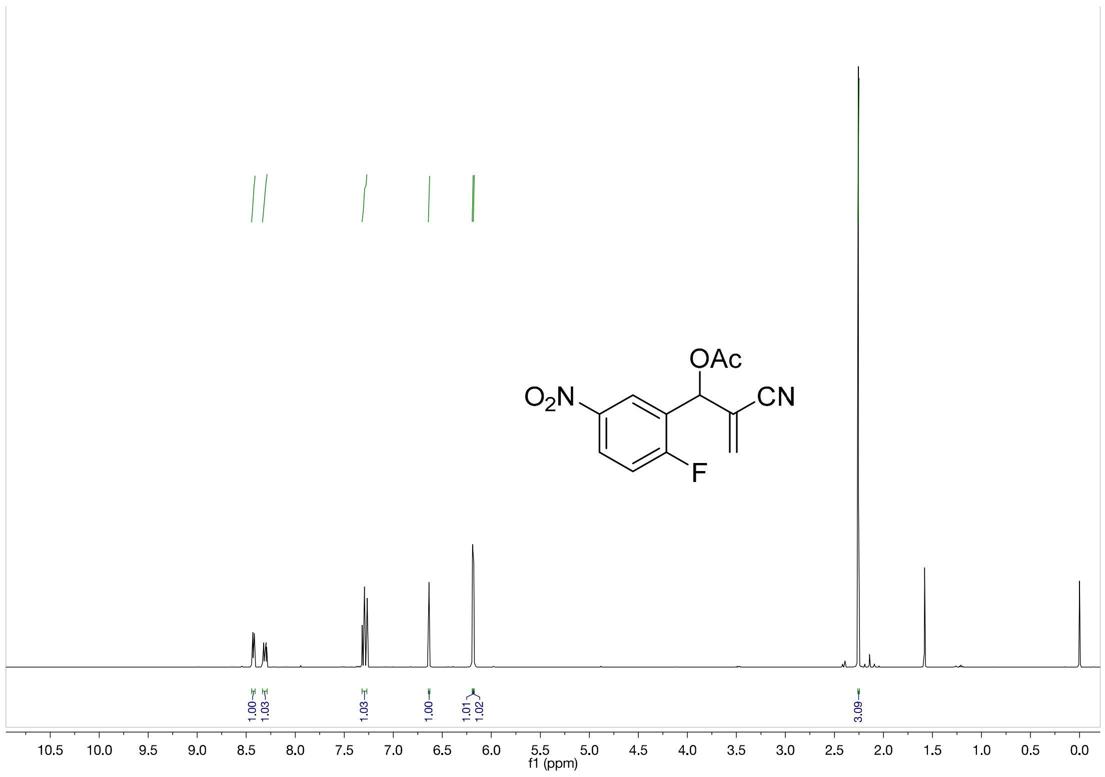

^{13}C NMR for (±)-2-cyano-1-(2-fluoro-5-nitrophenyl)allyl acetate (19)

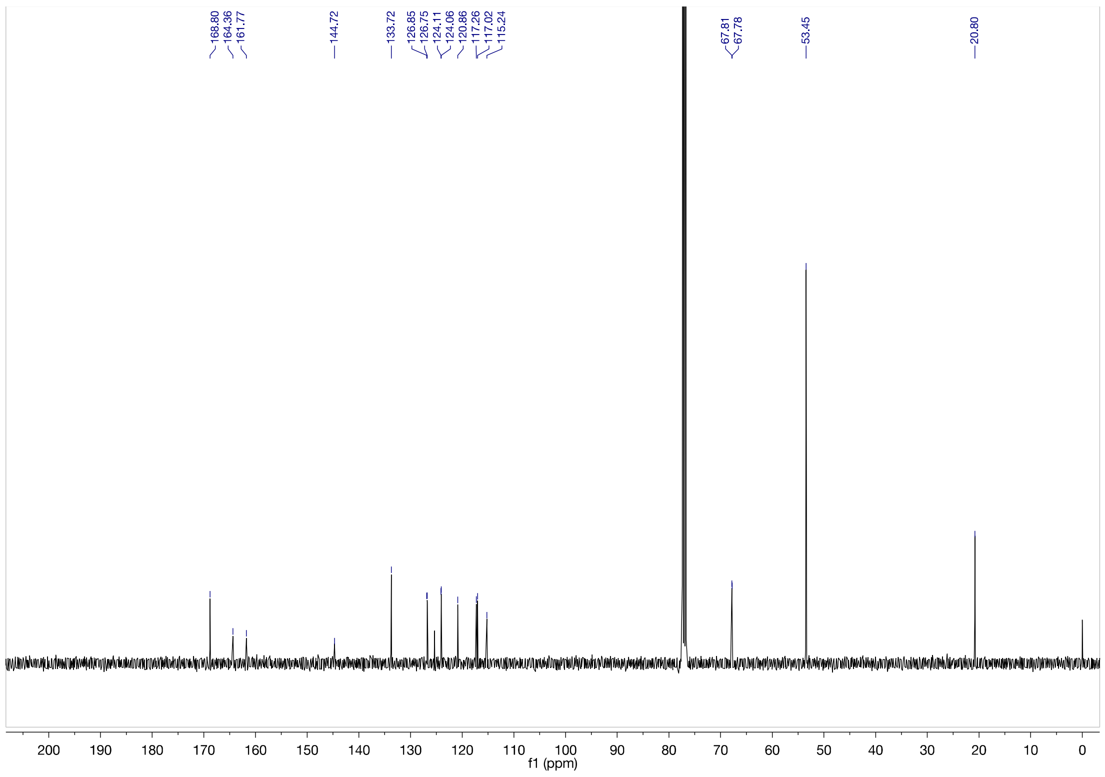

^{1}H NMR for (±)-2-cyano-1-(5-cyano-2-fluorophenyl)allyl acetate (20)

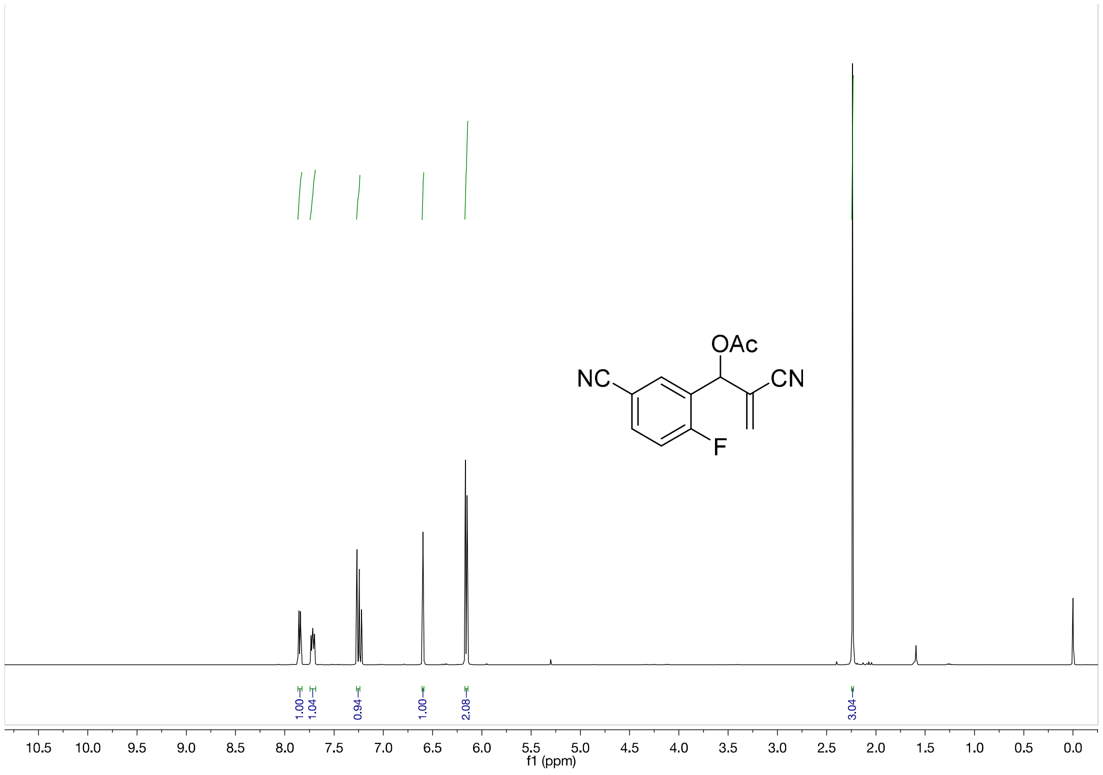

^{13}C NMR for (±)-2-cyano-1-(5-cyano-2-fluorophenyl)allyl acetate (20)

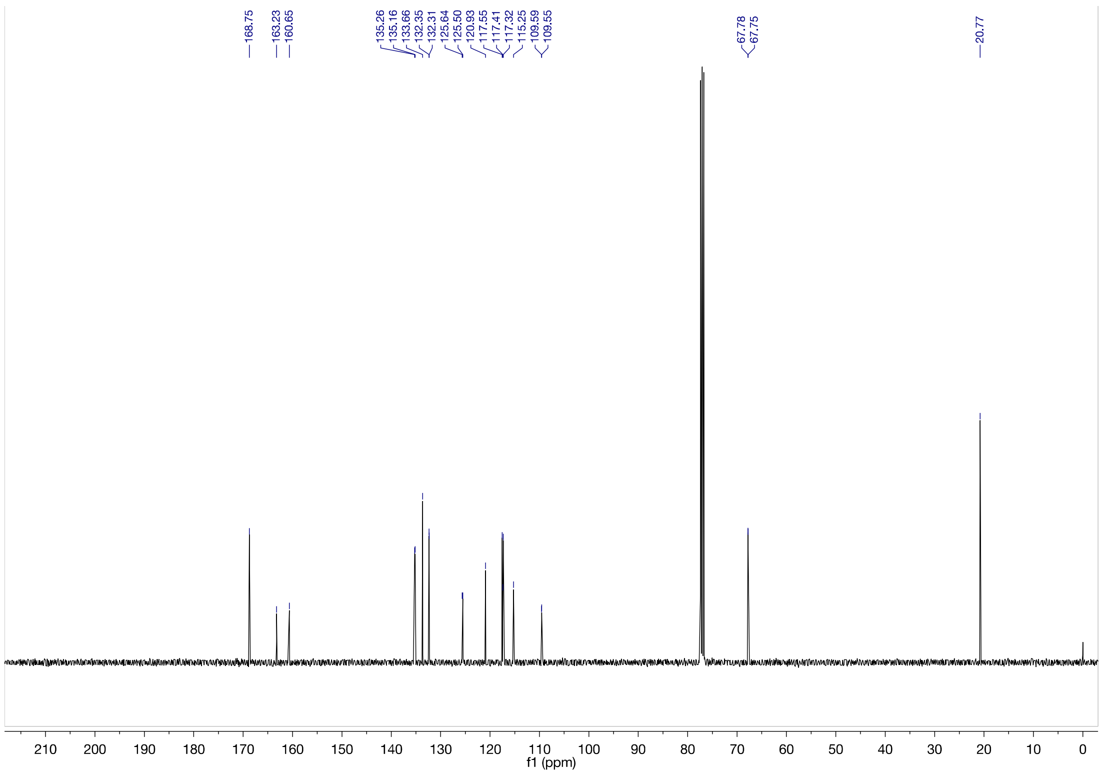

¹H NMR for (±)-2-cyano-1-(2-fluoro-5-(trifluoromethyl)phenyl)allyl acetate (21)

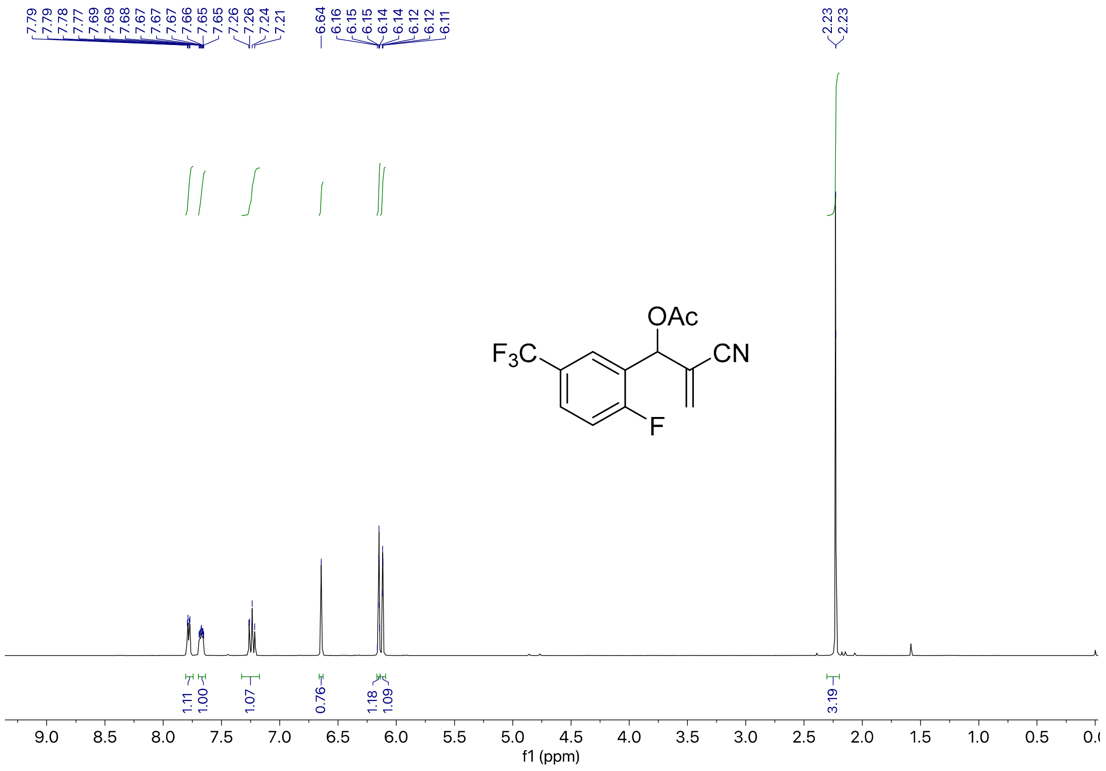

¹³C NMR for (±)-2-cyano-1-(2-fluoro-5-(trifluoromethyl)phenyl)allyl acetate (21)

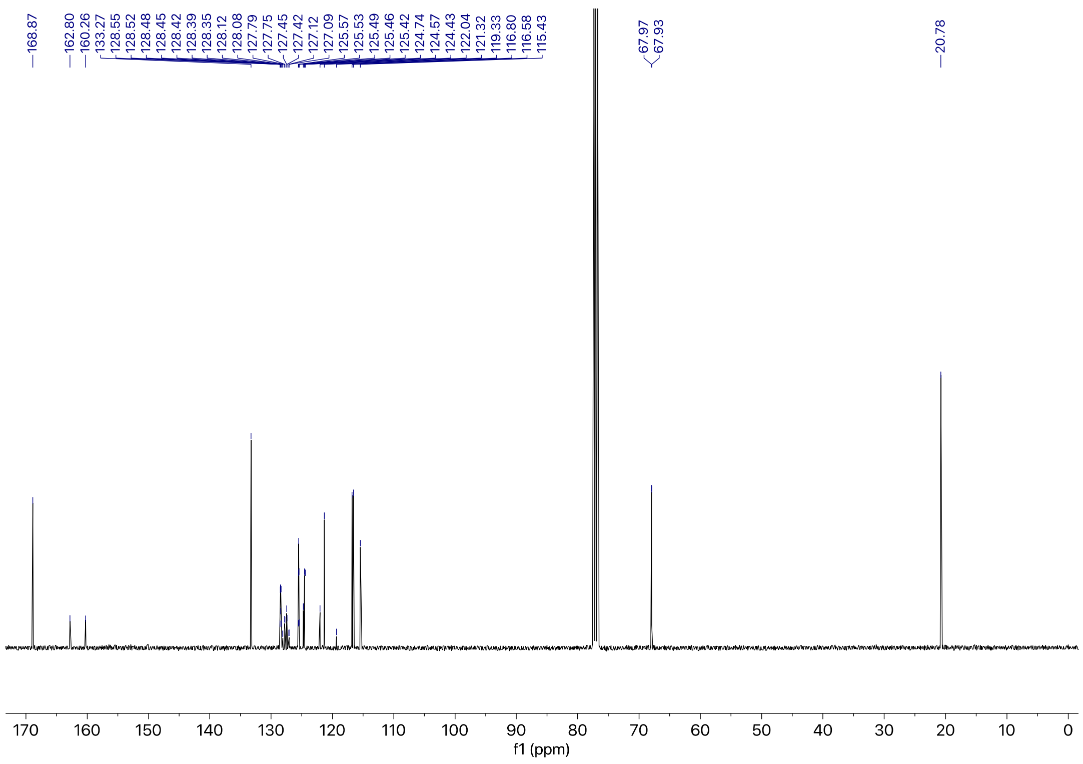

¹H NMR for (±)-1-(5-chloro-2-fluorophenyl)-2-cyanoallyl acetate (22)

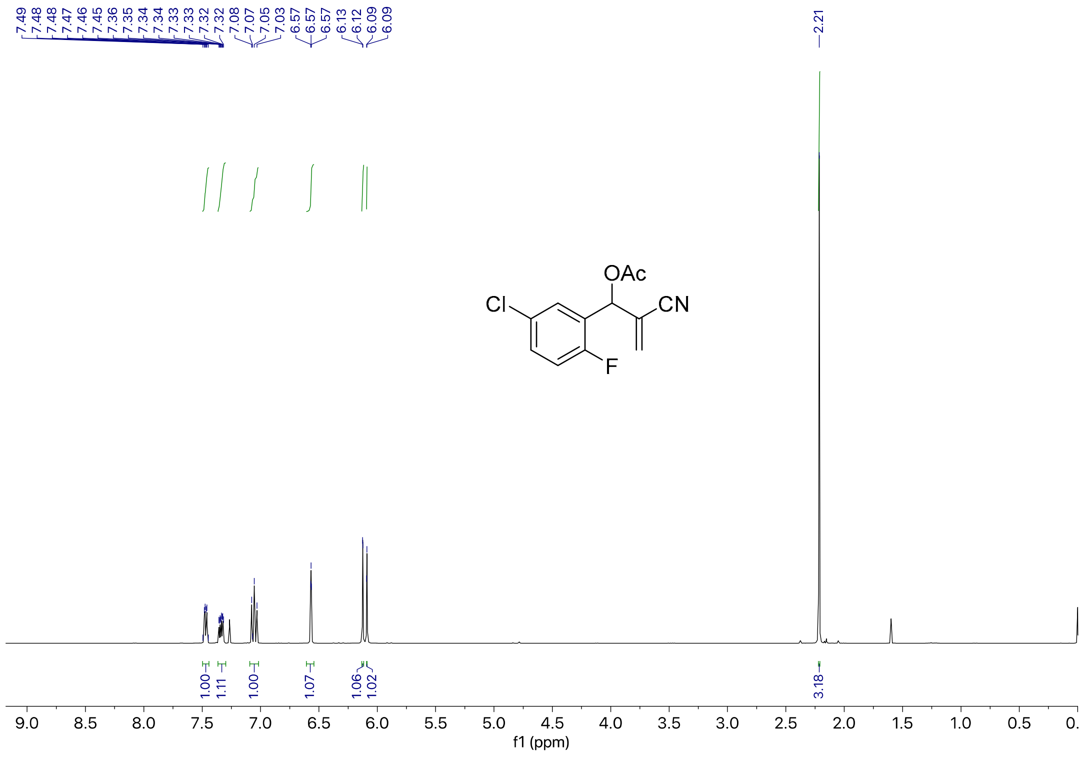

¹³C NMR for (±)-1-(5-chloro-2-fluorophenyl)-2-cyanoallyl acetate (22)

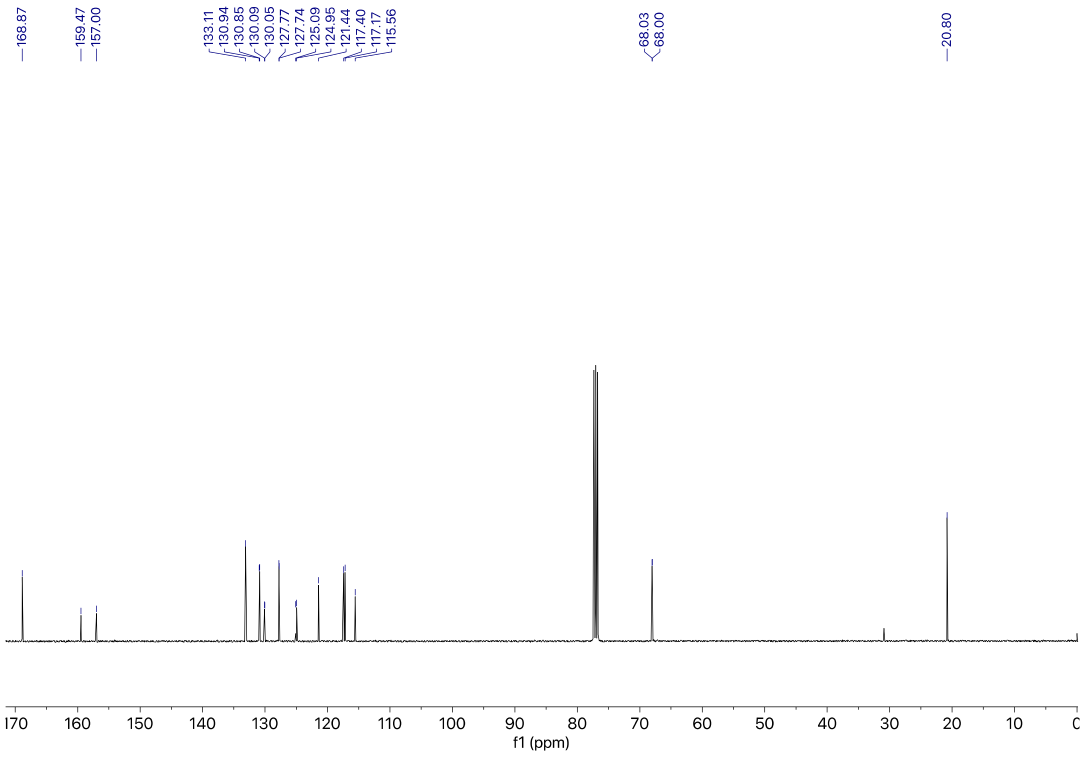

¹H NMR for (±)-1-(2-fluoro-5-nitrophenyl)-2-methylene-3-oxobutyl acetate (23)

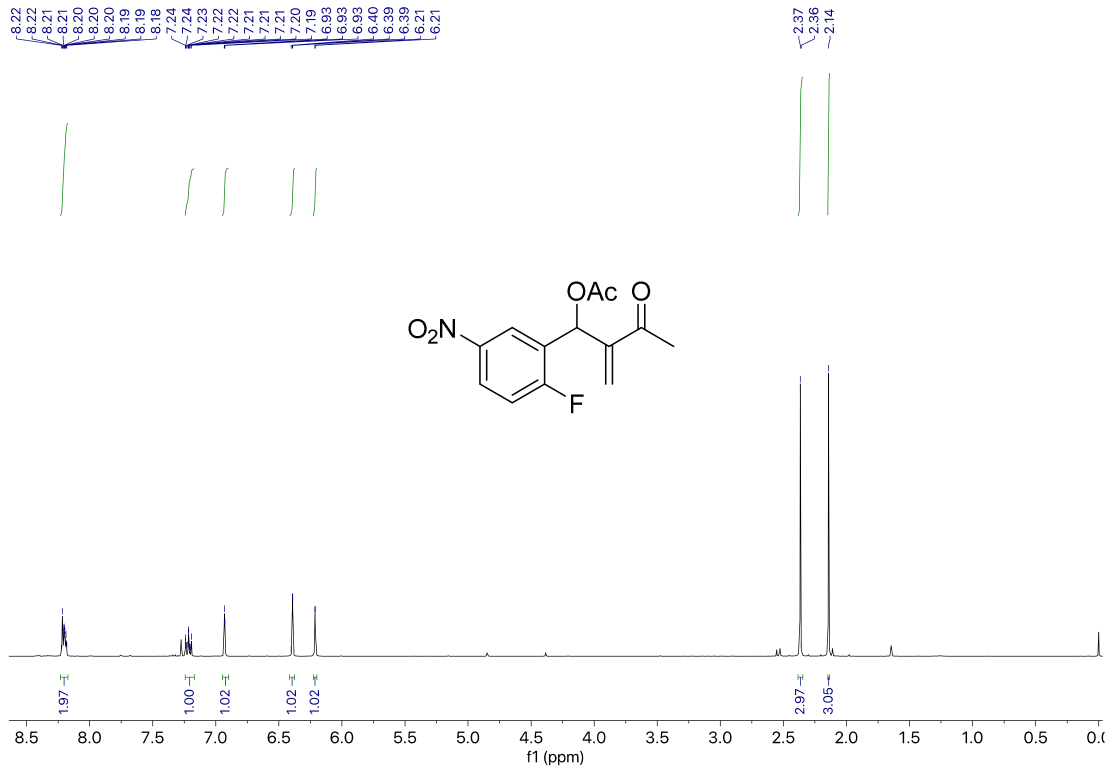

¹³C NMR or (±)-1-(2-fluoro-5-nitrophenyl)-2-methylene-3-oxobutyl acetate (23)

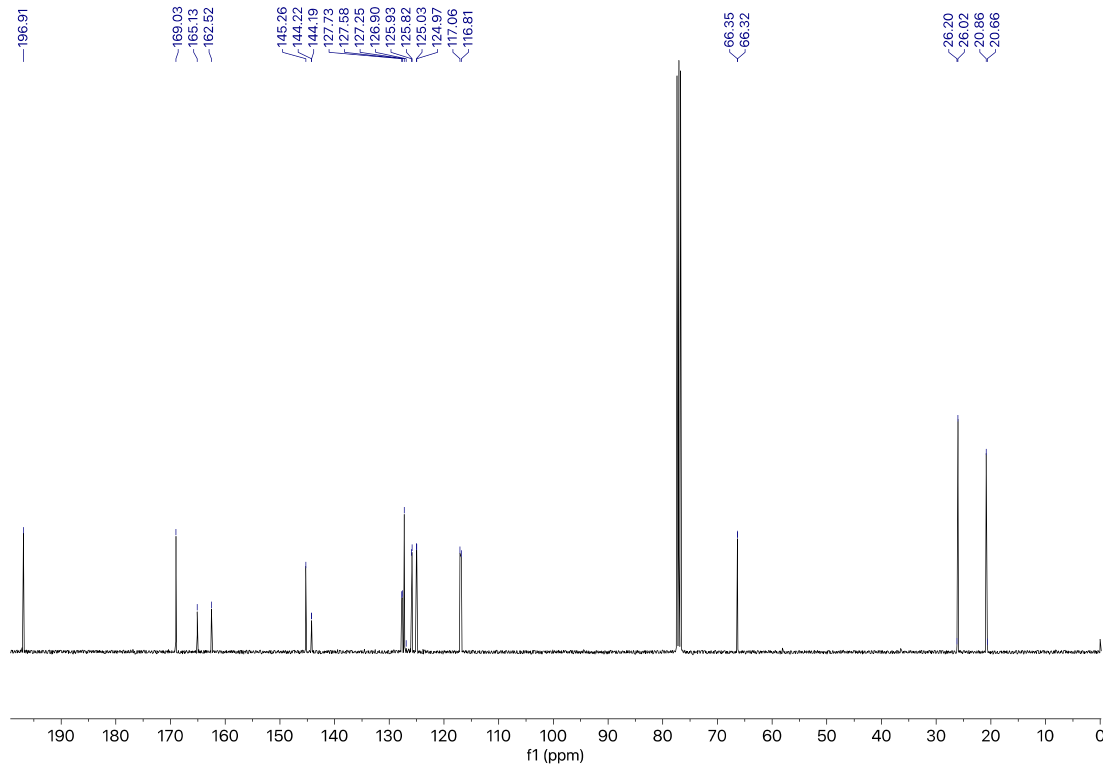

¹H NMR for (±)-1-(5-cyano-2-fluorophenyl)-2-methylene-3-oxobutyl acetate (24)

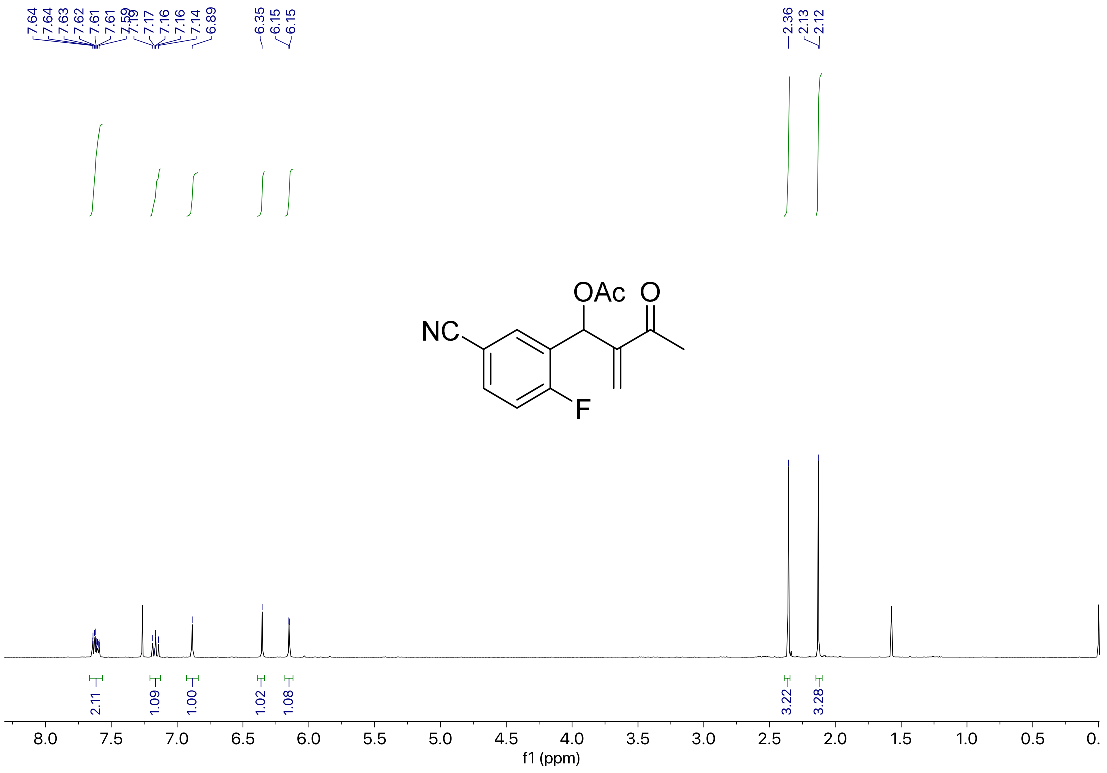

¹³C NMR for (±)-1-(5-cyano-2-fluorophenyl)-2-methylene-3-oxobutyl acetate (24)

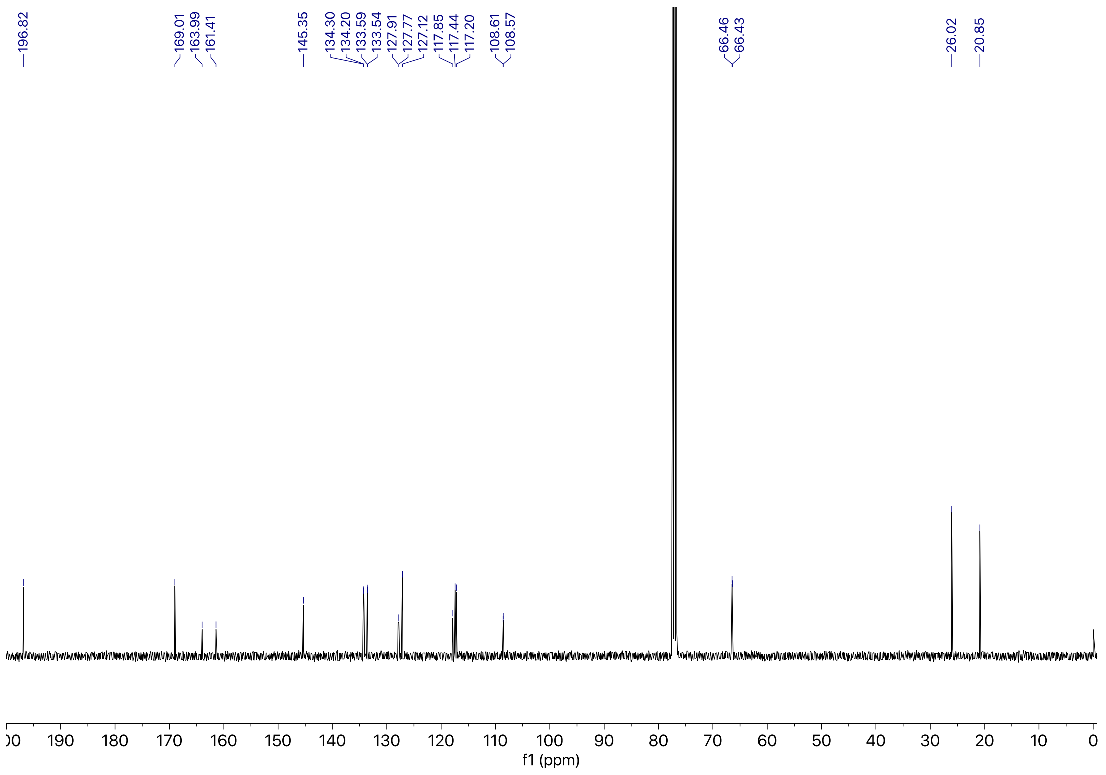

^{1}H NMR for (±)-1-(2-fluoro-5-(trifluoromethyl) phenyl)-2-methylene-3-oxobutyl acetate (25)

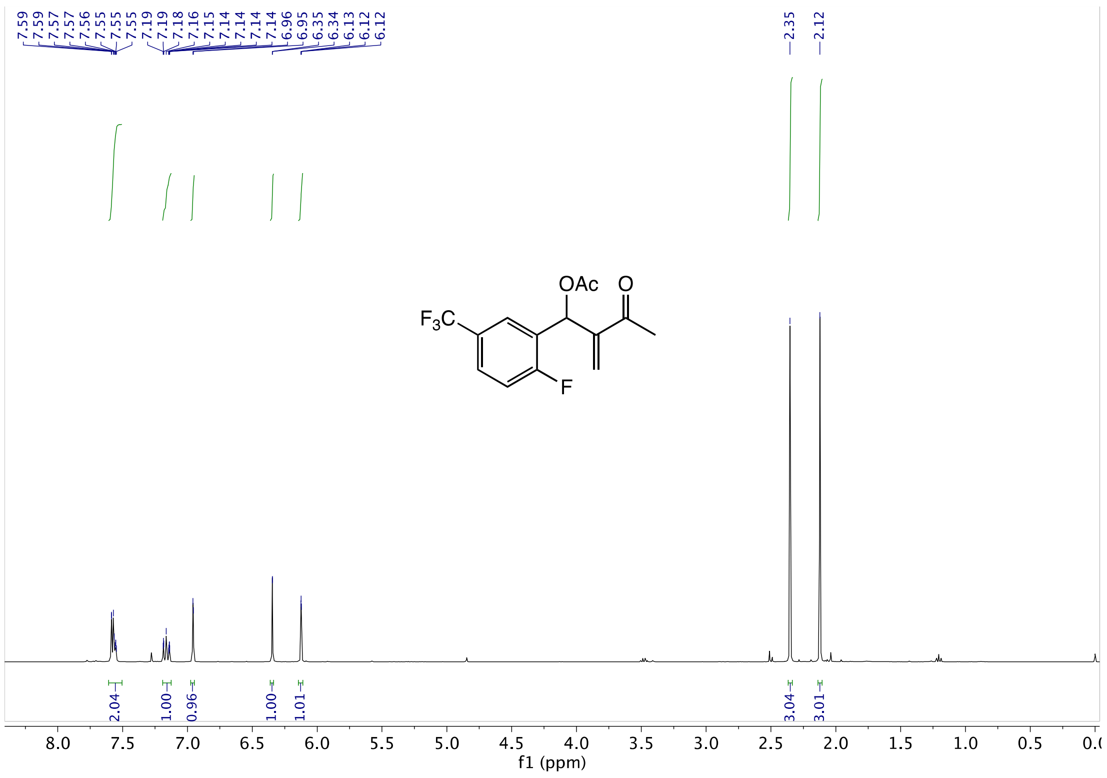

^{13}C NMR for (±)-1-(2-fluoro-5-(trifluoromethyl) phenyl)-2-methylene-3-oxobutyl acetate (25)

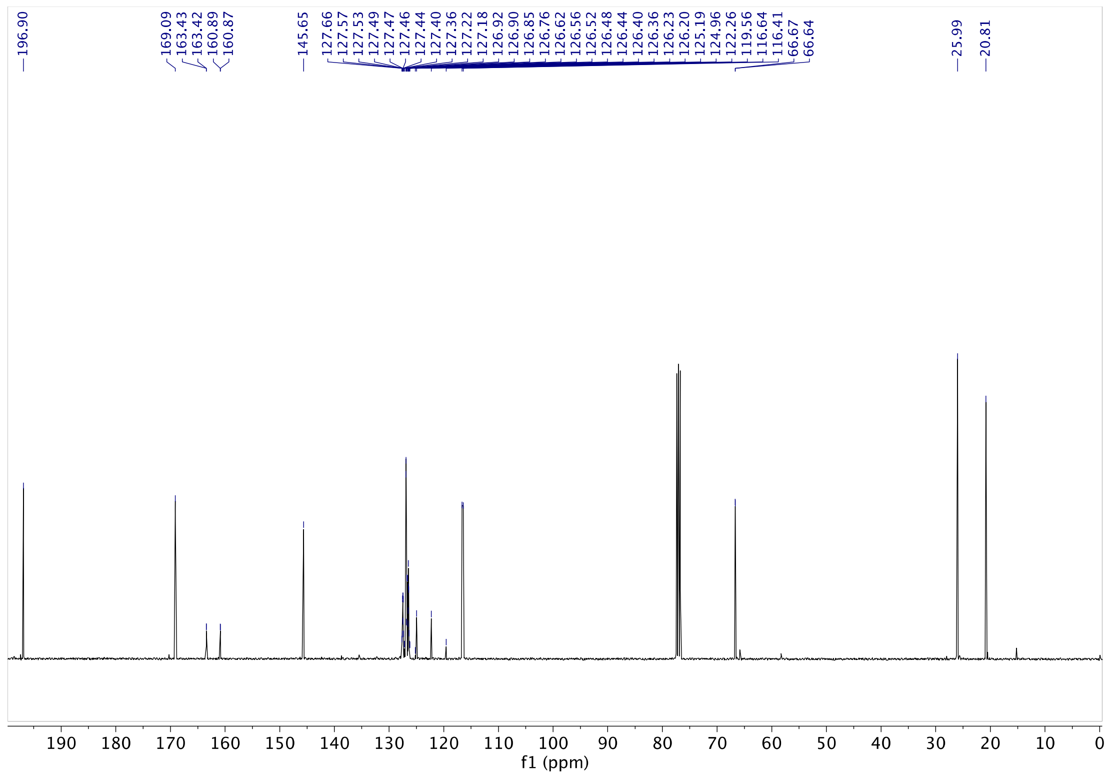

^{1}H NMR for (±)-2-methylene-3-oxo-1-(2,4,5-trifluorophenyl)butyl acetate (26)

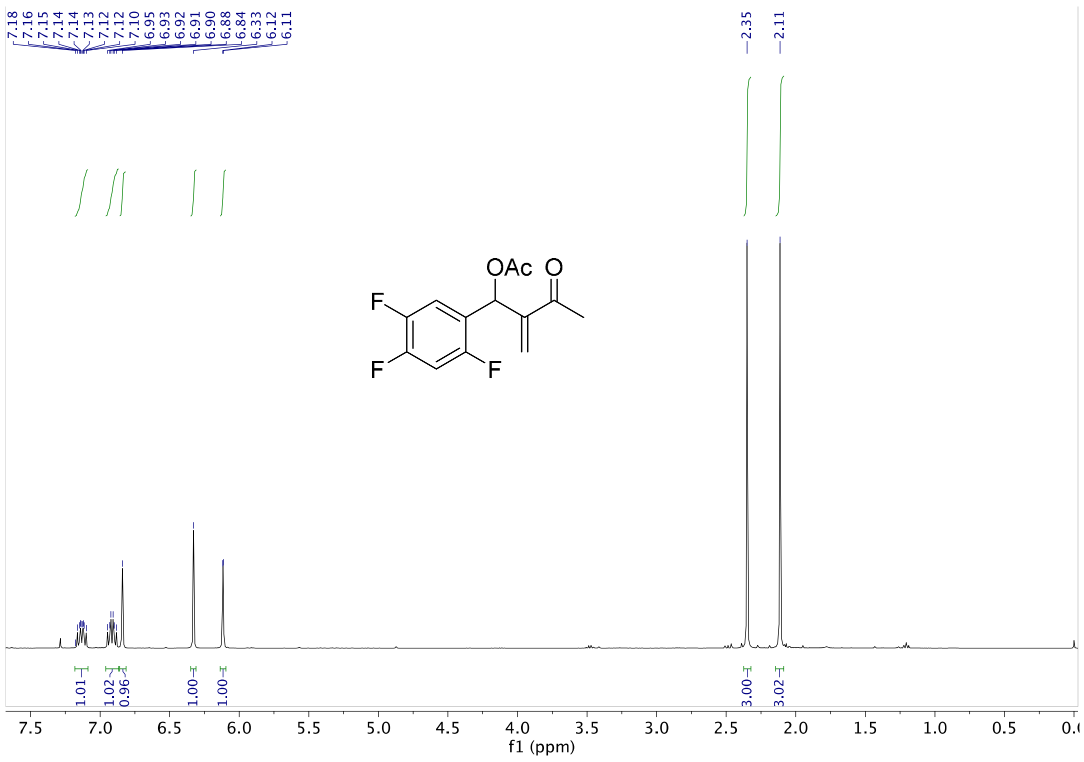

^{13}C NMR for (±)-2-methylene-3-oxo-1-(2,4,5-trifluorophenyl)butyl acetate (26)

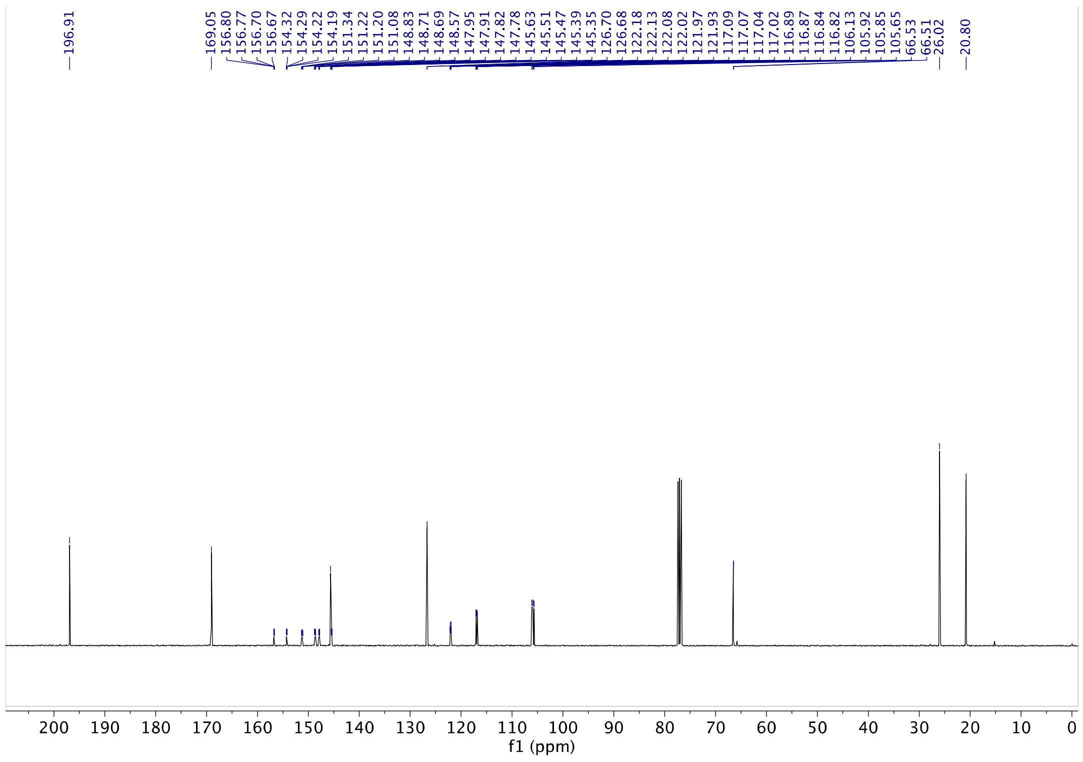

¹H NMR for (±)-1-(2-fluoropyridin-3-yl)-2-methylene-3-oxobutyl acetate (27)

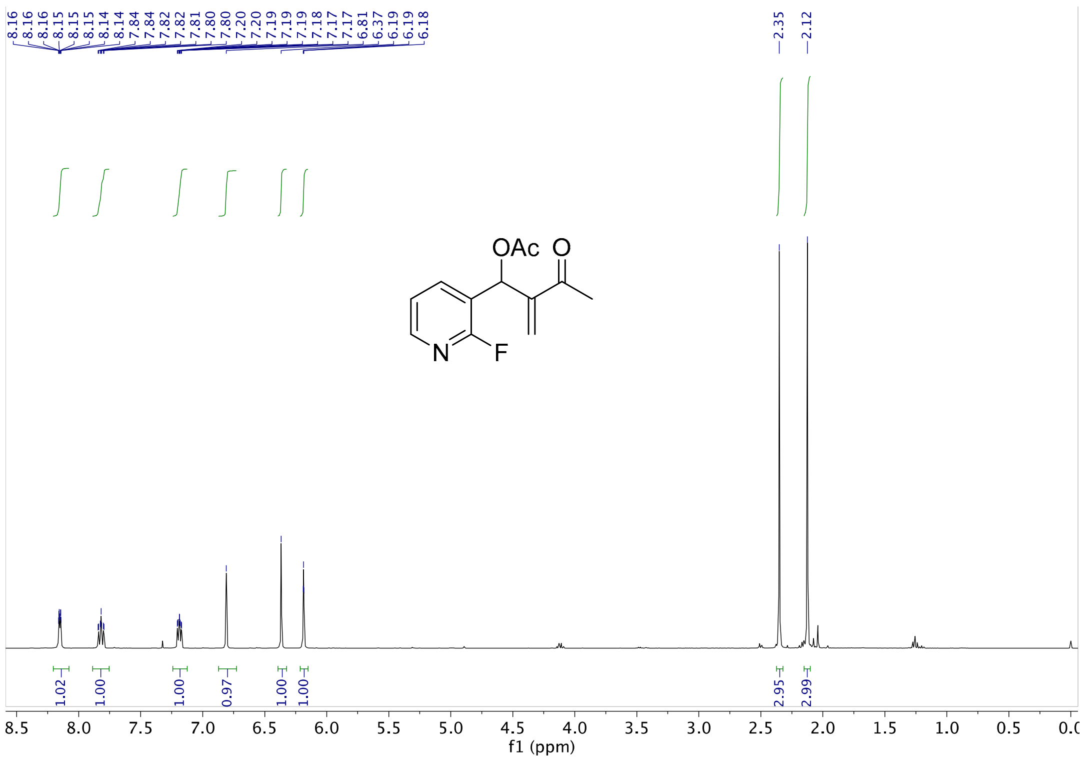

¹³C NMR for (±)-1-(2-fluoropyridin-3-yl)-2-methylene-3-oxobutyl acetate (27)

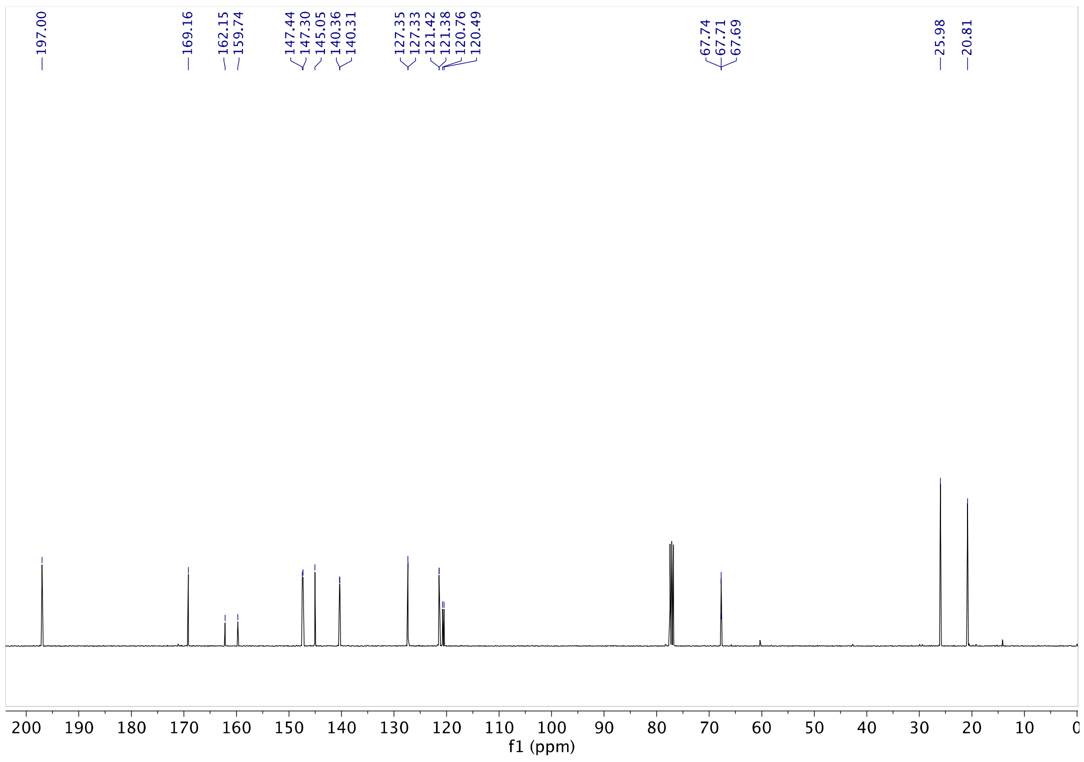

^{1}H NMR for ethyl 1-(methylsulfonyl)-6-nitro-1,2-dihydroquinoline-3-carboxylate (28-Ms)

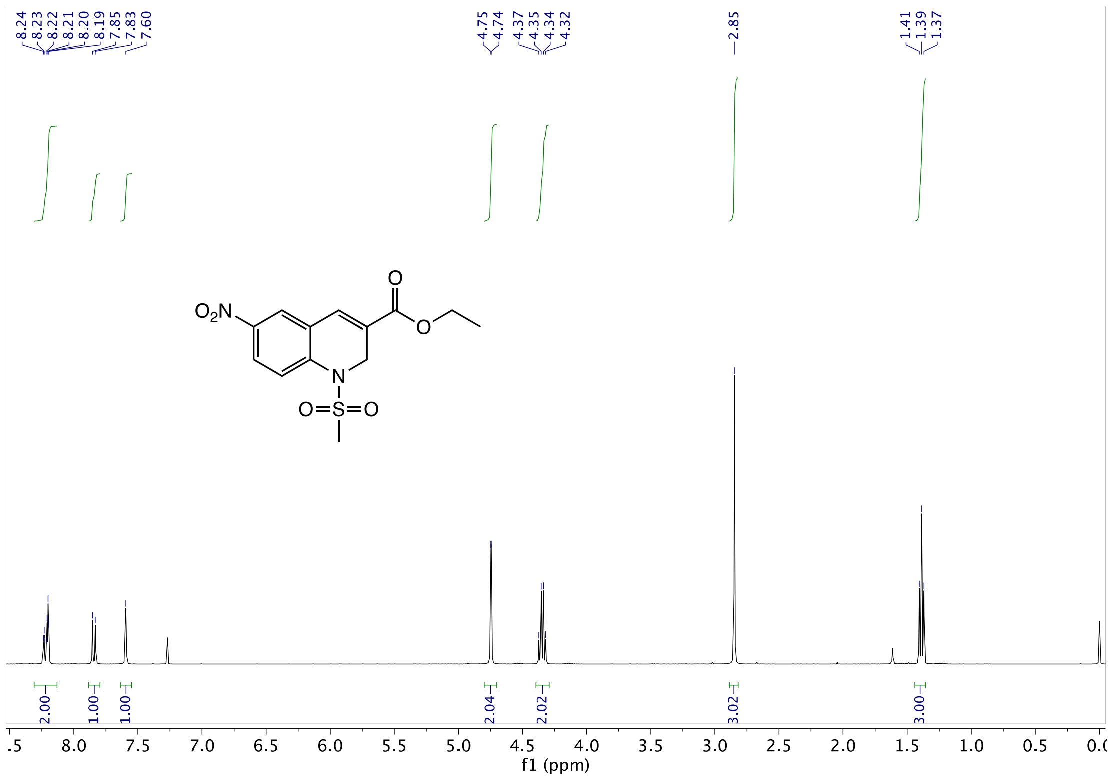

^{13}C NMR for ethyl 1-(methylsulfonyl)-6-nitro-1,2-dihydroquinoline-3-carboxylate (28-Ms)

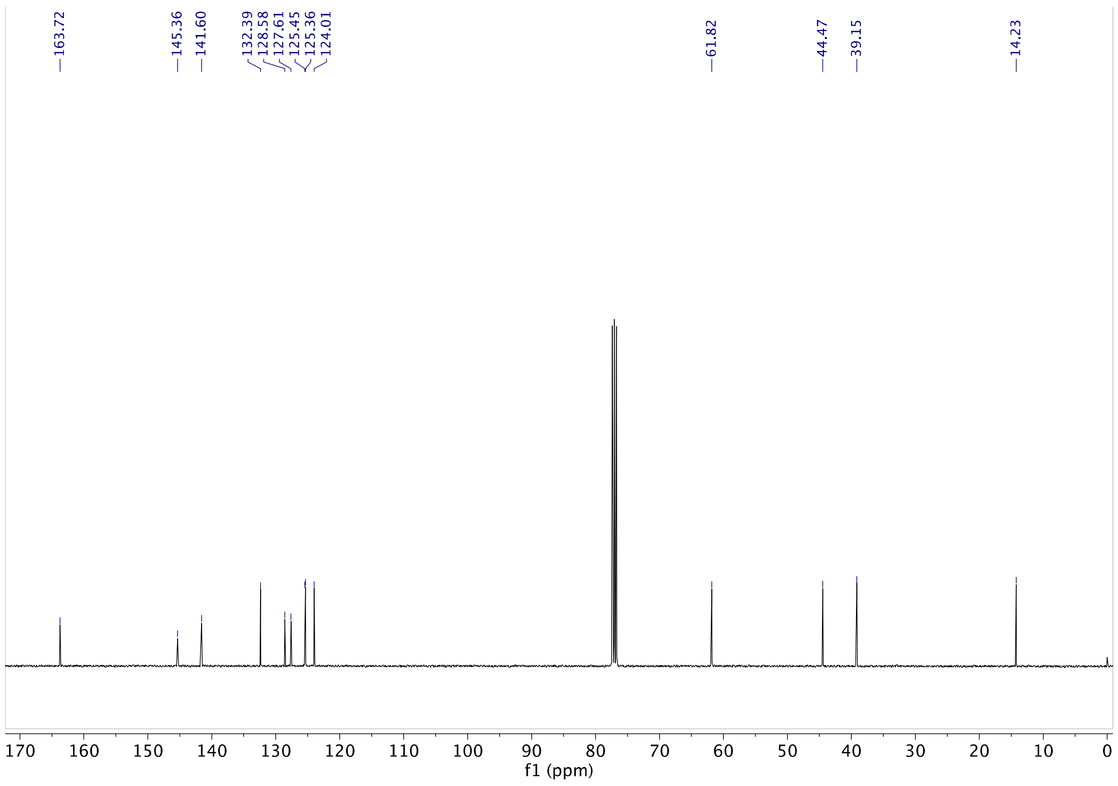

^{1}H NMR for ethyl 6-nitro-1-tosyl-1,2-dihydroquinoline-3-carboxylate (28-Ts)

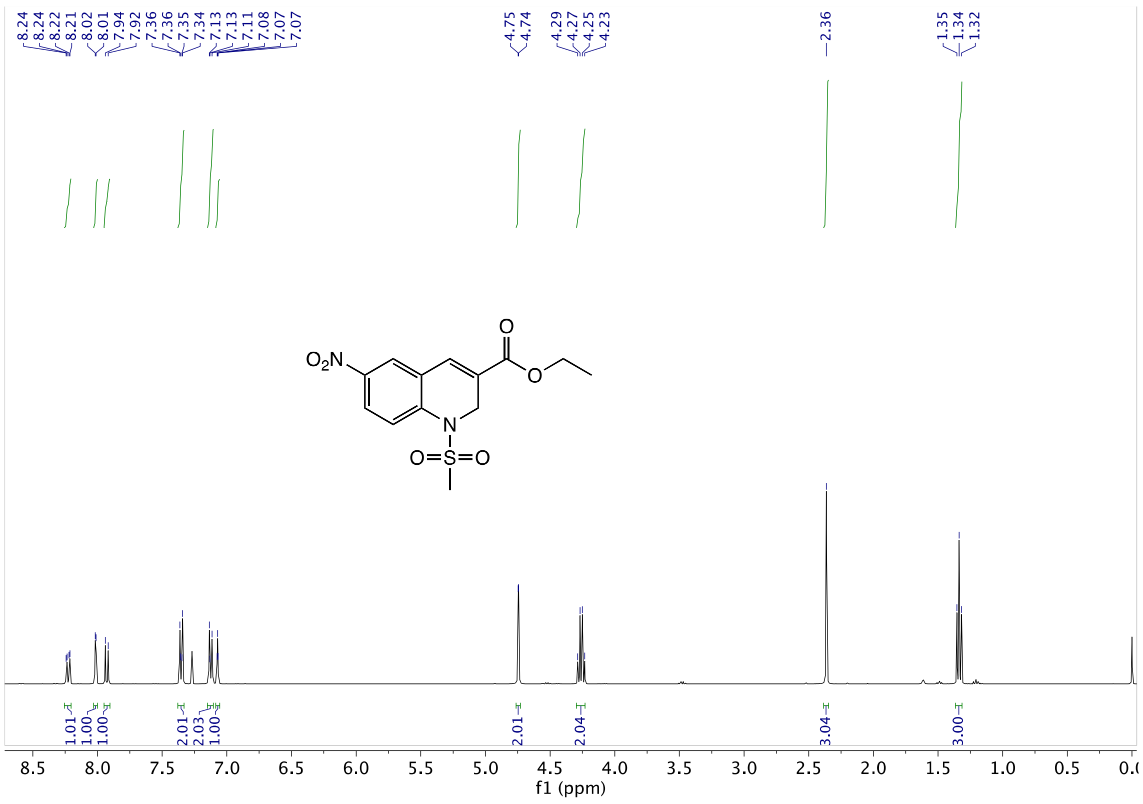

^{13}C NMR for ethyl 6-nitro-1-tosyl-1,2-dihydroquinoline-3-carboxylate (28-Ts)

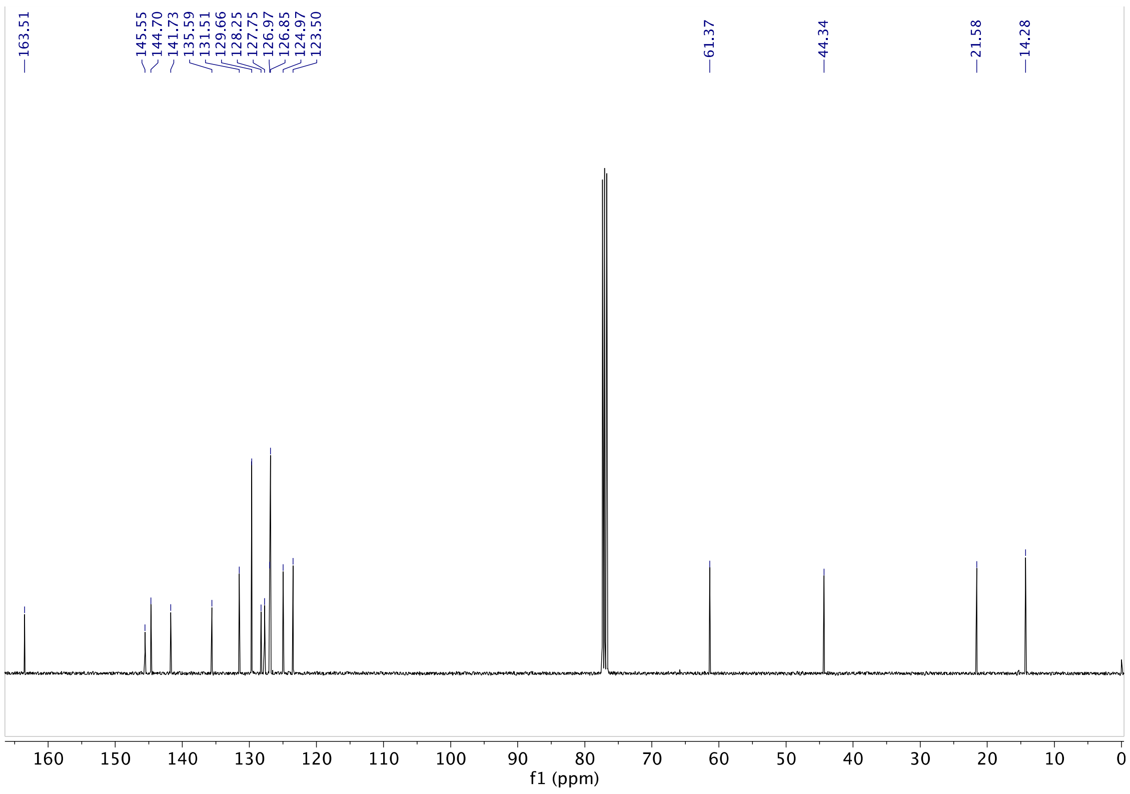

^{1}H NMR for ethyl 6-cyano-1-(methylsulfonyl)-1,2-dihydroquinoline-3-carboxylate (29)

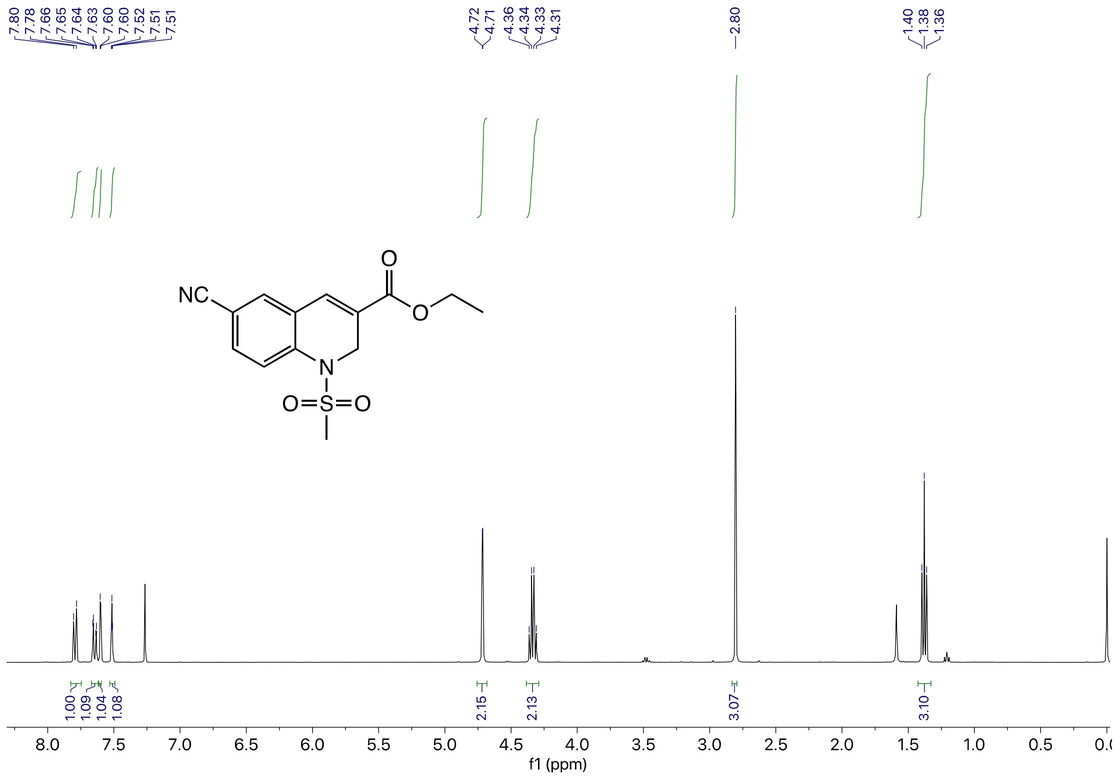

^{13}C NMR for ethyl 6-cyano-1-(methylsulfonyl)-1,2-dihydroquinoline-3-carboxylate (29)

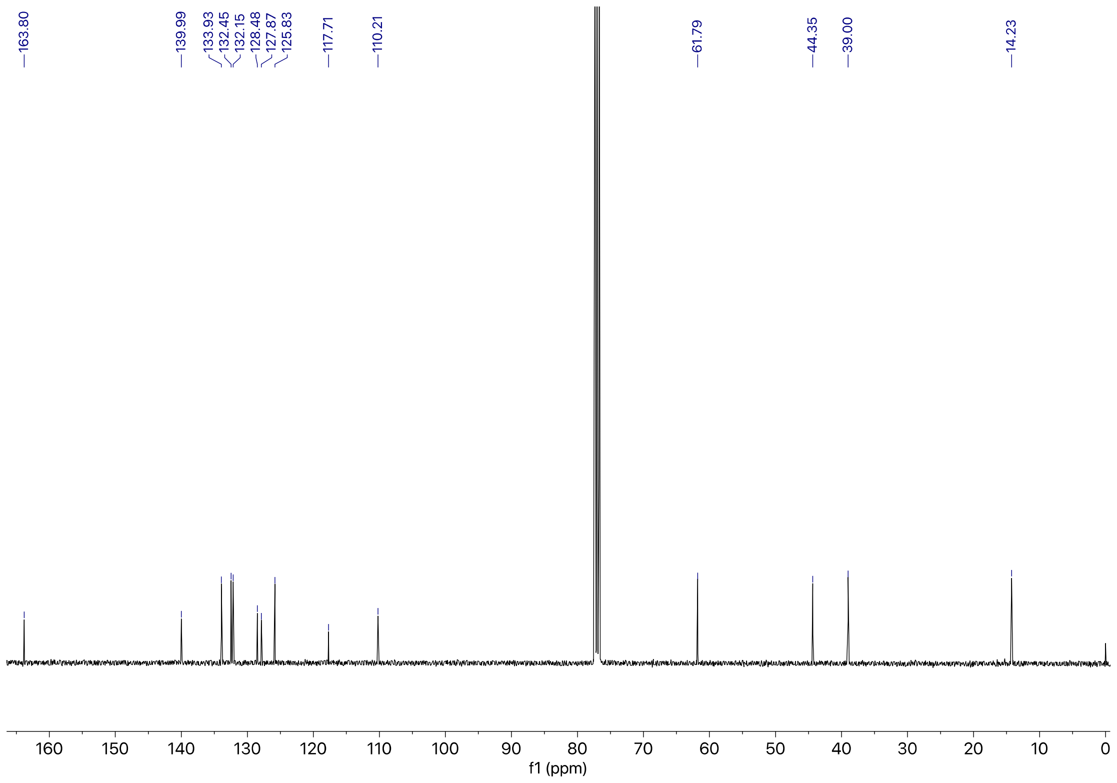

^{1}H NMR for ethyl 1-(methylsulfonyl)-6-(trifluoromethyl)-1,2-dihydroquinoline-3-carboxylate (30)

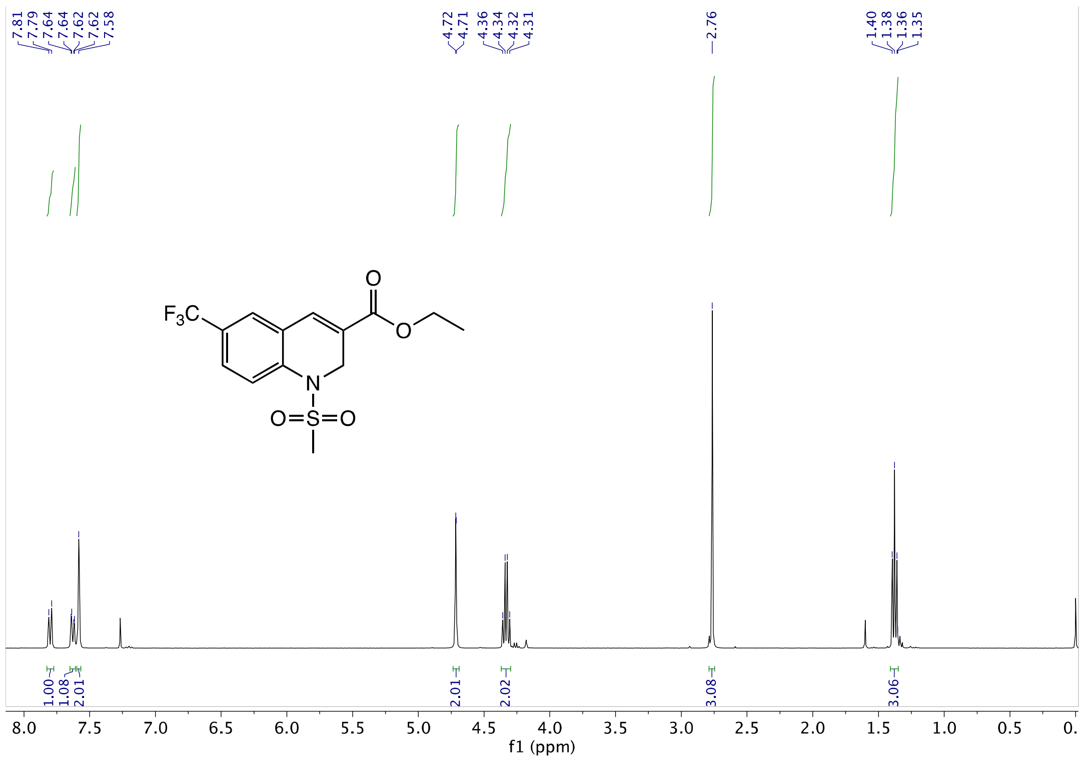

^{13}C NMR for ethyl 1-(methylsulfonyl)-6-(trifluoromethyl)-1,2-dihydroquinoline-3-carboxylate (30)

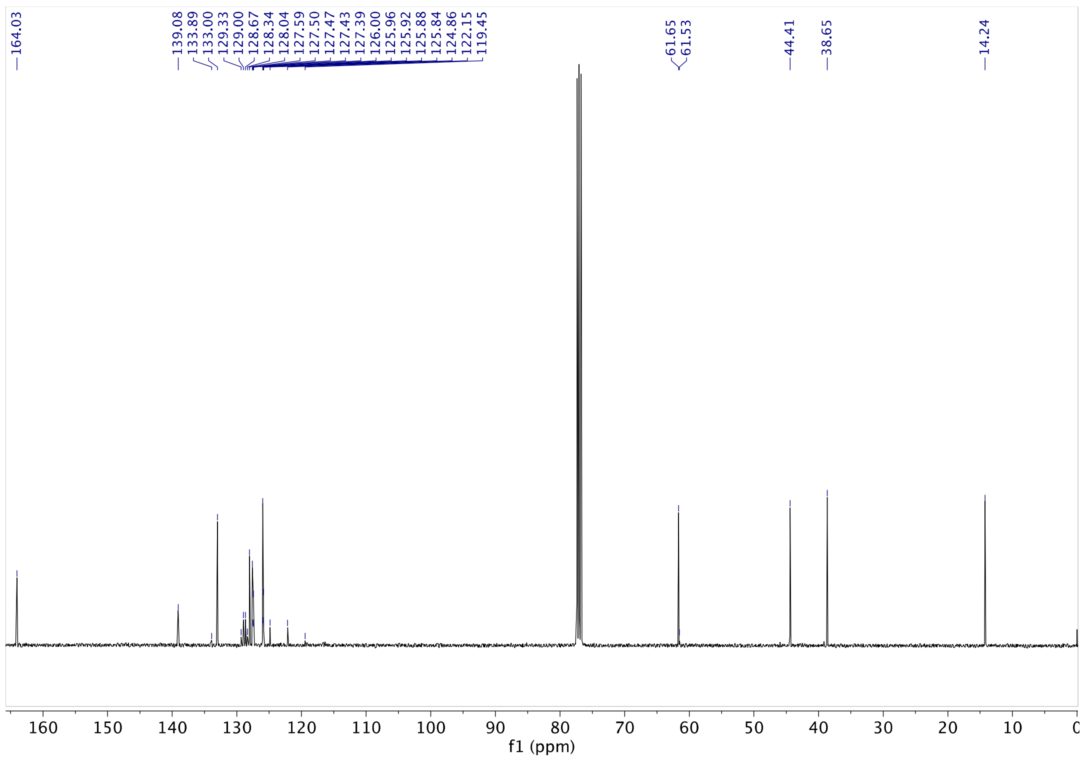

^{1}H NMR for ethyl 6-chloro-1-(methylsulfonyl)-1,2-dihydroquinoline-3-carboxylate (31)

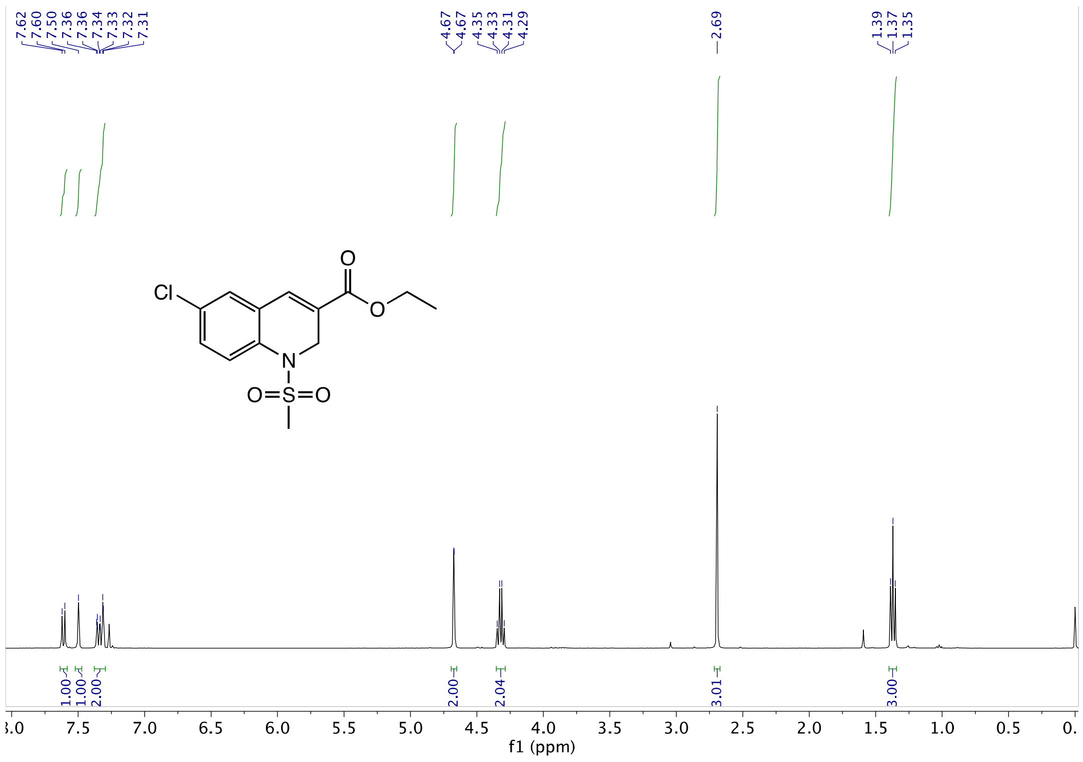

^{13}C NMR for ethyl 6-chloro-1-(methylsulfonyl)-1,2-dihydroquinoline-3-carboxylate (31)

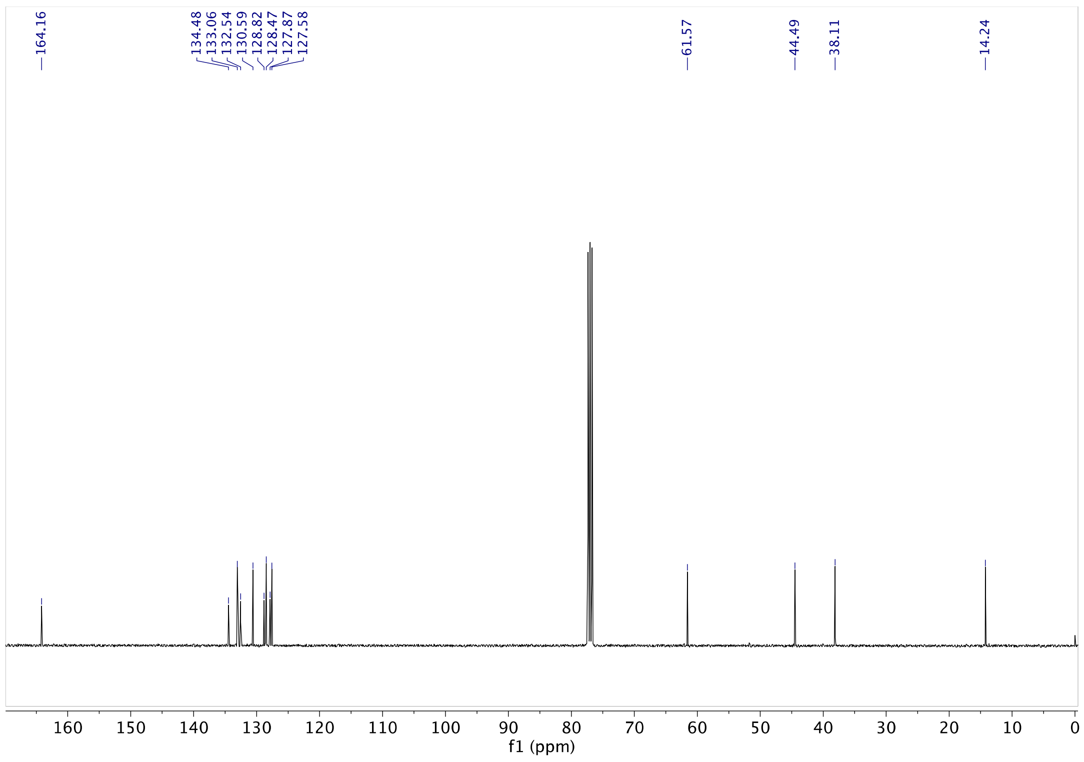

^{1}H NMR for ethyl 6,7-difluoro-1-(methylsulfonyl)-1,2-dihydroquinoline-3-carboxylate (32)

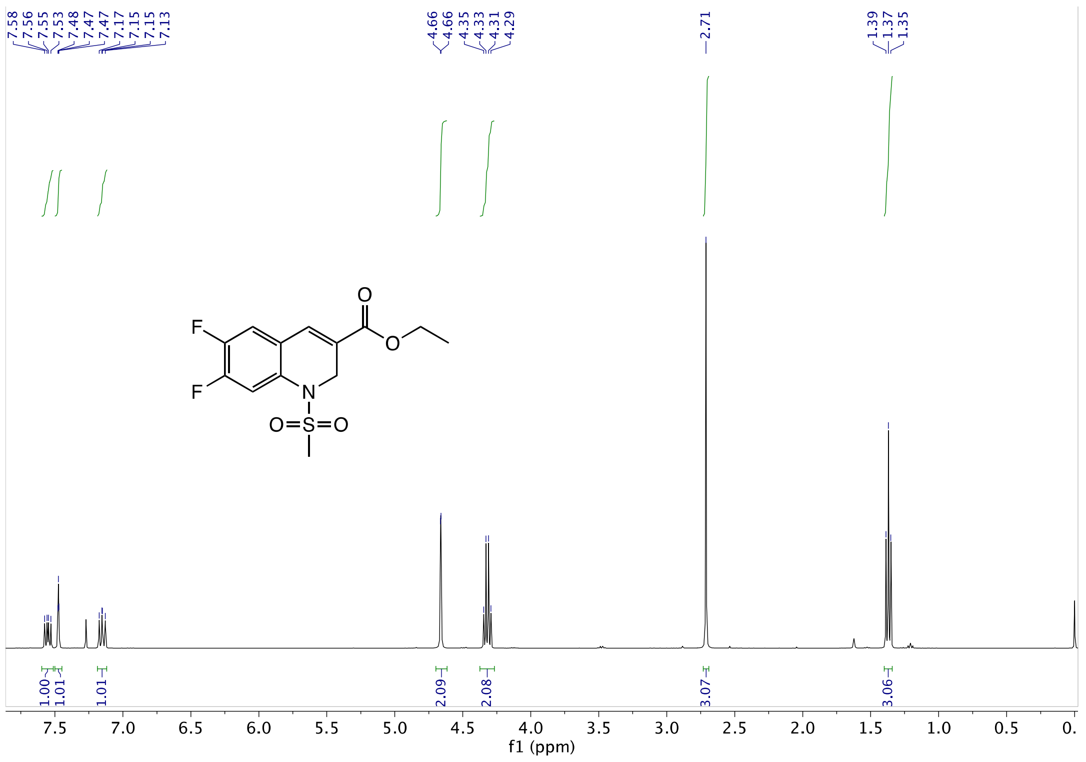

^{13}C NMR for ethyl 6,7-difluoro-1-(methylsulfonyl)-1, 2-dihydroquinoline-3-carboxylate (32)

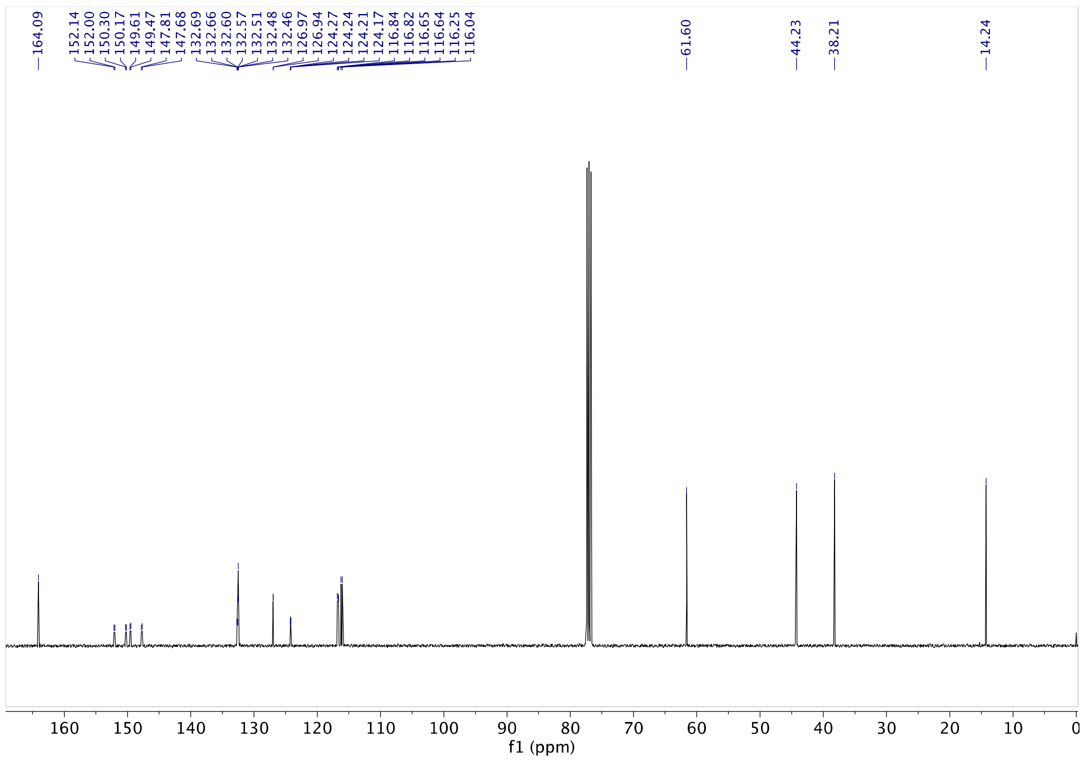

^{1}H NMR for ethyl 1-(methylsulfonyl)-1,2-dihydro-1,8-naphthyridine-3-carboxylate (33)

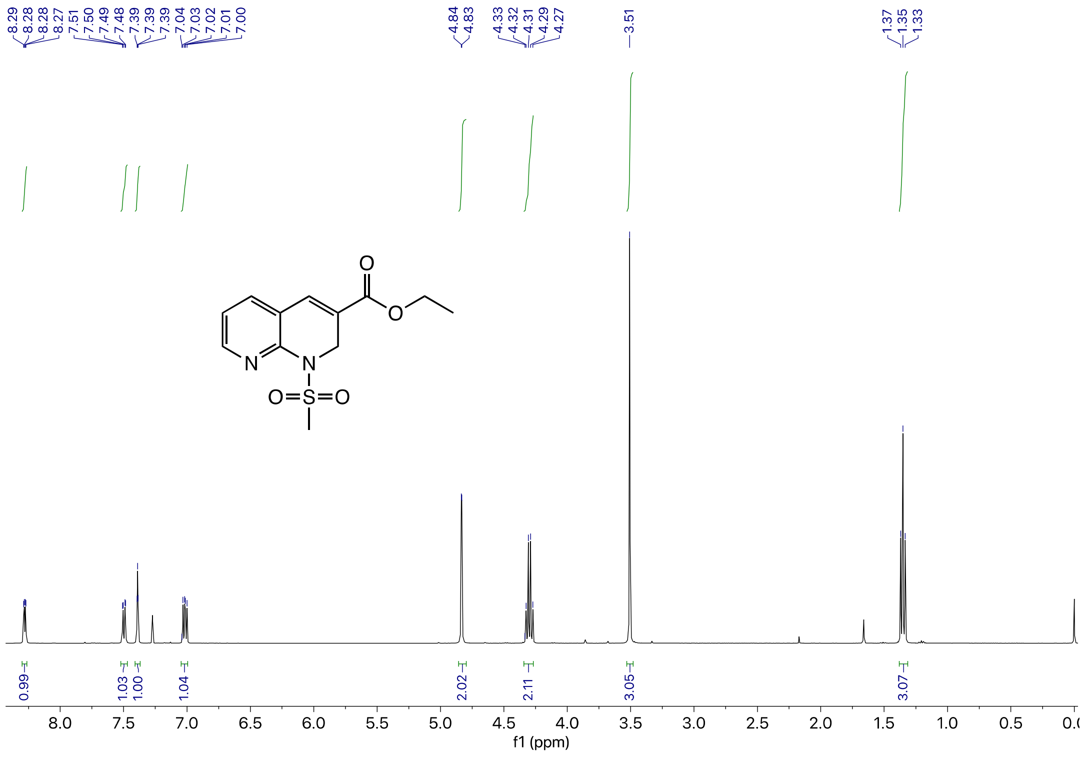

^{13}C NMR for ethyl 1-(methylsulfonyl)-1,2-dihydro-1,8-naphthyridine-3-carboxylate (33)

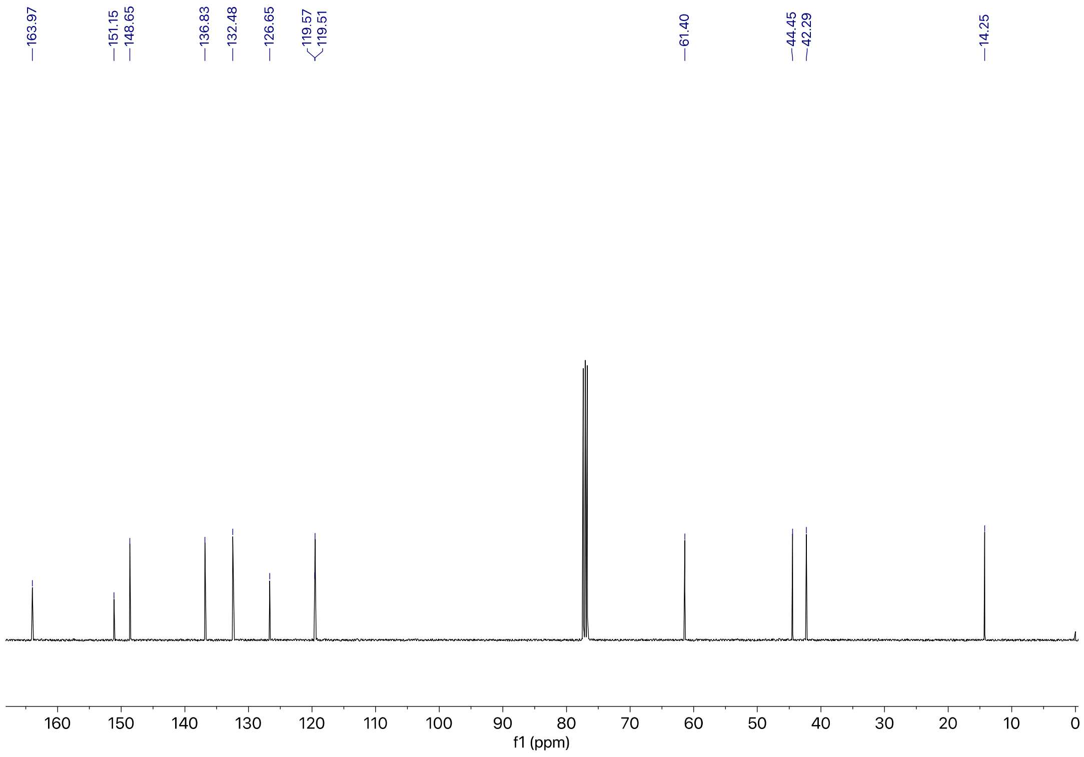

^{1}H NMR of 1-(methylsulfonyl)-6-nitro-1,2-dihydronaphthalene-3-carbonitrile (34)

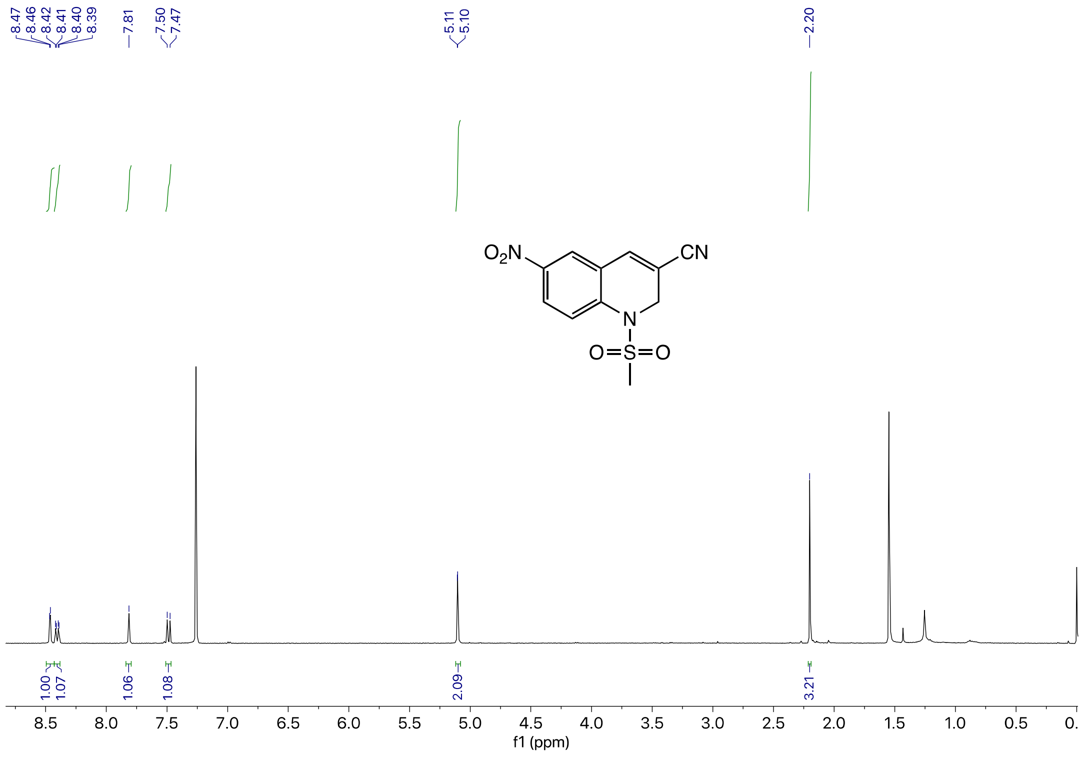

^{13}C NMR of 1-(methylsulfonyl)-6-nitro-1,2-dihydronaphthalene-3-carbonitrile (34)

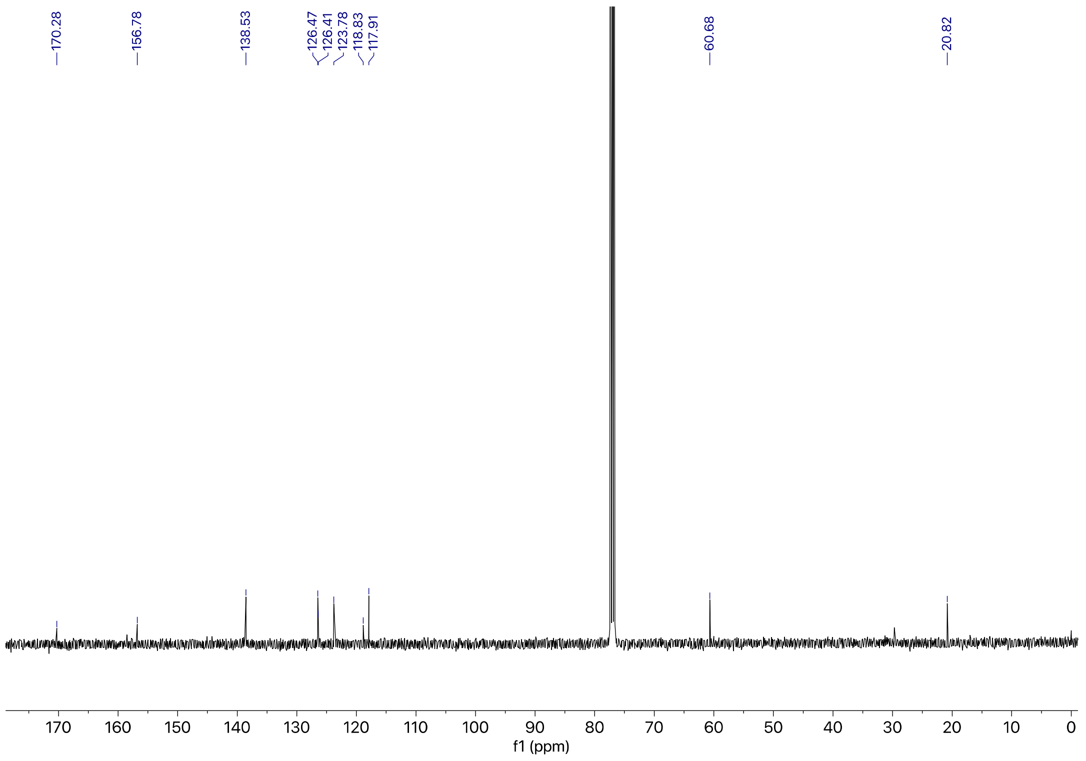

^{1}H NMR for 1-(1-(methylsulfonyl)-6-nitro-1,2-dihydroquinolin-3-yl)ethan-1-one (38)

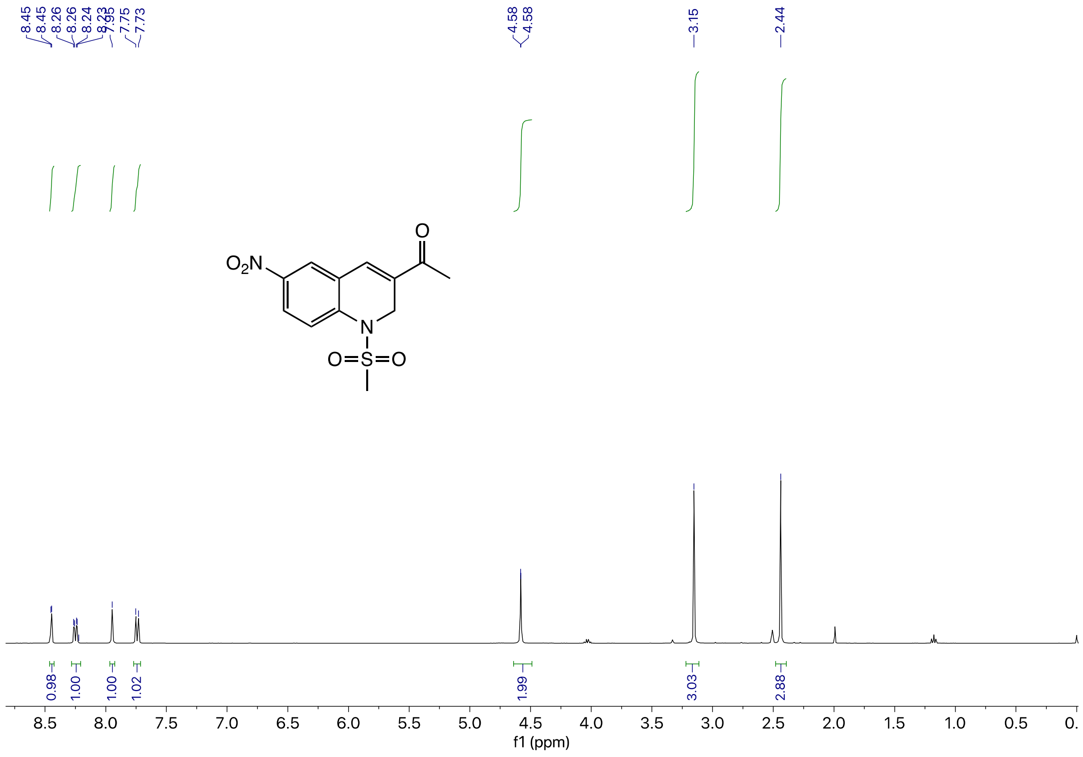

^{13}C NMR for 1-(1-(methylsulfonyl)-6-nitro-1,2-dihydroquinolin-3-yl)ethan-1-one (38)

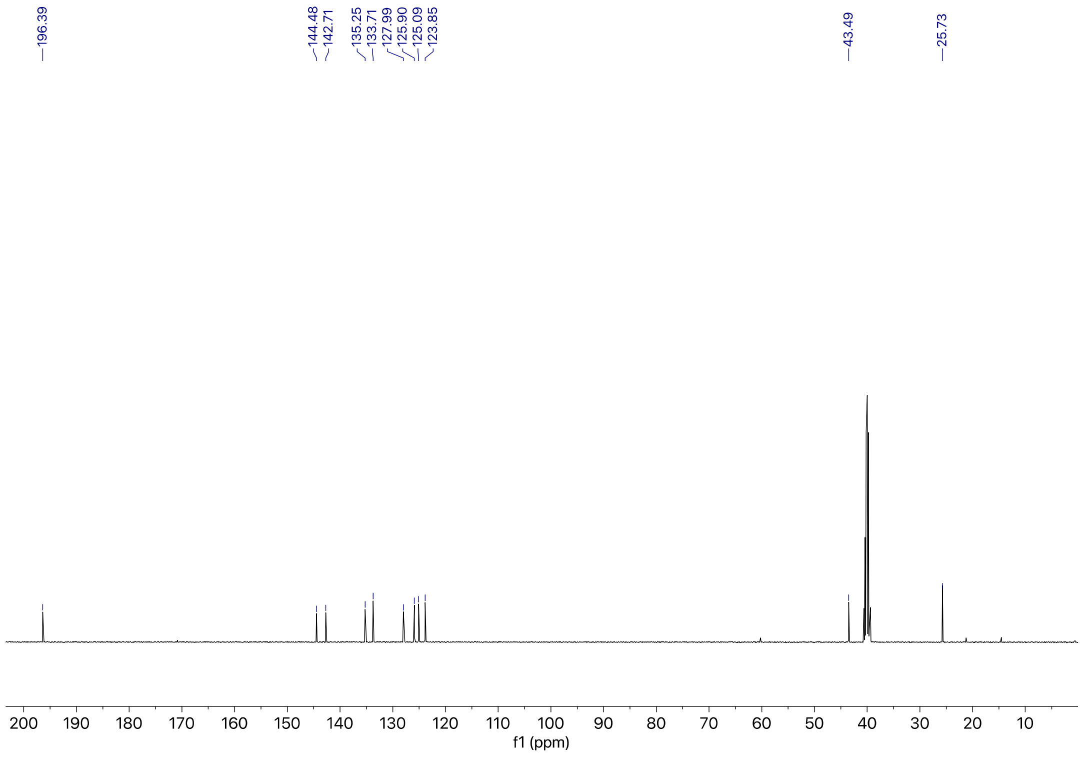

¹H NMR of 3-acetyl-1-(methylsulfonyl)-1,2-dihydroquinoline-6-carbonitrile (39)

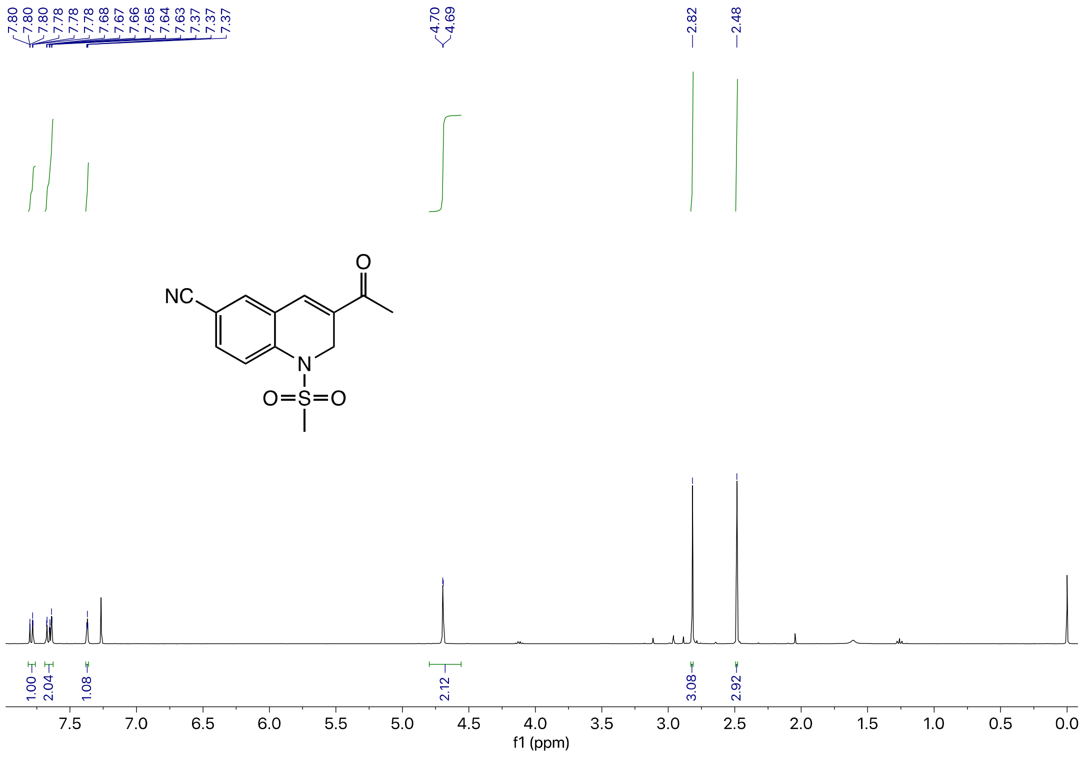

¹³C NMR of 3-acetyl-1-(methylsulfonyl)-1,2-dihydroquinoline-6-carbonitrile (39)

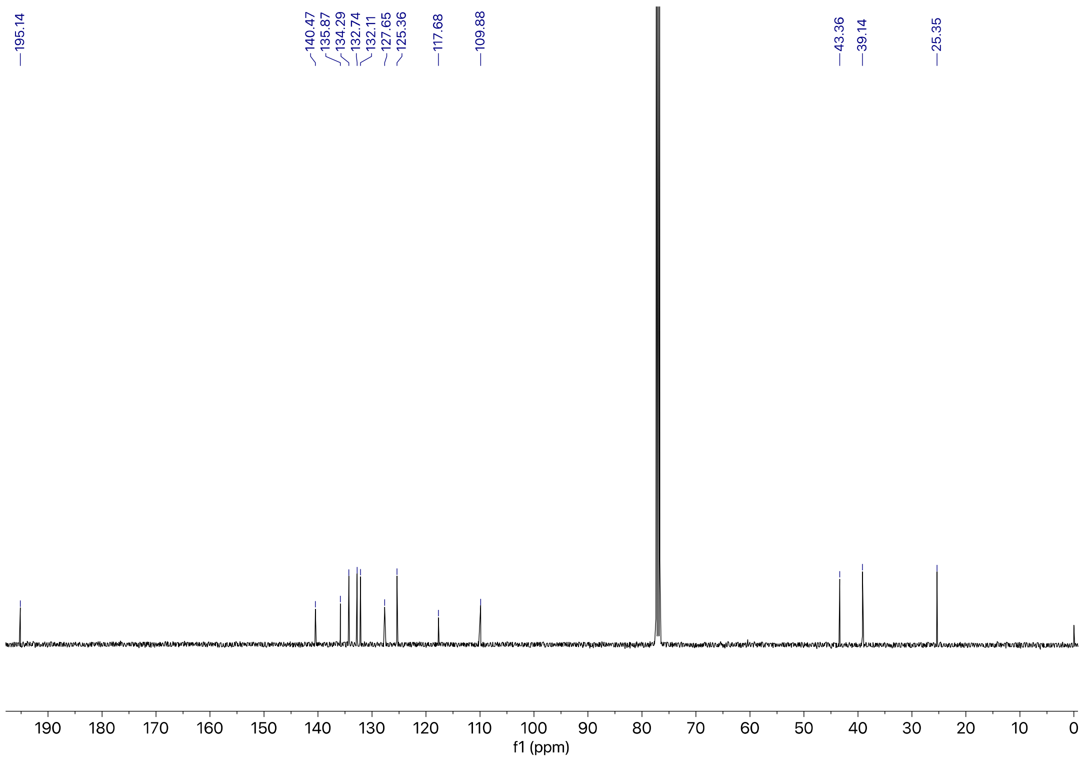

¹H NMR for 1-(1-(methylsulfonyl)-6-(trifluoromethyl)-1,2-dihydroquinolin-3-yl)ethan-1-one (40)

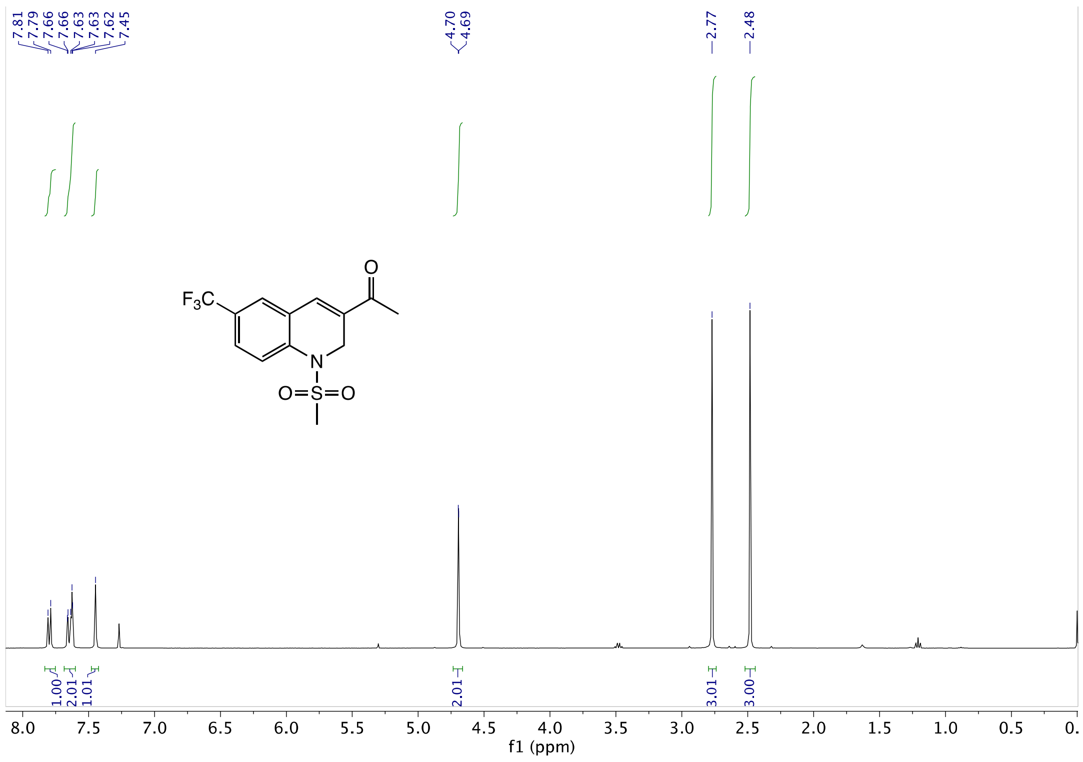

¹³C NMR for 1-(1-(methylsulfonyl)-6-(trifluoromethyl)-1,2-dihydroquinolin-3-yl)ethan-1-one (40)

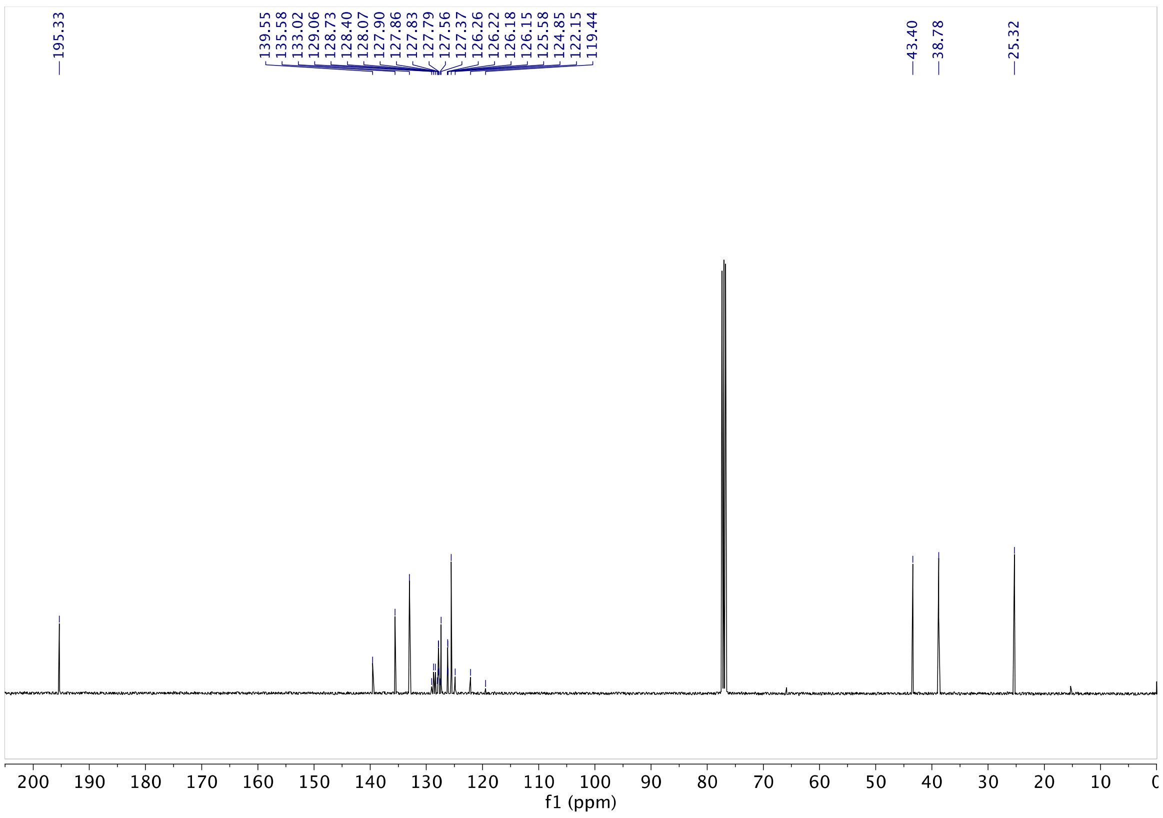

^{1}H NMR of 1-(6,7-difluoro-1-(methylsulfonyl)-1, 2-dihydroquinolin-3-yl)ethan-1-one (41)

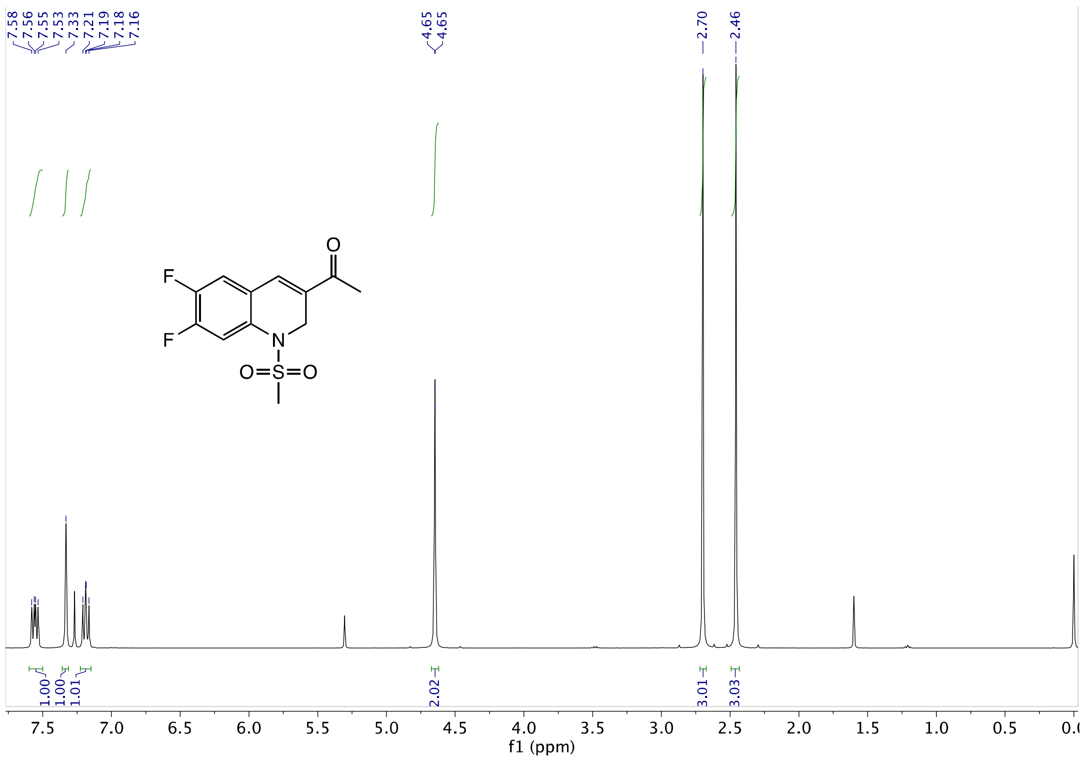

^{13}C NMR of 1-(6,7-difluoro-1-(methylsulfonyl)-1,2-dihydroquinolin-3-yl)ethan-1-one (41)

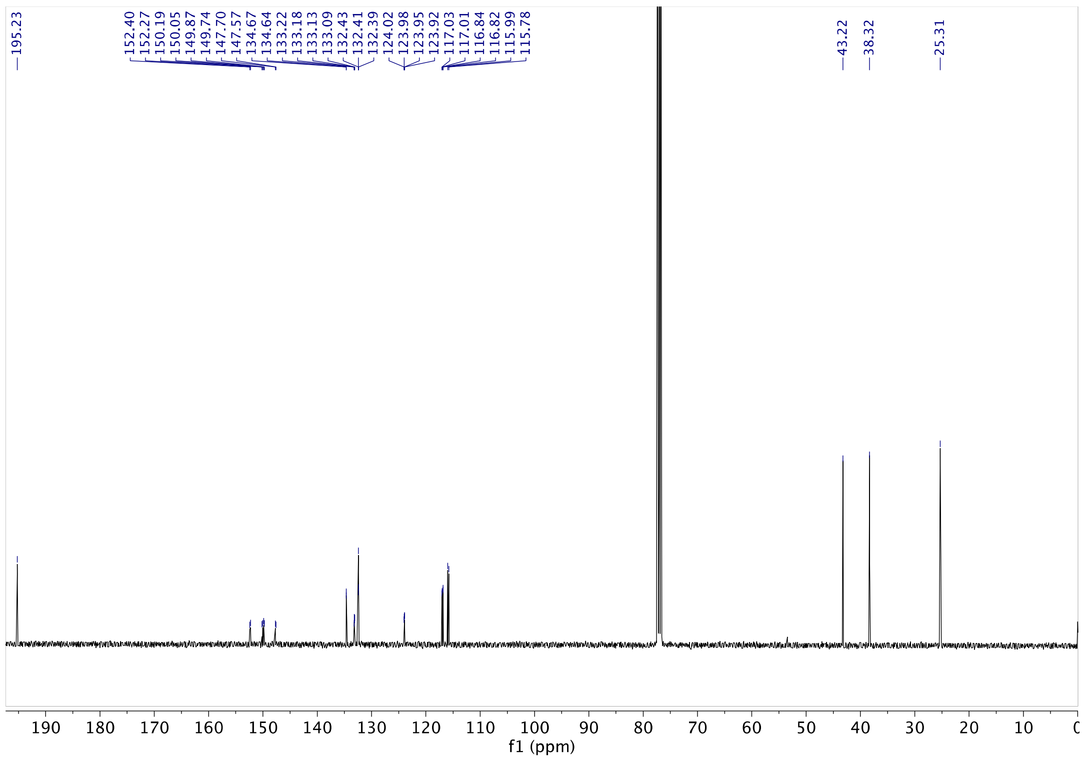

¹H for 1-(1-(methylsulfonyl)-1,2-dihydro-1,8-naphthyridin-3-yl)ethan-1-one (42)

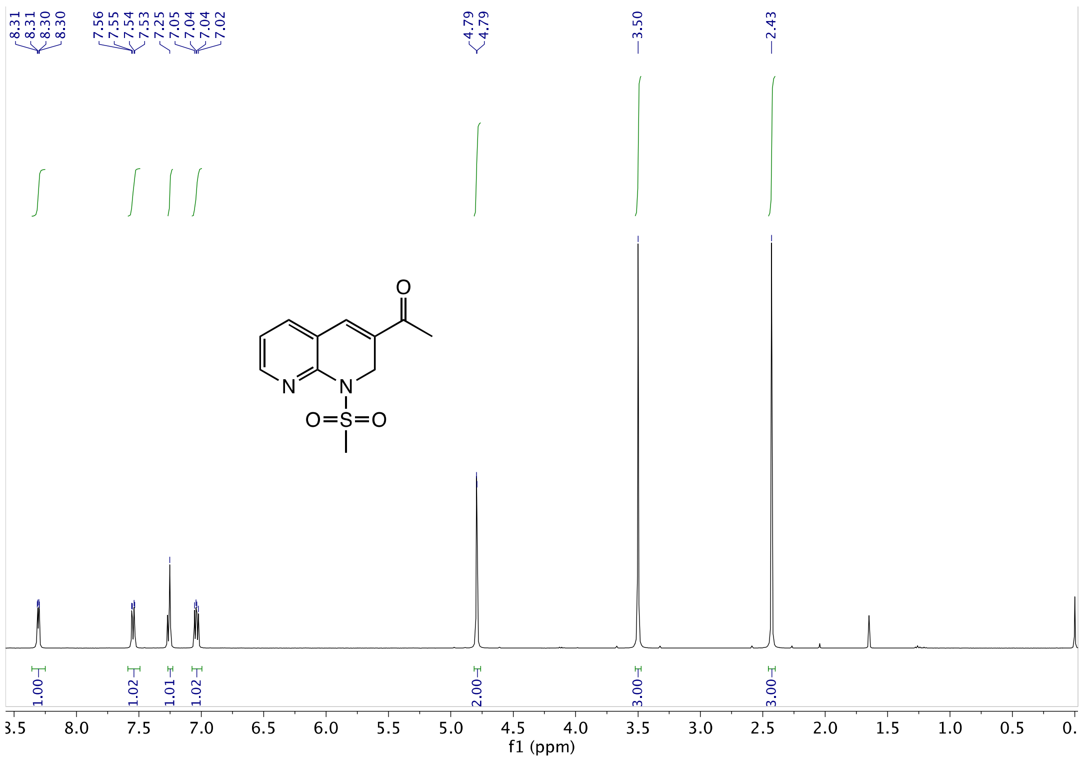

¹³C for 1-(1-(methylsulfonyl)-1,2-dihydro-1,8-naphthyridin-3-yl)ethan-1-one (42)

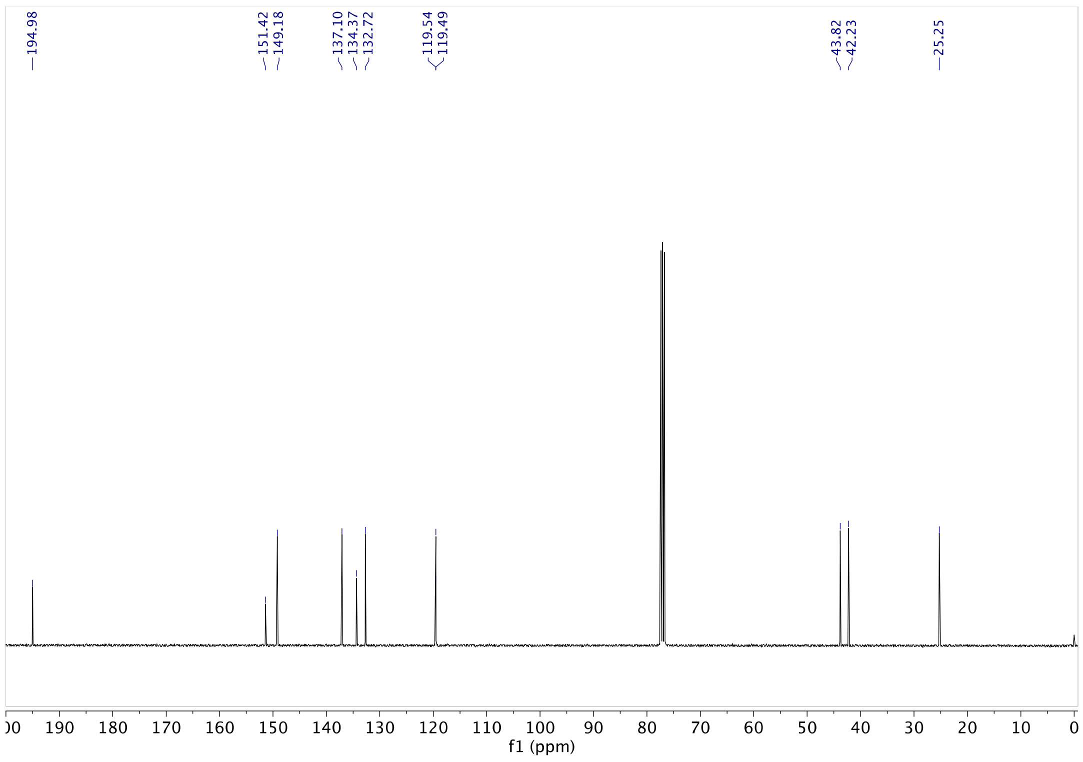

^{1}H NMR or ethyl 6-nitroquinoline-3-carboxylate (43 from 28-Ms)

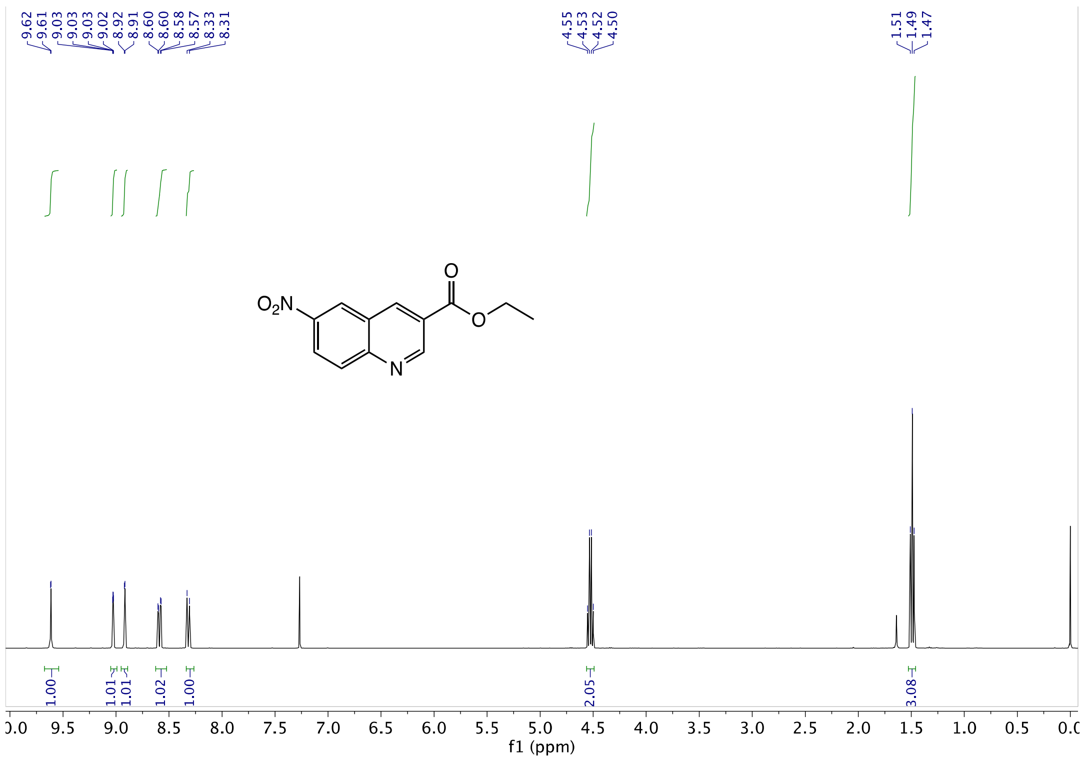

[13]C for ethyl 6-nitroquinoline-3-carboxylate (43 from 28-Ms)

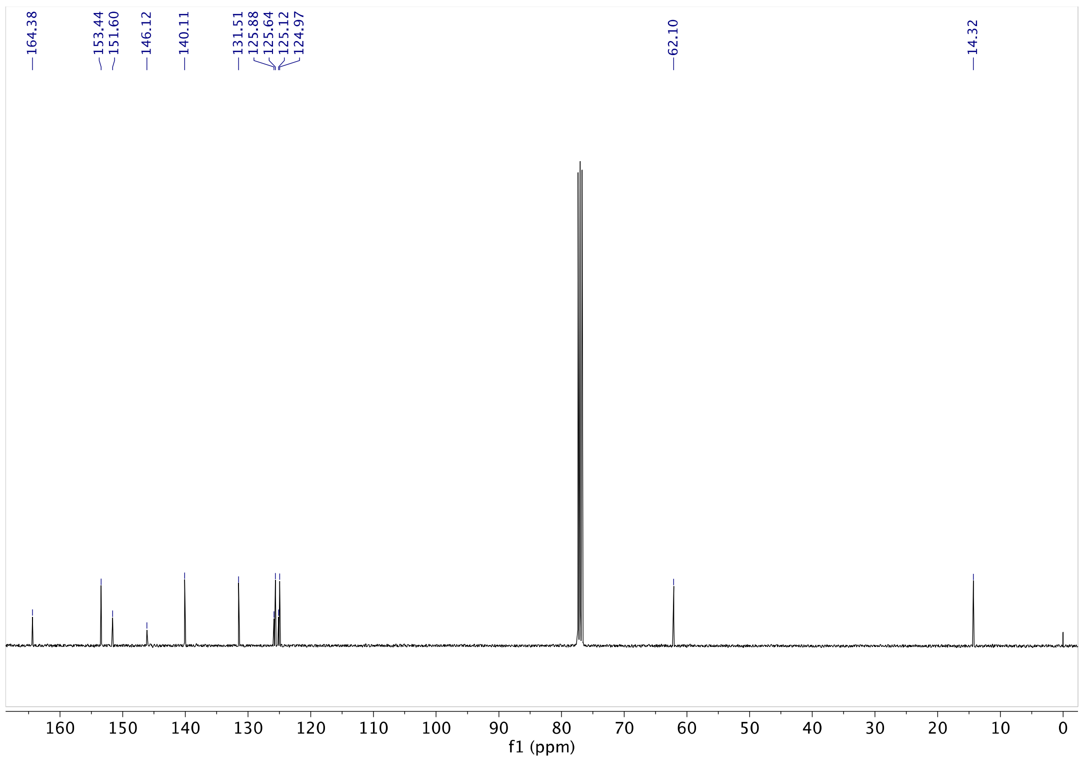

¹H NMR for ethyl 6-nitroquinoline-3-carboxylate (43 from 37-Ts)

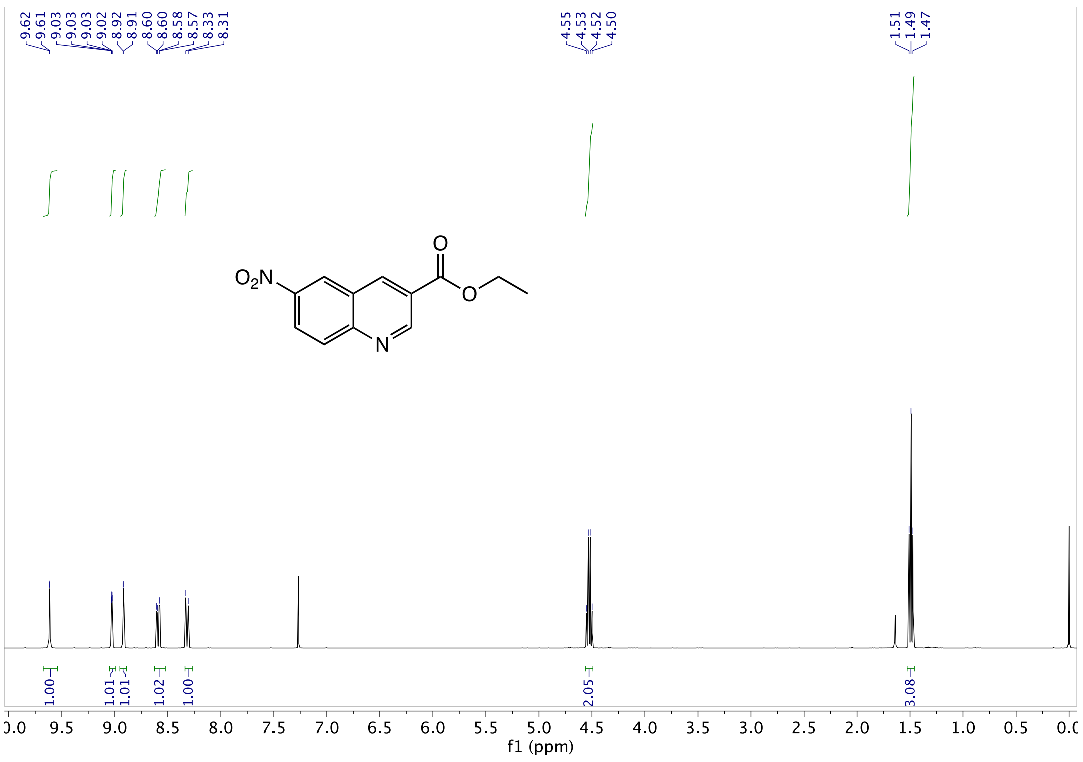

¹³C NMR or ethyl 6-nitroquinoline-3-carboxylate (43 from 37-Ts)

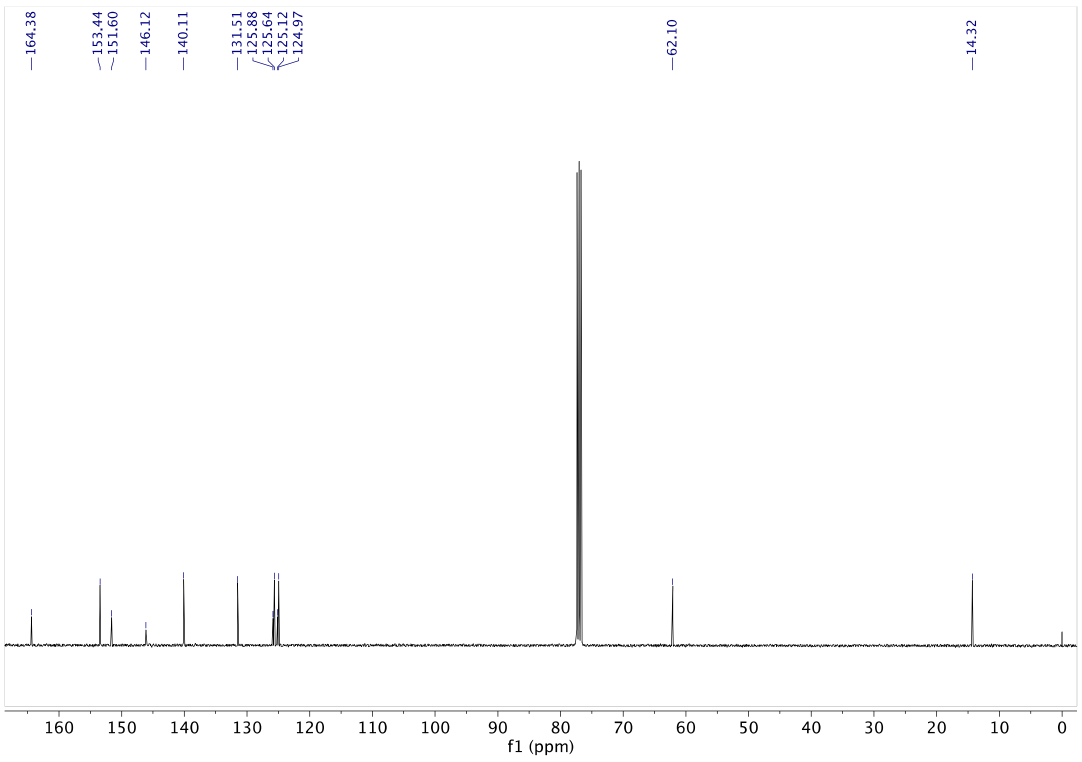

^{1}H NMR for ethyl 6-cyanoquinoline-3-carboxylate (44)

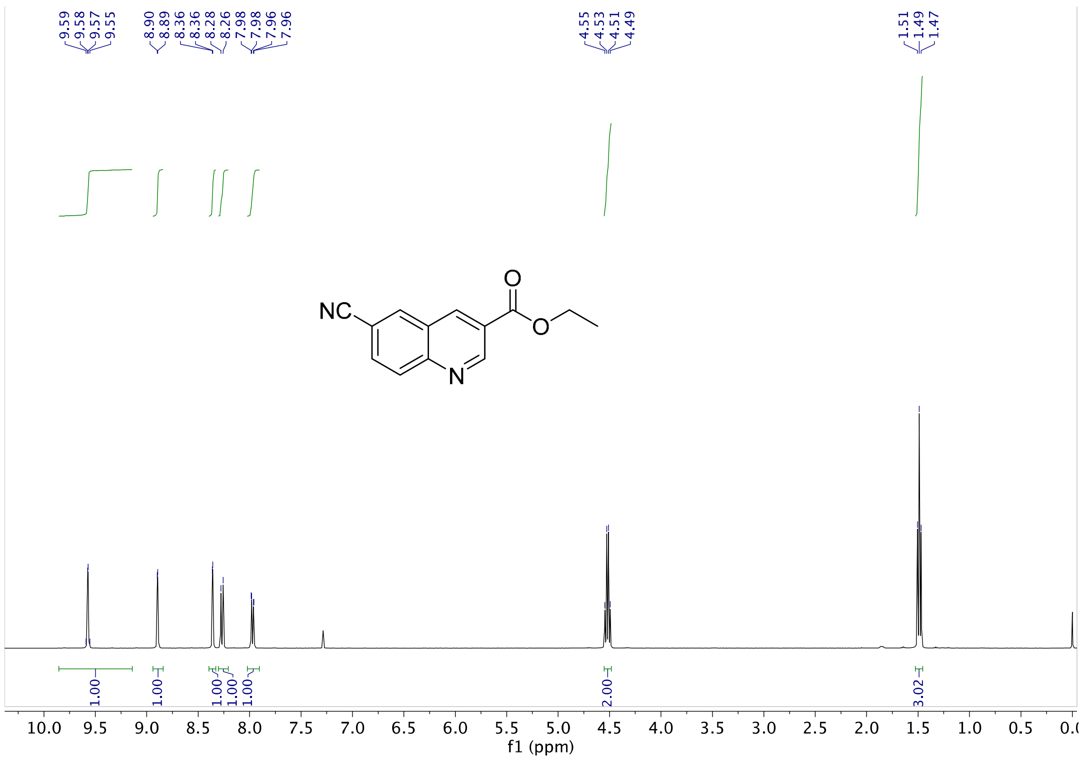

^{13}C NMR for ethyl 6-isocyanoquinoline-3-carboxylate (44)

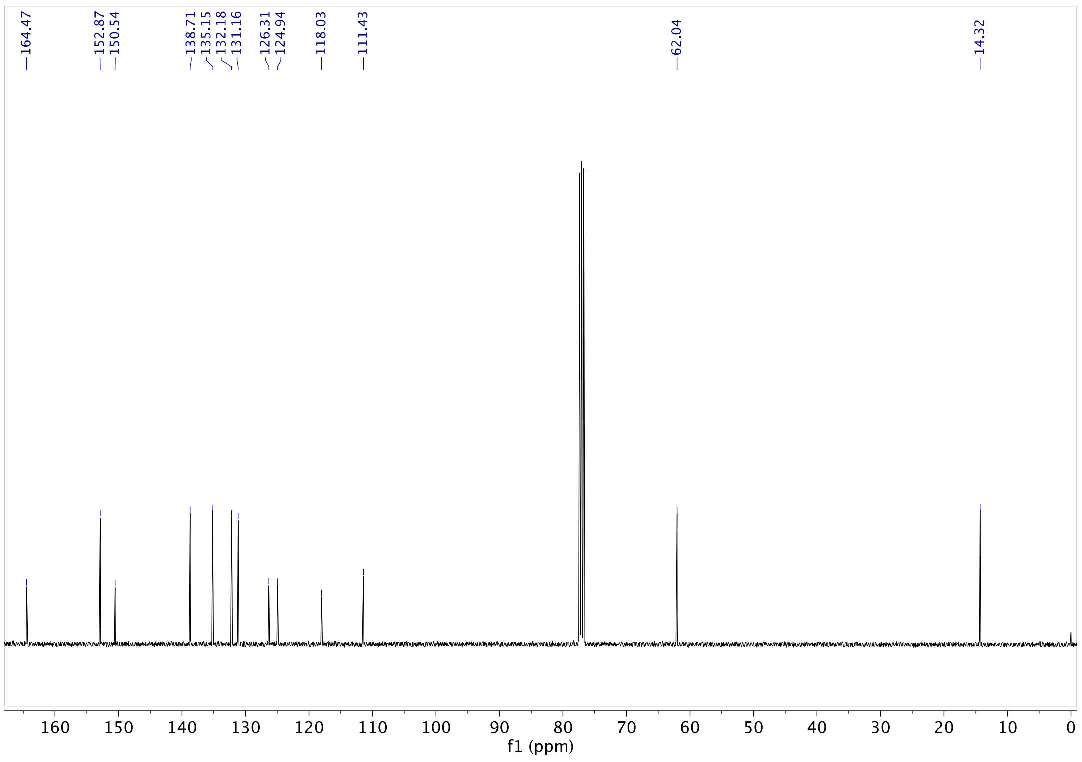

^{1}H NMR for ethyl 6-(trifluoromethyl)quinoline-3-carboxylate (45)

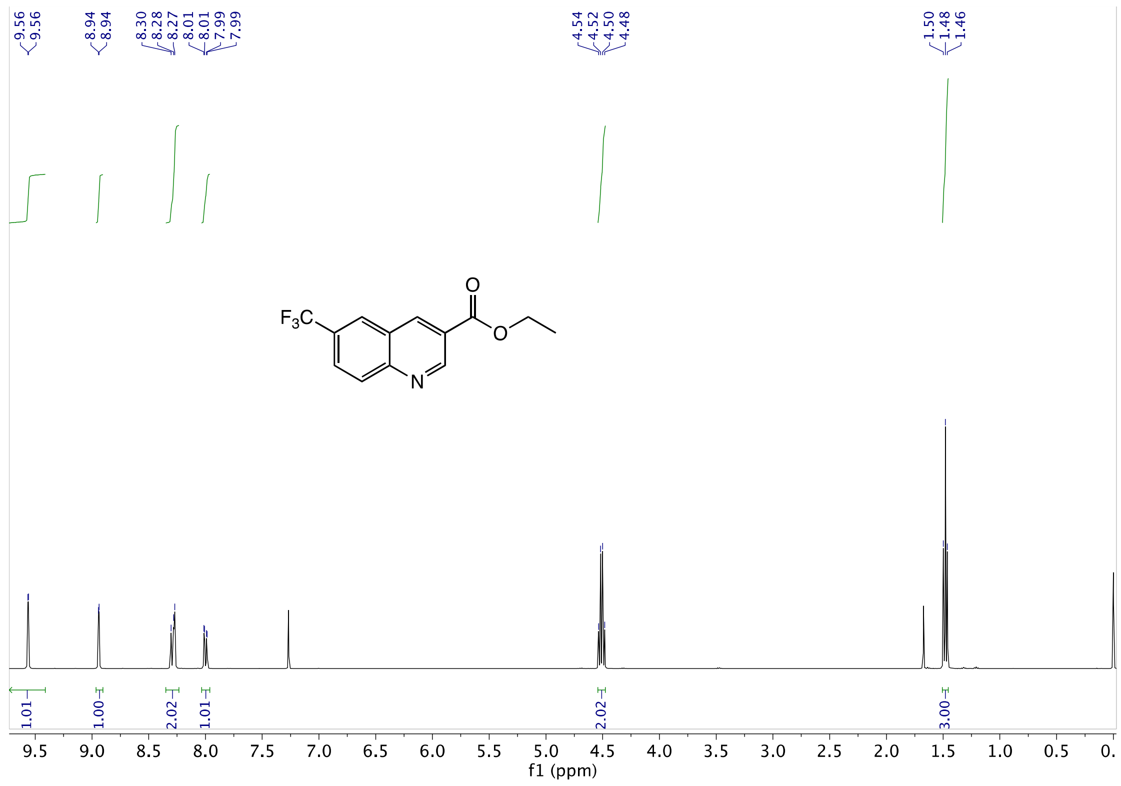

^{13}C NMR for ethyl 6-(trifluoromethyl)quinoline-3-carboxylate (45)

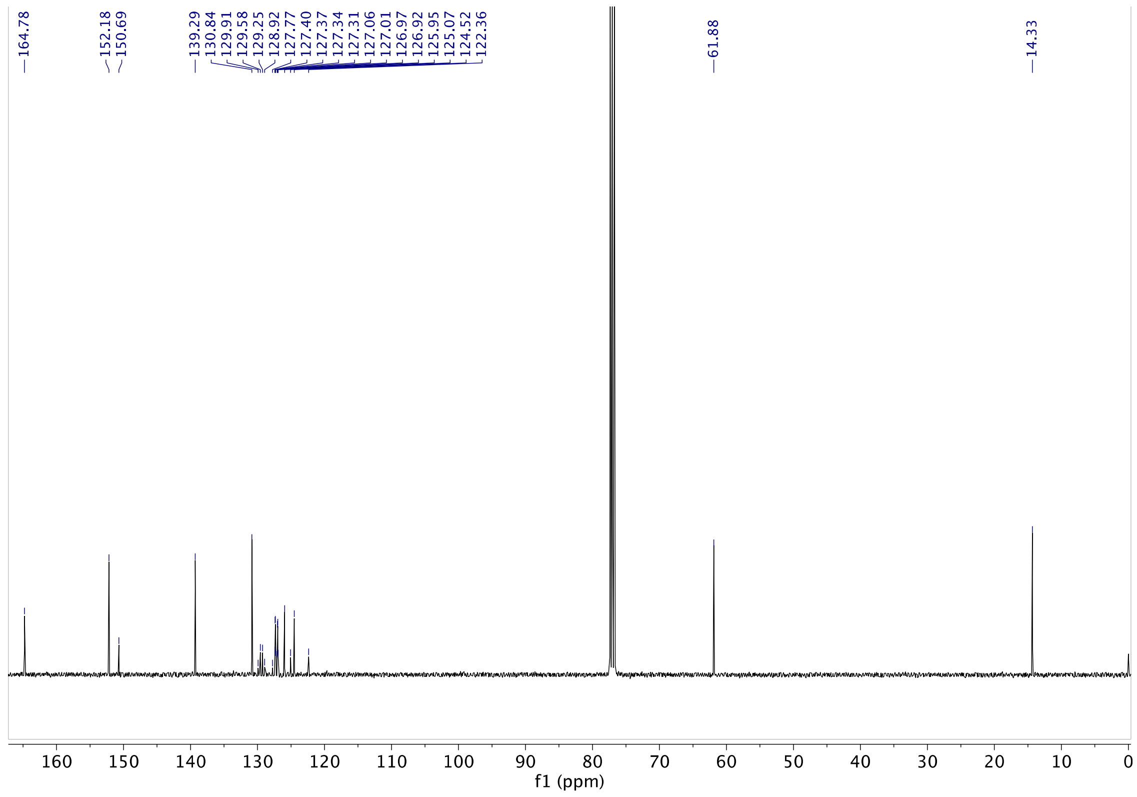

^{1}H NMR for ethyl 6-chloroquinoline-3-carboxylate (46)

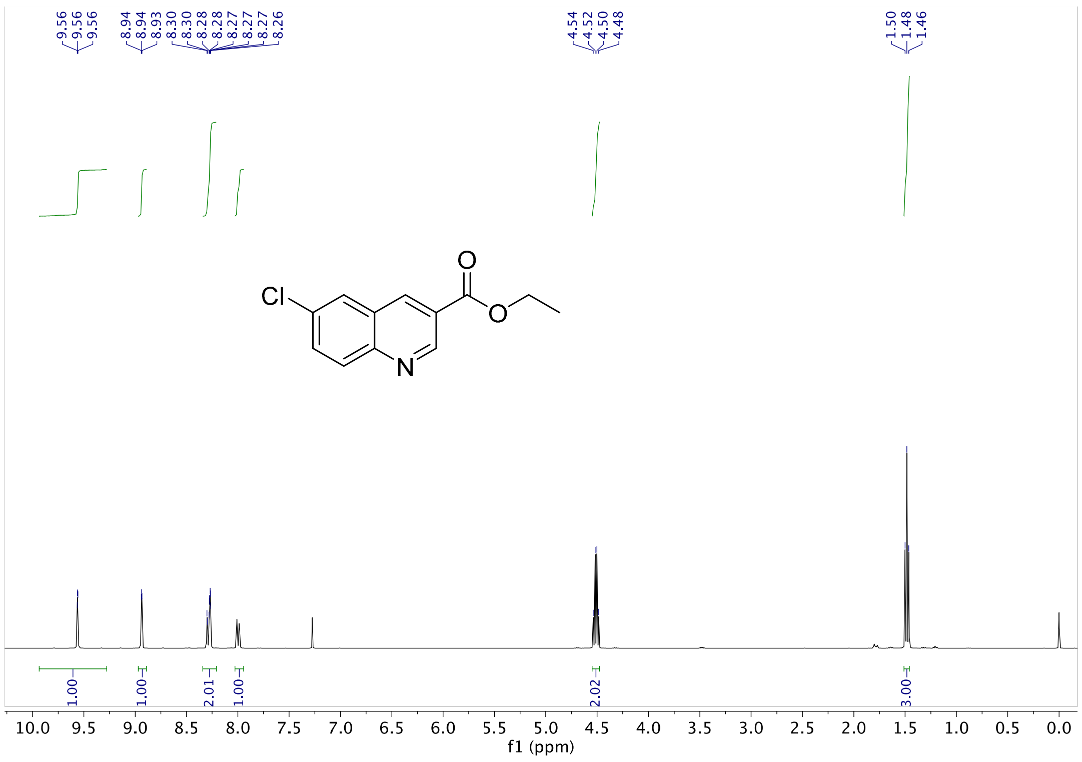

^{13}C NMR for ethyl 6-chloroquinoline-3-carboxylate (46)

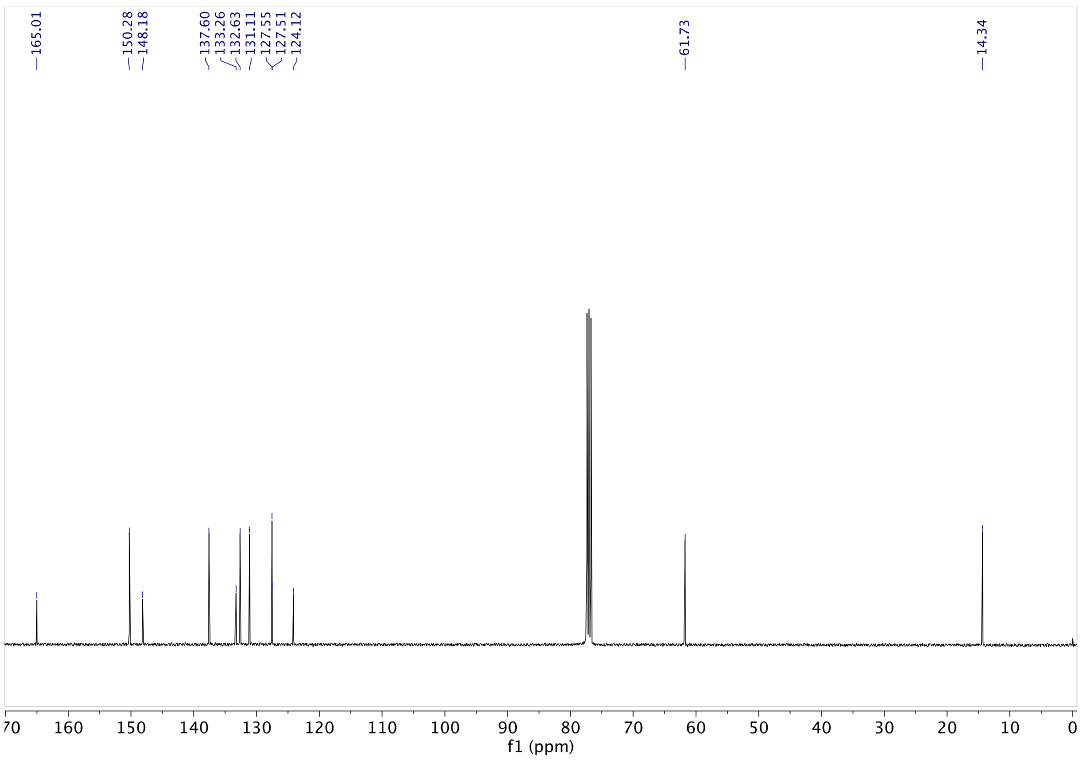

^{1}H NMR for 6-nitroquinoline-3-carbonitrile (48)

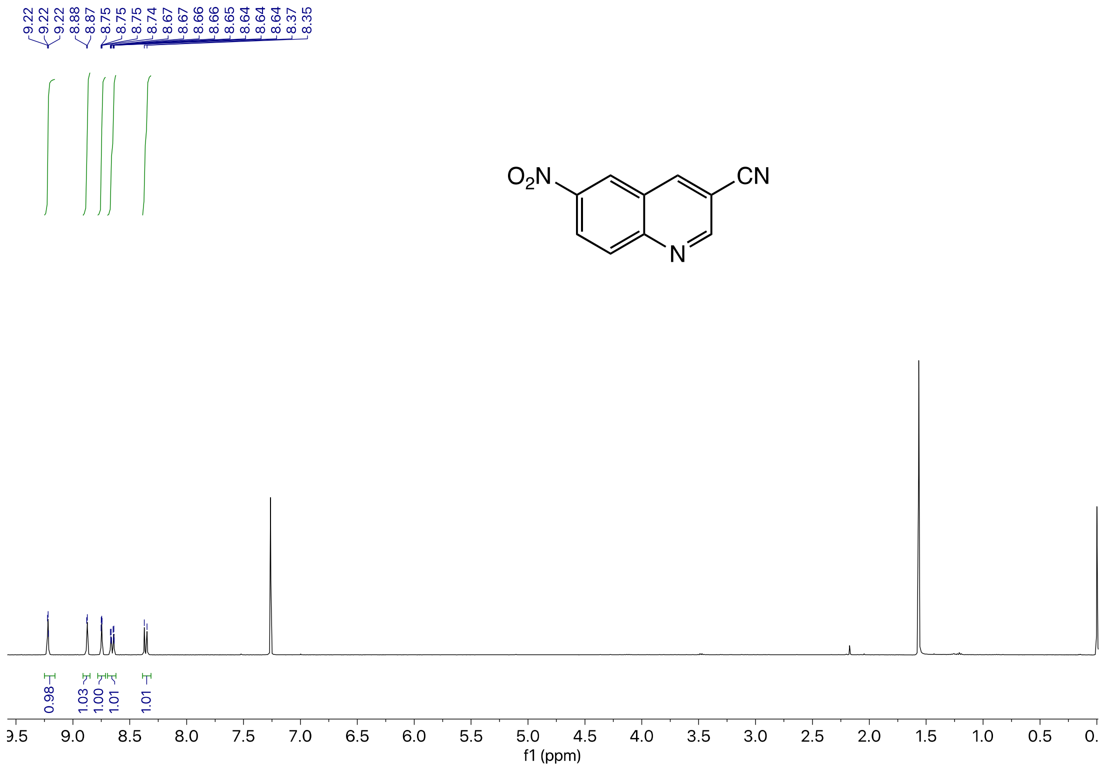

^{13}C NMR for 6-nitroquinoline-3-carbonitrile (48)

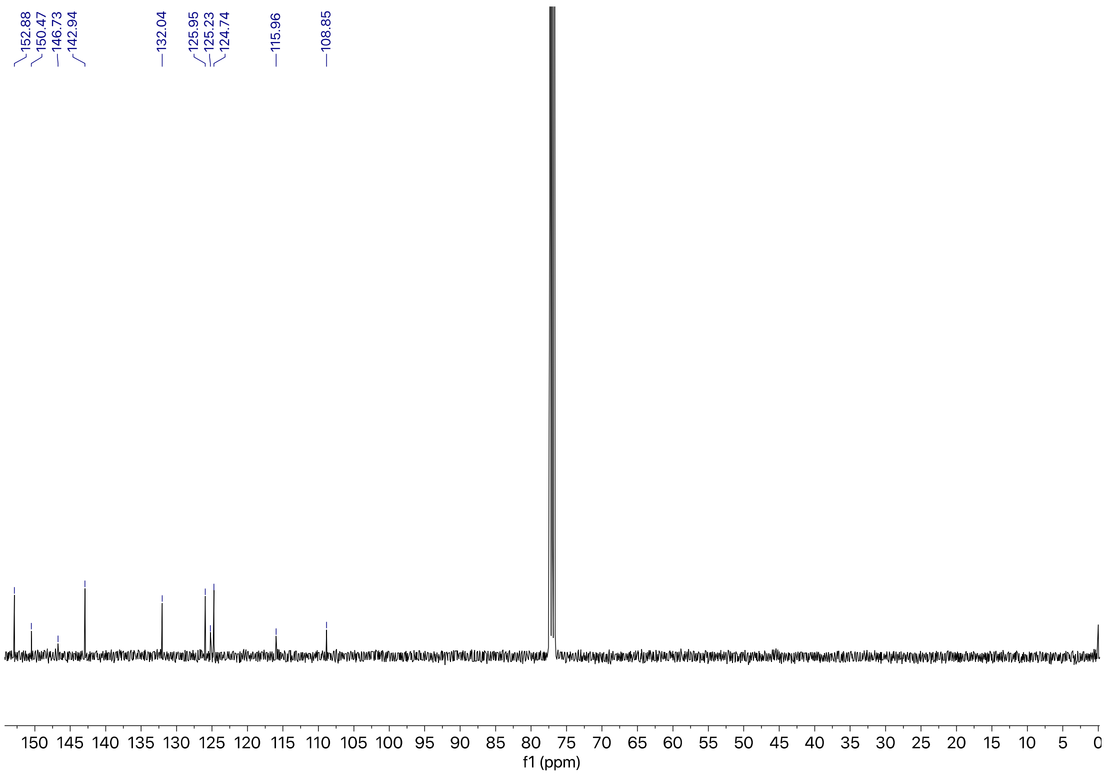

^{1}H NMR for ethyl 7-(methylsulfonyl)-6-(phenethylamino)quinoline-3-carboxylate (49)

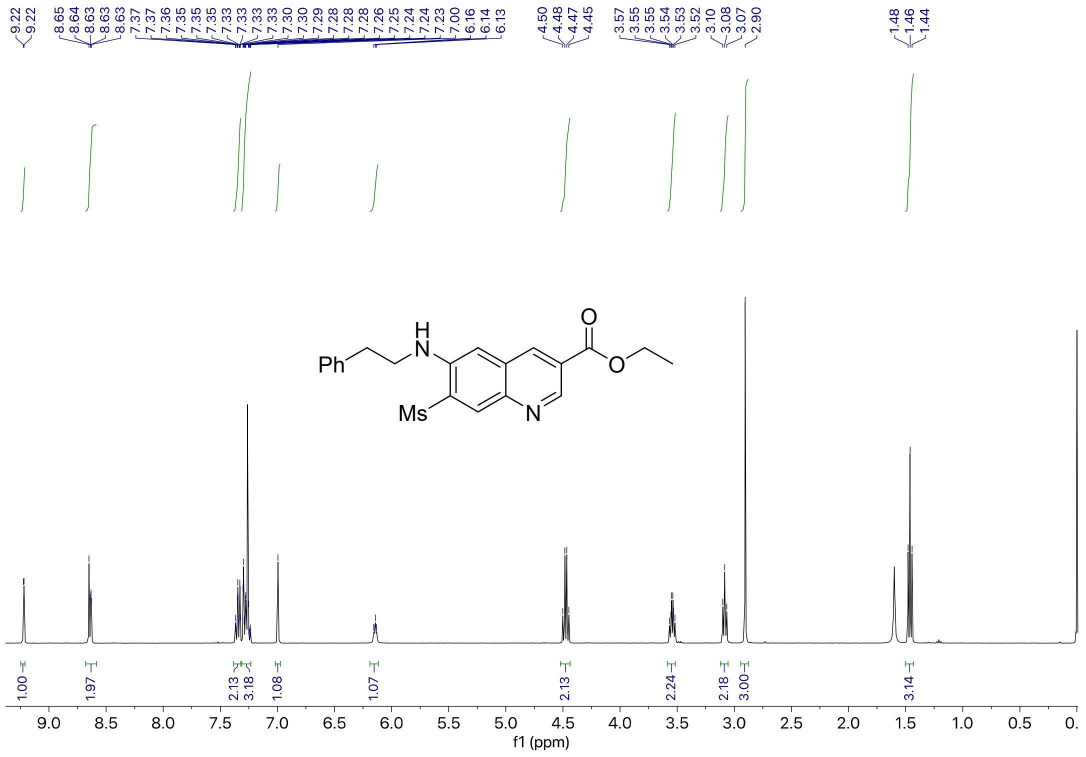

^{13}C NMR for ethyl 7-(methylsulfonyl)-6-(phenethylamino)quinoline-3-carboxylate (49)

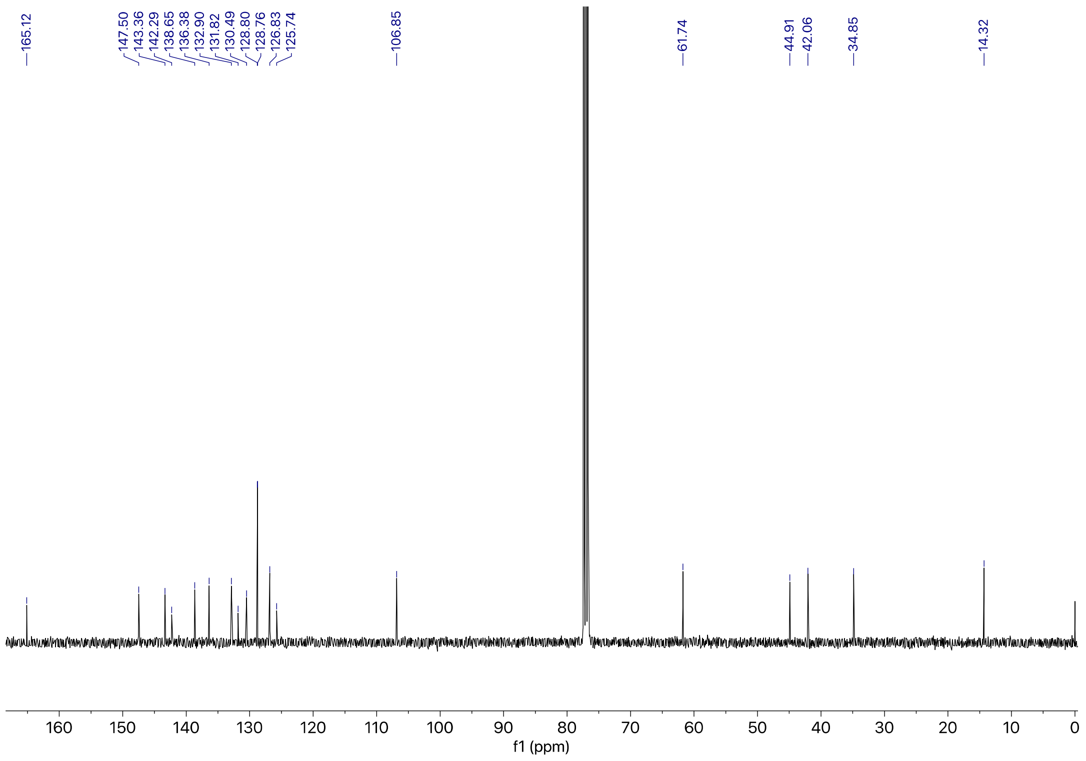

X-Ray Structure Determination for Compound 49

(CCDC 2246638)

Thermal Ellipsoid Plot Structure

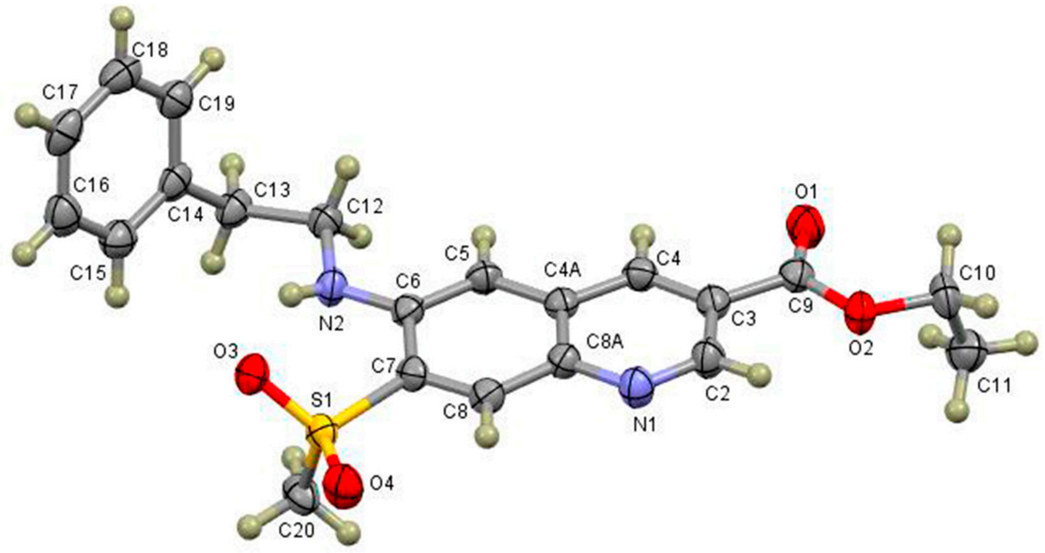

Packing Diagram

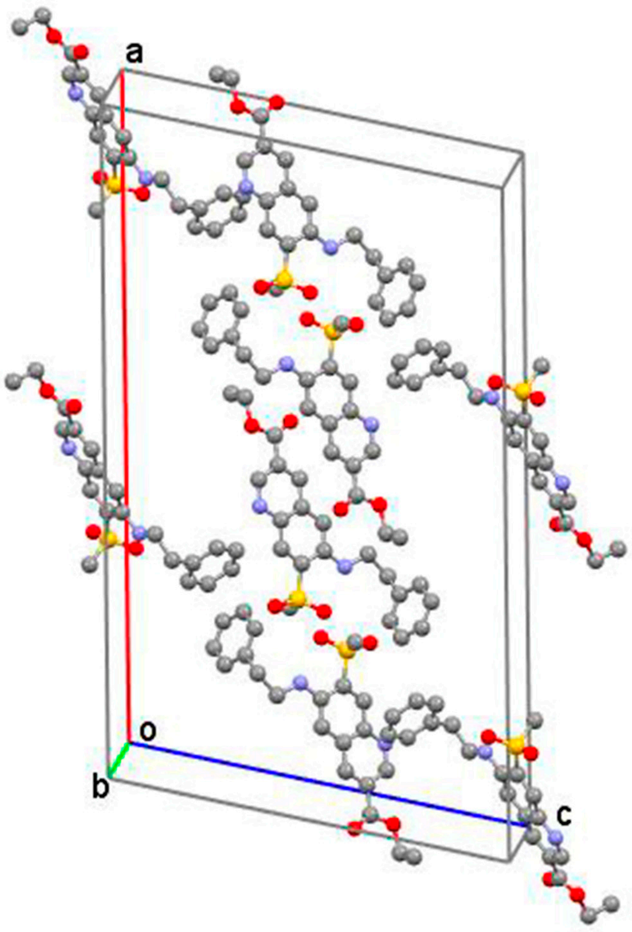

Comment

The displacement ellipsoids were drawn at the 50% probability level.

Experimental

A green, needle-shaped crystal of dimensions 0.046 x 0.122 x 0.252 mm was selected for structural analysis. Intensity data for this compound were collected using a D8 Quest κ-geometry diffractometer with a Bruker Photon II cmos area detector (1,2) and an Incoatec Iμs microfocus Mo Kα source (λ = 0.71073 Å). The sample was cooled to 100(2) K. Cell parameters were determined from a least-squares fit of 9733 peaks in the range 2.36 < θ < 25.63°. A total of 72607 data were measured in the range 2.364 < θ < 25.719° using φ and ω oscillation frames. The data were corrected for absorption by the empirical method (3) giving minimum and maximum transmission factors of 0.6364 and 0.6831. The data were merged to form a set of 3639 independent data with R(int) = 0.0998 and a coverage of 99.9 %.

The monoclinic space group *C*2/*c* was determined by systematic absences and statistical tests and verified by subsequent refinement. The structure was solved by dual-space methods and refined by full-matrix least-squares methods on F^2 (4,5). The positions of hydrogens bonded to carbons were initially determined by geometry and were refined using a riding model. The hydrogen bonded to N1 was located on a difference map, and its position was refined independently. Non-hydrogen atoms were refined with anisotropic displacement parameters. Hydrogen atom displacement parameters were set to 1.2 (1.5 for methyl) times the isotropic equivalent displacement parameters of the bonded atoms. A total of 259 parameters were refined against 3639 data to give $wR(F^2) = 0.1036$ and S = 1.006 for weights of $w = 1/[\sigma^2(F^2) + (0.0600\ P)^2 + 4.5000\ P]$ where $P = [F_o^2 + 2F_c^2] / 3$.

The final R(*F*) was 0.0361 for the 3315 observed, $[F > 4\sigma(F)]$, data. The largest shift/s.u. was 0.001 in the final refinement cycle. The final difference map had maxima and minima of 0.435 and -0.418 e/Å^3, respectively.

Acknowledgment

The authors thank the National Science Foundation (grant CHE-1726630) and the University of Oklahoma for funds to purchase of the X-ray instrument and computers. This structure was determined by Douglas R. Powell.

References

1. Data Collection: APEX3 (2018) Bruker Inc., Madison, Wisconsin, USA.
2. Data Reduction: SAINT (2016) Bruker Inc., Madison, Wisconsin, USA.
3. L. Krause, R. Herbst-Irmer, G. M. Sheldrick, and D. Stalke (2015). *J. Appl. Cryst., 48*, 3-10.
4. G. M. Sheldrick (2015). *Acta Cryst., A71*, 3-8.
5. G. M. Sheldrick (2015). *Acta Cryst., C71*, 3-8.

Table 1. Crystal data and structure refinement for Compound **49** (CCDC 2246638)

Empirical formula	$C_{21} H_{22} N_2 O_4 S$	
Formula weight	398.46	
Crystal system	monoclinic	
Space group	*C*2/*c*	
Unit cell dimensions	*a* = 34.920(5) Å	α= 90°
	b = 5.4677(6) Å	β= 99.312(5)°
	c = 20.235(3) Å	γ= 90°
Volume	3812.6(9) $Å^3$	
Z, Z'	8, 1	
Density (calculated)	1.388 Mg/m^3	
Wavelength	0.71073 Å	
Temperature	100(2) K	
F(000)	1680	
Absorption coefficient	0.201 mm^{-1}	
Absorption correction	semi-empirical from equivalents	
Max. and min. transmission	0.6831 and 0.6364	
Theta range for data collection	2.364 to 25.719°	
Reflections collected	72607	
Independent reflections	3639 [R(int) = 0.0998]	
Data / restraints / parameters	3639 / 0 / 259	
$wR(F^2$ all data)	*wR*2 = 0.1036	
R(*F* obsd data)	*R*1 = 0.0361	
Goodness-of-fit on F^2	1.006	
Observed data [I > 2σ(I)]	3315	
Extinction coefficient	0.0025(4)	
Largest and mean shift / s.u.	0.001 and 0.000	
Largest diff. peak and hole	0.435 and -0.418 e/$Å^3$	

$wR2 = \{ \Sigma [w(F_o^2 - F_c^2)^2] / \Sigma [w(F_o^2)^2] \}^{1/2}$

$R1 = \Sigma ||F_o| - |F_c|| / \Sigma |F_o|$

Table 2. Atomic coordinates and equivalent isotropic displacement parameters for Cpd **49**. U(eq) is defined as one third of the trace of the orthogonalized U_{ij} tensor.

	x	y	z	U(eq)
S(1)	0.68967(2)	0.24226(6)	0.53419(2)	0.01600(14)
O(1)	0.46496(3)	0.8793(2)	0.58153(5)	0.0251(3)
O(2)	0.46690(3)	0.5995(2)	0.66407(5)	0.0208(3)
O(3)	0.69712(3)	0.1987(2)	0.46689(5)	0.0216(3)
O(4)	0.69527(3)	0.0419(2)	0.58111(5)	0.0246(3)
N(1)	0.56424(4)	0.2675(2)	0.62832(7)	0.0206(3)
N(2)	0.64874(4)	0.6195(2)	0.43607(6)	0.0187(3)
C(2)	0.53159(4)	0.3758(3)	0.63601(7)	0.0203(3)
C(3)	0.51615(4)	0.5829(3)	0.59856(7)	0.0179(3)
C(4)	0.53526(4)	0.6757(3)	0.55001(7)	0.0172(3)
C(4A)	0.57054(4)	0.5639(3)	0.53930(7)	0.0161(3)
C(5)	0.59186(4)	0.6482(3)	0.49020(7)	0.0173(3)
C(6)	0.62715(4)	0.5420(3)	0.48305(7)	0.0160(3)
C(7)	0.64155(4)	0.3511(3)	0.52960(7)	0.0162(3)
C(8)	0.62036(4)	0.2597(3)	0.57509(7)	0.0174(3)
C(8A)	0.58384(4)	0.3613(3)	0.58077(7)	0.0168(3)
C(9)	0.48005(4)	0.7058(3)	0.61223(7)	0.0178(3)
C(10)	0.43305(4)	0.7103(3)	0.68561(8)	0.0234(3)
C(11)	0.44433(5)	0.9253(3)	0.73100(8)	0.0269(4)
C(12)	0.63417(4)	0.8055(3)	0.38724(7)	0.0179(3)
C(13)	0.66405(4)	0.8629(3)	0.34205(7)	0.0198(3)
C(14)	0.67456(4)	0.6479(3)	0.30159(7)	0.0185(3)
C(15)	0.70996(4)	0.5272(3)	0.31898(7)	0.0216(3)
C(16)	0.71995(5)	0.3308(3)	0.28129(8)	0.0235(3)
C(17)	0.69441(5)	0.2507(3)	0.22566(8)	0.0232(4)
C(18)	0.65909(5)	0.3692(3)	0.20788(7)	0.0232(3)
C(19)	0.64938(4)	0.5665(3)	0.24508(7)	0.0206(3)
C(20)	0.71853(4)	0.4897(3)	0.56756(8)	0.0217(3)

Table 3. Bond lengths [Å] and angles [°] for Cpd **49**.

S(1)-O(4)	1.4421(11)	C(10)-C(11)	1.505(2)
S(1)-O(3)	1.4468(11)	C(10)-H(10A)	0.9900
S(1)-C(20)	1.7553(16)	C(10)-H(10B)	0.9900
S(1)-C(7)	1.7709(14)	C(11)-H(11A)	0.9800
O(1)-C(9)	1.2071(19)	C(11)-H(11B)	0.9800
O(2)-C(9)	1.3436(18)	C(11)-H(11C)	0.9800
O(2)-C(10)	1.4562(17)	C(12)-C(13)	1.5271(19)
N(1)-C(2)	1.316(2)	C(12)-H(12A)	0.9900
N(1)-C(8A)	1.3677(19)	C(12)-H(12B)	0.9900
N(2)-C(6)	1.3725(18)	C(13)-C(14)	1.512(2)
N(2)-C(12)	1.4514(19)	C(13)-H(13A)	0.9900
N(2)-H(2N)	0.85(2)	C(13)-H(13B)	0.9900
C(2)-C(3)	1.419(2)	C(14)-C(15)	1.395(2)
C(2)-H(2)	0.9500	C(14)-C(19)	1.398(2)
C(3)-C(4)	1.371(2)	C(15)-C(16)	1.394(2)
C(3)-C(9)	1.493(2)	C(15)-H(15)	0.9500
C(4)-C(4A)	1.423(2)	C(16)-C(17)	1.389(2)
C(4)-H(4)	0.9500	C(16)-H(16)	0.9500
C(4A)-C(5)	1.412(2)	C(17)-C(18)	1.388(2)
C(4A)-C(8A)	1.421(2)	C(17)-H(17)	0.9500
C(5)-C(6)	1.391(2)	C(18)-C(19)	1.389(2)
C(5)-H(5)	0.9500	C(18)-H(18)	0.9500
C(6)-C(7)	1.441(2)	C(19)-H(19)	0.9500
C(7)-C(8)	1.365(2)	C(20)-H(20A)	0.9800
C(8)-C(8A)	1.413(2)	C(20)-H(20B)	0.9800
C(8)-H(8)	0.9500	C(20)-H(20C)	0.9800
O(4)-S(1)-O(3)	117.96(7)	C(4)-C(3)-C(9)	119.32(13)
O(4)-S(1)-C(20)	108.90(7)	C(2)-C(3)-C(9)	121.28(13)
O(3)-S(1)-C(20)	108.03(7)	C(3)-C(4)-C(4A)	118.96(14)
O(4)-S(1)-C(7)	108.43(7)	C(3)-C(4)-H(4)	120.5
O(3)-S(1)-C(7)	108.63(6)	C(4A)-C(4)-H(4)	120.5
C(20)-S(1)-C(7)	104.00(7)	C(5)-C(4A)-C(8A)	120.71(13)
C(9)-O(2)-C(10)	116.76(12)	C(5)-C(4A)-C(4)	122.35(13)
C(2)-N(1)-C(8A)	117.12(13)	C(8A)-C(4A)-C(4)	116.94(13)
C(6)-N(2)-C(12)	121.10(12)	C(6)-C(5)-C(4A)	120.92(13)
C(6)-N(2)-H(2N)	112.7(12)	C(6)-C(5)-H(5)	119.5
C(12)-N(2)-H(2N)	119.8(12)	C(4A)-C(5)-H(5)	119.5
N(1)-C(2)-C(3)	123.87(14)	N(2)-C(6)-C(5)	122.57(13)
N(1)-C(2)-H(2)	118.1	N(2)-C(6)-C(7)	120.24(13)
C(3)-C(2)-H(2)	118.1	C(5)-C(6)-C(7)	117.12(13)
C(4)-C(3)-C(2)	119.38(13)	C(8)-C(7)-C(6)	122.39(13)

C(8)-C(7)-S(1)	117.06(11)	C(14)-C(13)-H(13A)	108.6
C(6)-C(7)-S(1)	120.37(11)	C(12)-C(13)-H(13A)	108.6
C(7)-C(8)-C(8A)	120.39(13)	C(14)-C(13)-H(13B)	108.6
C(7)-C(8)-H(8)	119.8	C(12)-C(13)-H(13B)	108.6
C(8A)-C(8)-H(8)	119.8	H(13A)-C(13)-H(13B)	107.6
N(1)-C(8A)-C(8)	118.08(13)	C(15)-C(14)-C(19)	118.05(14)
N(1)-C(8A)-C(4A)	123.70(13)	C(15)-C(14)-C(13)	120.95(14)
C(8)-C(8A)-C(4A)	118.12(13)	C(19)-C(14)-C(13)	120.98(14)
O(1)-C(9)-O(2)	124.46(14)	C(16)-C(15)-C(14)	121.10(14)
O(1)-C(9)-C(3)	124.72(14)	C(16)-C(15)-H(15)	119.5
O(2)-C(9)-C(3)	110.82(12)	C(14)-C(15)-H(15)	119.5
O(2)-C(10)-C(11)	111.26(13)	C(17)-C(16)-C(15)	120.11(15)
O(2)-C(10)-H(10A)	109.4	C(17)-C(16)-H(16)	119.9
C(11)-C(10)-H(10A)	109.4	C(15)-C(16)-H(16)	119.9
O(2)-C(10)-H(10B)	109.4	C(18)-C(17)-C(16)	119.32(15)
C(11)-C(10)-H(10B)	109.4	C(18)-C(17)-H(17)	120.3
H(10A)-C(10)-H(10B)	108.0	C(16)-C(17)-H(17)	120.3
C(10)-C(11)-H(11A)	109.5	C(17)-C(18)-C(19)	120.46(15)
C(10)-C(11)-H(11B)	109.5	C(17)-C(18)-H(18)	119.8
H(11A)-C(11)-H(11B)	109.5	C(19)-C(18)-H(18)	119.8
C(10)-C(11)-H(11C)	109.5	C(18)-C(19)-C(14)	120.95(15)
H(11A)-C(11)-H(11C)	109.5	C(18)-C(19)-H(19)	119.5
H(11B)-C(11)-H(11C)	109.5	C(14)-C(19)-H(19)	119.5
N(2)-C(12)-C(13)	110.70(12)	S(1)-C(20)-H(20A)	109.5
N(2)-C(12)-H(12A)	109.5	S(1)-C(20)-H(20B)	109.5
C(13)-C(12)-H(12A)	109.5	H(20A)-C(20)-H(20B)	109.5
N(2)-C(12)-H(12B)	109.5	S(1)-C(20)-H(20C)	109.5
C(13)-C(12)-H(12B)	109.5	H(20A)-C(20)-H(20C)	109.5
H(12A)-C(12)-H(12B)	108.1	H(20B)-C(20)-H(20C)	109.5
C(14)-C(13)-C(12)	114.44(12)		

Table 4. Anisotropic displacement parameters ($Å^2 x 10^3$) for Cpd **49** The anisotropic displacement factor exponent takes the form: $-2 \pi^2 [h^2 a^{*2} U_{11} + ... + 2 h k a^* b^* U_{12}]$

	U_{11}	U_{22}	U_{33}	U_{23}	U_{13}	U_{12}
S(1)	16(1)	15(1)	17(1)	-1(1)	4(1)	4(1)
O(1)	22(1)	28(1)	26(1)	5(1)	7(1)	8(1)
O(2)	19(1)	23(1)	23(1)	0(1)	10(1)	3(1)
O(3)	22(1)	24(1)	20(1)	-6(1)	6(1)	4(1)
O(4)	25(1)	22(1)	28(1)	6(1)	7(1)	9(1)
N(1)	20(1)	21(1)	22(1)	4(1)	7(1)	2(1)
N(2)	18(1)	21(1)	18(1)	2(1)	7(1)	4(1)
C(2)	19(1)	23(1)	21(1)	3(1)	7(1)	1(1)
C(3)	15(1)	21(1)	18(1)	-2(1)	3(1)	-1(1)
C(4)	17(1)	18(1)	16(1)	0(1)	1(1)	1(1)
C(4A)	16(1)	17(1)	15(1)	-2(1)	2(1)	0(1)
C(5)	18(1)	18(1)	15(1)	3(1)	2(1)	1(1)
C(6)	17(1)	17(1)	14(1)	-1(1)	3(1)	-1(1)
C(7)	16(1)	16(1)	16(1)	-3(1)	3(1)	3(1)
C(8)	20(1)	16(1)	17(1)	0(1)	3(1)	2(1)
C(8A)	18(1)	17(1)	16(1)	0(1)	4(1)	0(1)
C(9)	16(1)	20(1)	18(1)	-2(1)	3(1)	-1(1)
C(10)	16(1)	28(1)	28(1)	-2(1)	10(1)	2(1)
C(11)	26(1)	31(1)	24(1)	-3(1)	5(1)	7(1)
C(12)	17(1)	20(1)	17(1)	2(1)	4(1)	1(1)
C(13)	22(1)	21(1)	18(1)	0(1)	6(1)	-4(1)
C(14)	22(1)	20(1)	16(1)	2(1)	8(1)	-4(1)
C(15)	22(1)	25(1)	18(1)	2(1)	4(1)	-5(1)
C(16)	22(1)	26(1)	24(1)	6(1)	8(1)	2(1)
C(17)	33(1)	19(1)	20(1)	3(1)	12(1)	0(1)
C(18)	29(1)	25(1)	16(1)	0(1)	4(1)	-4(1)
C(19)	21(1)	23(1)	19(1)	3(1)	4(1)	-1(1)
C(20)	18(1)	22(1)	26(1)	-6(1)	3(1)	1(1)

Table 5. Hydrogen coordinates and isotropic displacement parameters for Cpd **49**.

	x	y	z	U(eq)
H(2N)	0.6641(5)	0.510(4)	0.4260(9)	0.022
H(2)	0.517518	0.311571	0.668535	0.024
H(4)	0.525113	0.812399	0.523937	0.021
H(5)	0.581962	0.779337	0.461645	0.021
H(8)	0.630239	0.127101	0.603075	0.021
H(10A)	0.419701	0.586828	0.709520	0.028
H(10B)	0.414709	0.764871	0.645836	0.028
H(11A)	0.454494	1.056325	0.705692	0.040
H(11B)	0.464343	0.874954	0.768205	0.040
H(11C)	0.421514	0.984598	0.748682	0.040
H(12A)	0.628210	0.956171	0.410745	0.021
H(12B)	0.609870	0.747482	0.359609	0.021
H(13A)	0.653766	0.995601	0.310866	0.024
H(13B)	0.687952	0.924490	0.370169	0.024
H(15)	0.727538	0.579674	0.357153	0.026
H(16)	0.744275	0.251698	0.293655	0.028
H(17)	0.701050	0.116126	0.200049	0.028
H(18)	0.641441	0.314974	0.169999	0.028
H(19)	0.625273	0.647405	0.231926	0.025
H(20A)	0.745986	0.447716	0.569721	0.033
H(20B)	0.713330	0.525966	0.612707	0.033
H(20C)	0.712375	0.633459	0.538898	0.033

Table 6. Torsion angles [°] for Cpd **49**.

C(8A)-N(1)-C(2)-C(3)	-0.6(2)	C(2)-N(1)-C(8A)-C(4A)	-1.1(2)
N(1)-C(2)-C(3)-C(4)	1.7(2)	C(7)-C(8)-C(8A)-N(1)	-178.29(13)
N(1)-C(2)-C(3)-C(9)	-176.63(14)	C(7)-C(8)-C(8A)-C(4A)	-1.7(2)
C(2)-C(3)-C(4)-C(4A)	-1.1(2)	C(5)-C(4A)-C(8A)-N(1)	-178.67(14)
C(9)-C(3)-C(4)-C(4A)	177.30(13)	C(4)-C(4A)-C(8A)-N(1)	1.6(2)
C(3)-C(4)-C(4A)-C(5)	179.83(13)	C(5)-C(4A)-C(8A)-C(8)	4.9(2)
C(3)-C(4)-C(4A)-C(8A)	-0.4(2)	C(4)-C(4A)-C(8A)-C(8)	-174.81(13)
C(8A)-C(4A)-C(5)-C(6)	-2.7(2)	C(10)-O(2)-C(9)-O(1)	-2.6(2)
C(4)-C(4A)-C(5)-C(6)	177.04(13)	C(10)-O(2)-C(9)-C(3)	176.41(12)
C(12)-N(2)-C(6)-C(5)	-5.1(2)	C(4)-C(3)-C(9)-O(1)	3.1(2)
C(12)-N(2)-C(6)-C(7)	177.84(13)	C(2)-C(3)-C(9)-O(1)	-178.50(15)
C(4A)-C(5)-C(6)-N(2)	-179.77(13)	C(4)-C(3)-C(9)-O(2)	-175.93(13)
C(4A)-C(5)-C(6)-C(7)	-2.6(2)	C(2)-C(3)-C(9)-O(2)	2.44(19)
N(2)-C(6)-C(7)-C(8)	-176.79(14)	C(9)-O(2)-C(10)-C(11)	-82.17(16)
C(5)-C(6)-C(7)-C(8)	6.0(2)	C(6)-N(2)-C(12)-C(13)	178.64(13)
N(2)-C(6)-C(7)-S(1)	8.29(19)	N(2)-C(12)-C(13)-C(14)	61.08(17)
C(5)-C(6)-C(7)-S(1)	-168.91(11)	C(12)-C(13)-C(14)-C(15)	-105.01(16)
O(4)-S(1)-C(7)-C(8)	8.80(13)	C(12)-C(13)-C(14)-C(19)	76.00(18)
O(3)-S(1)-C(7)-C(8)	138.13(11)	C(19)-C(14)-C(15)-C(16)	-0.1(2)
C(20)-S(1)-C(7)-C(8)	-106.99(12)	C(13)-C(14)-C(15)-C(16)	-179.16(13)
O(4)-S(1)-C(7)-C(6)	-176.03(11)	C(14)-C(15)-C(16)-C(17)	-0.5(2)
O(3)-S(1)-C(7)-C(6)	-46.69(13)	C(15)-C(16)-C(17)-C(18)	0.5(2)
C(20)-S(1)-C(7)-C(6)	68.19(13)	C(16)-C(17)-C(18)-C(19)	0.2(2)
C(6)-C(7)-C(8)-C(8A)	-3.8(2)	C(17)-C(18)-C(19)-C(14)	-0.9(2)
S(1)-C(7)-C(8)-C(8A)	171.24(11)	C(15)-C(14)-C(19)-C(18)	0.9(2)
C(2)-N(1)-C(8A)-C(8)	175.34(13)	C(13)-C(14)-C(19)-C(18)	179.89(13)

Table 7. Hydrogen bonds for Cpd **49** [Å and °].

D-H...A	d(D-H)	d(H...A)	d(D...A)	<(DHA)
N(2)-H(2N)...O(3)	0.85(2)	2.14(2)	2.8627(17)	142.0(16)
C(5)-H(5)...O(1)#1	0.95	2.54	3.4331(18)	156.0
C(13)-H(13B)...O(3)#2	0.99	2.45	3.1847(19)	131.1
C(20)-H(20A)...O(3)#3	0.98	2.37	3.2998(19)	158.3
C(20)-H(20C)...O(4)#2	0.98	2.50	3.1502(19)	123.8

Symmetry transformations used to generate equivalent atoms:

#1 -x+1, -y+2, -z+1   #2 x, y+1, z   #3 -x+3/2, -y+1/2, -z+1
